# The science of child and adolescent mental health in Mozambique: A systematic review of prevalence estimates, measurement tools, and interventions

**DOI:** 10.1371/journal.pgph.0007513

**Published:** 2026-10-23

**Authors:** Helena Mutede Cutótua Daniel, Lauro Estivalete Marchionatti, Jessica Azevedo Veronesi, André Cardoso Campello, Luis Augusto Rohde, Caio Borba Casella, Peter Raucci, Afonso Mazine Tiago Fumo, Jair Mari, Giovanni Abrahão Salum, Lidia Gouveia, Zeina Mneimneh

**Affiliations:** 1 Stavros Niarchos Foundation (SNF) Global Center for Child and Adolescent Mental Health, Child Mind Institute (CMI), New York, New York, United States of America; 2 Department of Mental Health, Mozambique Ministry of Health, Maputo, Mozambique; 3 Hospital Psiquiátrico de Nampula, Nampula, Mozambique; 4 Programa de Pós Graduação em Psiquiatria e Ciências do Comportamento, Universidade Federal do Rio Grande do Sul (UFRGS), Porto Alegre, Brazil; 5 Hospital de Clínicas de Porto Alegre (HCPA), Porto Alegre, Brazil; 6 Department of Psychiatry, Universidade de São Paulo (USP), São Paulo, Brazil; 7 Instituto de Psiquiatria, Hospital das Clínicas de São Paulo, São Paulo, Brazil; 8 Medical Council, Centro Universitário de Jaguariúna (UNIFAJ) and Centro Universitário Max Planck (UNIMAX), Jaguariúna and Indaiatuba, São Paulo, Brazil; 9 National Institute of Developmental Psychiatry and National Center for Innovation and Research in Mental Health, São Paulo, Brazil; 10 Department of Medicine, Beira Central Hospital, Beira, Mozambique; 11 Faculty of Health Science, Catholic University of Mozambique, Beira, Mozambique; 12 Department of Psychiatry and Department of Preventive Medicine, Universidade Federal de São Paulo, São Paulo, Brazil; 13 Mozambique Institute for Health Education and Research (MIHER), Maputo, Mozambique; 14 Center for Global Mental Health Equity, University of Michigan, Ann Arbor, Michigan, United States of America; University of Gothenburg: Goteborgs Universitet, SWEDEN

## Abstract

Mozambique is home to 18 million children and adolescents largely underrepresented in mental health research. We conducted a systematic review of evidence-based resources (prevalence estimates, assessment instruments, and interventions) on mental health-related outcomes addressing individuals aged 0–19 years in Mozambique. We searched PubMed, Web of Science, PsycINFO, CINAHL, Google Scholar, African Index Medicus, and local catalogues. Extraction followed COSMIN and Cochrane manuals (PROSPERO registration: CRD420251016208). The search yielded 81 unique publications, including 61 reports presenting prevalence estimates from 25 studies, 29 reports providing data on 42 assessment instruments, and 9 intervention reports describing 8 intervention trials. The clinical prevalence of mental disorders were estimated in two surveys, conducted in Nampula (ADHD: 13·4%; N = 748, 6–18 years) and Maputo (any mental diagnosis: 23%, anxiety disorders: 17·5%; major depression episode: 8·5%; disruptive behavior disorder: 6·6%; substance use disorders: 4·5%; ADHD: 3·3%; PTSD: 1·2%; N = 486, 12–19 years). Most research focused on psychosocial adversities, with nationally representative adolescent surveys reporting significant exposure to violence (physical/emotional violence annually: 24·86%; girls suffering intimate partner violence: 21·11%; lifetime sexual abuse: 16·55%). National suicide rates were highest among 15–19-year-olds (1·53 per 100,000). Only four instruments were cross-culturally adapted and showed reliable psychometrics (MINI-KID, GAD-7, PHQ-A, and SDQ), whilst unadapted tools performed poorly. There was a single randomized clinical trial which reported a cost-effective intervention for neurodevelopment among high-poverty preschoolers. Children and adolescents in Mozambique face significant psychosocial adversity, and available evidence suggests a substantial burden of mental disorders according to limited clinical data. There are few available instruments assessing mental conditions, and culturally sensitive approaches are warranted for strengthening local research.

## Introduction

The emerging field of global mental health requires strengthening resources in lower-middle income countries (LMIC), where approximately 90% of the world’s population under 19 lives but remain underrepresented in research data [1]. A recent global analysis shows the mental health data gap is especially pronounced across sub-Saharan African countries, with a near absence of nationally representative data on children and adolescents [2]. Mozambique, located in Southern Africa, is an illustrative case. The country is home to more than 18 millions of children and adolescents, representing over 55% of the total demographic composition [1]. It is estimated that the majority of these youth live below the poverty line, with socioeconomic vulnerabilities extending to climate-related disasters, exposure to violence and conflict, and the HIV/AIDS epidemic [3]. Their mental health and psychosocial development have gained increased attention from the national government and international agencies, with the Ministry of Health including children and adolescents as a priority in the Strategy and Action Plan for Mental Health 2016–2026 [4].

Mental health resources in Mozambique are critically strained. The country has fewer than 500 specialists (including psychiatrists, psychologists, and psychiatric technicians), with the first child and adolescent psychiatrist being trained in 2021 [5,6]. This shortage is reflected in the limited volume of research produced locally. A 2019 systematic review identified just 130 mental health–related publications from Mozambique, revealing substantial gaps in epidemiological data [7]. Despite these constraints, research output appears to be increasing: a PubMed search using the terms “mental health” AND “Mozambique” yields 189 records, 144 of which have been published since 2020. Some of this work explicitly focuses on bridging gaps in evidence-based resources for children and adolescents, including global and national surveys and work validating assessment instruments [8].

In non-Western contexts, synthesizing and appraising extant research is essential to enhance the reach and availability of evidence-based resources such as prevalence rates, assessment instruments, or intervention programs. National-level compilations on child and adolescent mental health have been conducted in countries such as Greece, Brazil, and South Africa, providing valuable tools to inform practice, policy, and research [9–12]. This study aims to synthesize the scientific literature on child and adolescent mental health in Mozambique. We conducted a systematic review of prevalence estimates, assessment instruments, and interventions addressing mental health–related outcomes in this population. Our goal is to present a comprehensive overview of evidence-based resources available in the country and to take practical insights for public health.

## Materials and methods

We draw on national reviews conducted in Greece, Brazil, and South Africa, with a comprehensive search strategy followed by classification of studies into three independent arms (prevalence, assessment instruments, and interventions) [9–12]. The protocol has been published in PROSPERO [CRD420251016208]. All research conducted in Mozambique requires registration, and we obtained approval from the Ethics Committee of the Faculdade de Medicina da Universidade Eduardo Mondlane (UEM) and the Hospital Central de Maputo [CIBSFM&HCM/42/2025]. This review follows the Preferred Reporting Items for Systematic Reviews and Meta-Analyses (PRISMA) guidelines and the PRISMA-COSMIN manual for reporting systematic reviews of measurement instruments (see checklist in Table A in S1 Appendix) [13,14].

### Inclusion criteria

Our scope was the mental health of children and/or adolescents in Mozambique considering outcomes in a broad sense (e..g: mental disorders, substance use, suicidality, life adversities, childhood maltreatment and neglect, bullying, quality of life, wellness, neurodevelopment, learning difficulties, literacy, stigma). For eligibility, studies had to report specific data for samples in Mozambique with an average age up to 19-years-old (regardless of outliers), including data collected through self- or proxy-reported measures (for instance, caregiver- or teacher-rated scales measuring children’s symptoms). This scope builds upon comprehensive cataloguing efforts in Brazil, Greece, and South Africa [9–11]. It was initially designed to encompass all DSM-5 diagnostic domains and was subsequently expanded to incorporate additional areas identified through mapping of the national literature corpus.

Eligible studies were classified into one or more of three review arms based on the following criteria: (1) prevalence studies included community, school or clinical surveys using structured instruments, questionnaires, or epidemiological registries; (2) assessment instruments included studies that developed, translated or validated tools, further including studies that solely applied an instrument if that was the unique report of the tool in the country; (3) interventions studies with experimental (or quasi-experimental) designs, as well as translation, adaptation, or development of intervention programs.

We included peer-reviewed articles, academic letters, book chapters, theses and dissertations, and institutional reports, without restriction of language or date. For repeated datasets, we included the most recent and/or most comprehensive reports. We excluded multi-country studies that did not provide specific data for Mozambique, as well as general population studies without specific data for children or adolescents. Conference abstracts were not eligible.

### Search strategy and screening

We searched multiple data sources from inception to August 6th 2026: (1) international databases, including PubMed, Web of Science, CINAHL, PsycINFO, and Google Scholar; (2) the African Index Medicus; (3) local university repositories from Universidade Lúrio (UniLúrio) and Universidade Eduardo Mondlane (UEM); (4) publications from the National Health Institute (Instituto Nacional de Saúde – INS); and (5) the local health journals, Revista Moçambicana de Ciência de Saúde (RMCS) and Revista Médica de Moçambique (RMM).

A comprehensive query combined terms associated with children/adolescents, Mozambique, and mental health-related outcomes, being adapted to the syntax and language (English and/or Portuguese) of each database (see Table B in S1 Appendix). First, records were retrieved from PubMed, Web of Science, CINAHL, and PsycINFO. Following deduplication, two independent authors conducted primary screening (based on titles and abstracts) and a single reviewer performed full-text screening. Second, we searched Google Scholar as a complementary source, adapting the query to its crawler-based engine using simplified terms in English and Portuguese. A single author screened results until 100 consecutive entries produced no new relevant studies.

Next, regional and local data sources were also screened by a single author. African Medicus Index was searched for records containing “Mozambique” or “Moçambique”; the UEM repository was searched with Portuguese terms related to children and adolescents; and the full catalogues of the remaining sources (INS, UniLúrio, RMCS, RMM) were manually verified.

The research team responsible for screening and data extraction (LEM, ACC, and JAV) was the same that had conducted these procedures in the national review in Brazil [10]. Prior to primary and secondary screening, a pilot calibration exercise was performed on 10% of the dataset to align inclusion decisions across reviewers. Regular meetings were held throughout screening and extraction to maintain consistency and shared understanding of criteria. When adopting single-reviewer screening procedures, 20% of the records were randomly verified by a second reviewer (LEM); this author also resolved discrepancies arising from two author independent screening. This strategy draws on prior national reviews which utilized a single-reviewer approach emphasizing sensitivity over specificity to maximize article inclusion (i.e., when in doubt, studies were included to make the review as comprehensive as possible) [9–12]. The rationale is fully detailed in the Brazilian systematic review [10]. In brief, this approach is particularly well-suited to compilation reviews and has demonstrated reliable performance in empirical evaluations informing Cochrane guidance [15–17].

The review was managed using Rayyan software. Potential duplicates were detected, automatically removing pairs with a 100% match in title/DOI or Overall Similarity Percentage above 95% (a metric calculated based on all characters and metadata associated with the reference); remaining candidates were manually verified. This threshold was informed by an assessment of the deduplication tool, which suggested that an initial 95% cut-off carries a negligible risk of false exclusions [18].

### Data extraction

Data collection was conducted independently for each review arm, following specific methodological guidelines. A single reviewer performed the initial extraction and quality appraisal, and all datasheets were subsequently verified by LEM.

Prevalence studies followed procedures established in a widely cited systematic review on child and adolescent mental health disorders [19]. Risk of bias is assessed using a validated tool that evaluates external and internal validity, analytical bias, and population representativeness [20]. Table C in S1 Appendix presents extraction fields, with each estimate from each study representing a separate data entry.

For assessment instruments, we followed the Consensus-based Standards for the Selection of Health Measurement Instruments (COSMIN) for data extraction, quality assessment, and psychometric appraisal [21]. Table D in S1 Appendix shows extraction fields, collecting information for each instrument reported in a study on a separate line. Next, data is classified using psychometric criteria outlined in Table E in S1 Appendix.

Intervention studies were extracted following the Cochrane Manual for Systematic Reviews of Interventions [22]. Methodological quality was assessed with the revised Cochrane tool for randomized trials (RoB 2) and the Joanna Briggs Institute (JBI) checklist for non-randomized designs [23,24]. Extraction fields are detailed in Table F in S1 Appendix.

### Data synthesis and presentation

We followed previous national compilation efforts to synthesize all available evidence in an accessible format, aiming at presenting a “catalogue of evidence” that was informative, comprehensive, and able to account for the substantial heterogeneity across populations, study methodologies, and outcomes [9–11].

Data were presented for each major area (prevalence, instruments, and interventions) and organized according to diagnostic domains. These domains included mental health disorders, mental health symptoms, and mental health-related outcomes (such as substance use, psychosocial adversities, and quality of life).

For prevalence, data was presented for each study (aggregating findings across multiple reports and secondary analyses referring to the same parent dataset). To align with the diagnostic domains, studies were initially grouped by methodological design: clinical estimates utilizing diagnostic interviews (reporting mental health disorders), estimates using mental health screening scores (reporting mental health symptoms), and findings from unstructured questionnaires and other surveys (reporting mental health-related outcomes). Within each of these categories, studies were sequentially sorted by sample size.

For assessment instruments, data was presented by each tool. The psychometric classification per instrument was summarized by aggregating data across studies according to the codes presented in Table G in S1 Appendix (with all supporting data detailed in the footnotes).

For interventions, data were presented for each trial (encompassing findings reported across one or multiple publications). Due to the limited number of included studies, all data could be synthesized within a single table. We opted to order the studies by sample size rather than by diagnostic category.

### Ethics statements

We obtained ethical approval from the Scientific Committee and Institutional Bioethics Committee for Health of the Faculty of Medicine at Eduardo Mondlane University and Maputo Central Hospital (Comité Científico e Comité Institucional de Bioética para Saúde da Faculdade de Medicina da Universidade Eduardo Mondlane e Hospital Central de Maputo) [Reference: CIBSFM&HCM/42/2025]. Although this study did not involve direct participation of human subjects, national regulations in Mozambique require local ethical approval for all review studies.

## Results

Fig 1 presents a flowchart with search outcomes (see Fig A in S1 Appendix for PRISMA flowchart; see Table H in S1 Appendix for exclusion reasons; S1 Data provides full access to original search files and documents all screening decisions). Following the screening of 5,760 records, 81 unique publications were eligible for inclusion in one or more areas: 61 reports on prevalence estimates from 25 datasets, 29 reports containing data on 42 assessment instruments, and 9 reports detailing 8 intervention trials. S2 Data contains the full data sheets, which detail all included reports, extracted data, and risk of bias assessments.

**Fig 1:**
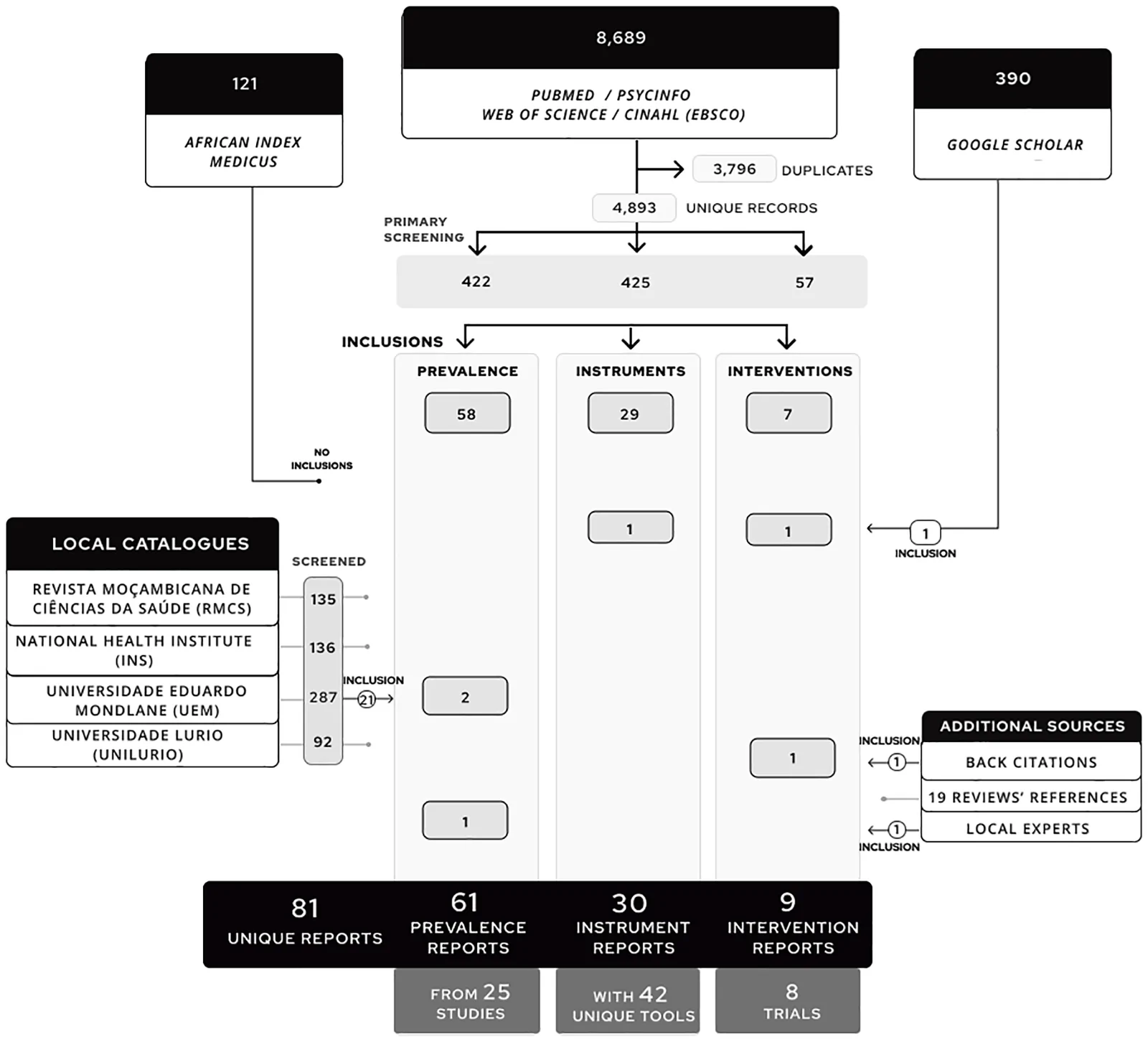
Flowchart of inclusions.

### Overview of scientific production

Fig 2 presents the extent of evidence-based resources, while Fig 3 shows temporal trends of publications. There were 75 peer-reviewed articles, 4 theses/dissertations, and 2 institutional research reports. Research output in Mozambique is relatively recent, with the vast majority of reports published in the past decade. The evidence base is skewed toward prevalence estimates, featuring very few intervention trials. Childhood adversity and trauma constitute a prominent line of inquiry, accounting for 28 of the included reports. Prevalence reports are also centered on substance use, and assessment instruments have a substantial concentration on neurodevelopment tests.

**Fig 2:**
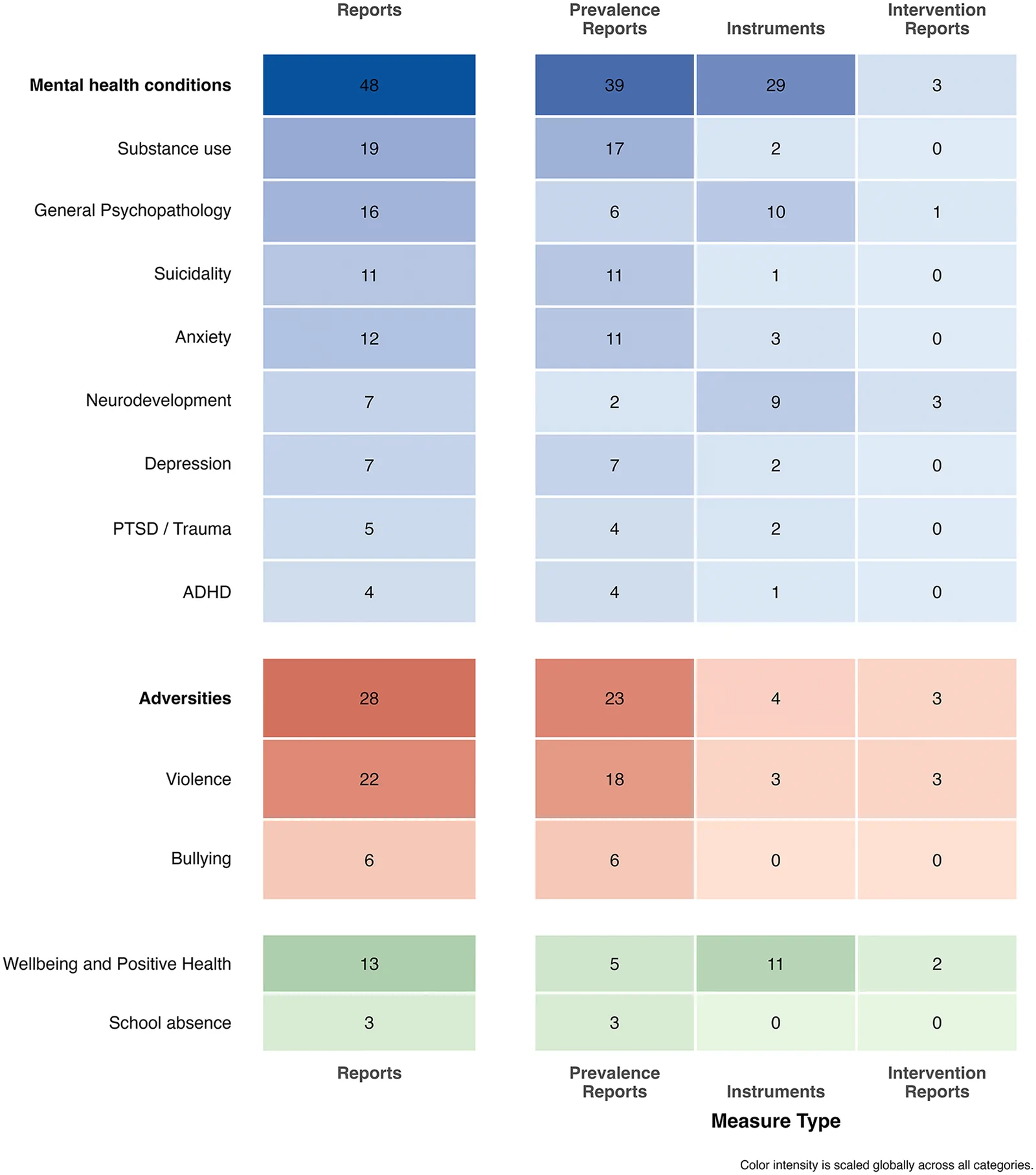
Availability of evidence-based resources per domain of inquiry.

**Fig 3:**
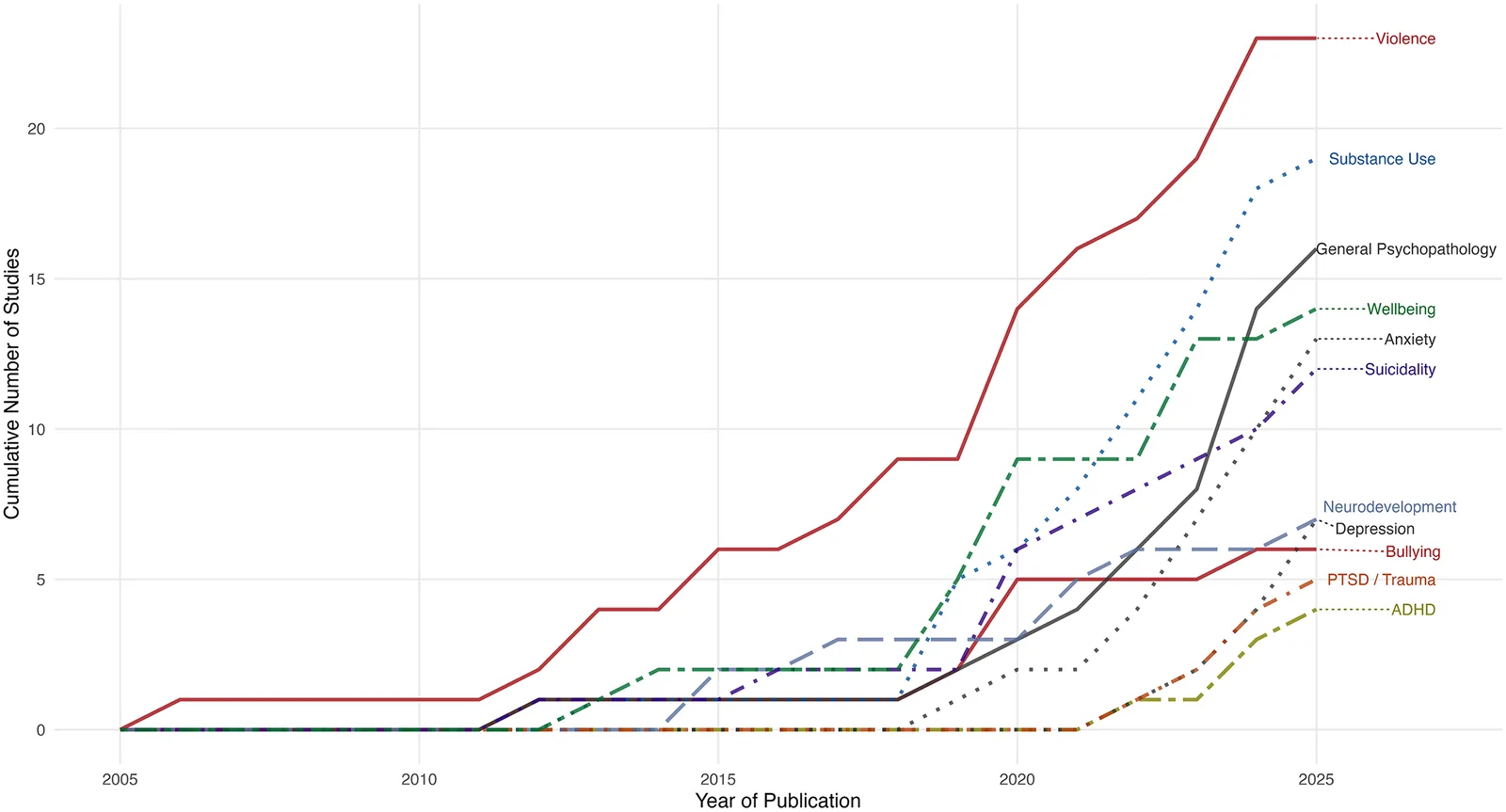
Cumulative trend of publications per domain of inquiry.

International authorship is predominant [25]. Among the 31 articles with a Mozambican affiliation, just 18 credited a local institution in first authorship and only 6 in senior authorship. Two PhD theses were associated with international universities (Arizona State University and Universidade do Porto) and two master dissertations were produced in a local postgraduate program (UEM) [26–29]. Institutional research reports were published by the World Bank and ActionAid [30,31].

International funding is also the primary source of support. There were several multinational surveys run by organizations such as the WHO [32–53], as well as national surveys sponsored by agencies like UNICEF [54] and the NIMH [55]. International development programs promoted 7 out of 8 intervention studies. Although more limited, national funding involves various public bodies. The National Demographic and Health Survey is a representative study which is implemented by the National Institute of Statistics, the Ministry of Health, and the National Institute for Health [56]. Other contributing agencies include the National Council for Combating AIDS (CNCS) and the National Research Fund under the Ministry of Science, Technology, and Higher Education [26].

### Prevalence of mental health disorders

Table 1 presents estimates on the prevalence of mental health disorders. There were only 3 studies including clinical data according to manualized criteria. A study in Maputo recruited 486 adolescents, leading to three reports estimating the prevalence of any mental diagnosis (23%), any anxiety disorders (17·5%), major depression episode (8·5%), disruptive behavior disorder (6·6%), substance use disorders (4·5%), ADHD (3·3%), and PTSD (1·2%) [8,57,58]. Another survey in Nampula included 748 children and adolescents, with two reports on the prevalence of ADHD at 13·4% [59,60]. Additionally, a non-probabilistic study included 110 individuals aged 1–20, using clinical batteries to estimate the prevalence of neurodevelopmental impairment (mild: 18·2%; moderate-to-severe: 0·9%) [61].

**Table 1:** Prevalence estimates on mental health disorders.

Prevalence estimates based on structured clinical interview and manualized criteria						
Study	Sample recruitment	Sample (N) Description	Location Year	Diagnostic criteria / clinical interview	Findings	RoB
Daniel et al. (2024) [59] [60],	School-based, probabilistic sample Two-step sample screening: 1. Screening survey with SNAP-IV 2. Clinical interview for high-risk	N = 748 6 to 18 years-old 54·6% female	Nampula 2022 - 2023	**DSM-5 criteria** Kiddie Schedule for Affective Disorders and Schizophrenia for School-aged Children (K-SADS-PL)	Current prevalence ADHD: 13·4% (95% CI 11·5%-19·2%) SNAP-IV positive screening (teacher- or caregiver-rate): 10·16%	Low
Come et al. (2022) [57] Lovero et al. (2022) [8] King et al. (2026) [58]	School-based, probabilistic sample Single-step sampling: all participants completed screening scores and diagnostic interviews	N = 486 12 - 19 years-old 66·2% female	Maputo 2019	**DSM-4 and ICD-10 criteria** Miniature International Neuropsychiatric Interview for Children and Adolescents (MINI-KID)	Current prevalence: Any MINI Diagnosis: 23% Anxiety disorders:* 17·5% Major Depressive Disorder Episode: 8·5% Disruptive Behavior Disorder:** 6·6%ADHD: 3·3% Alcohol Use Disorder: **2·7%** Drug Use Disorder: **2·1%** **PTSD: 1·2%** Suicidal thoughts (past month): 16·42% Suicidal behavior (past month): 6·16% Suicide attempt (lifetime): 8·89%	Low
Paul et al. (2022) [61]	Case-control on a cohort of survivors of invasive Group B Streptococcus infant Controls identified via Health and Demographic Surveillance System	N = 110 1 - 20 years-old 54·5% female Control group matched for survivors of invasive bacterial infection during early infancy	Maputo 2019 - 2021	WHO and Global Burden of Disease (GBD) criteria for neurodevelopmental impairment Battery of neurodevelopmental tests: Malawi Developmental Assessment Tool (MDAT) Cambridge Neuropsychological Test Automated Battery (CANTAT)	Neurodevelopmental impairment (moderate to severe): 0·9% Neurodevelopmental impairment (mild): 18·2%Cognitive impairment (moderate to severe): 4·8% Cognitive impairment (mild): 14·4% *No clinical cases were identified through the Child Behavior Checklist (CBCL), which was attributed to a poor cultural fit of the instrument*	High
**Prevalence estimates based on screening instruments**						
Study	Sample recruitment	Sample (N) Description	Location Year	Assessment tools / cut-off scores	Findings	
2022-2023 Mozambique Demographic and Health Survey [56] [62],	National Probabilistic Community-based	N = 4548 15 - 19 years-old 68·35% female	Nationwide 2022 -2023	Generalized Anxiety Disorder- 7 item (GAD-7) ≥ 5 & ≥ 7 Patient Health Questionnaire – 9 item (PHQ-9) ≥ 8 & ≥ 10	**Positive screening for:** Generalized Anxiety Disorder (2 weeks): 6·3% - 18·51% Major Depressive Disorder (2 weeks): 6·6% - 10·25%	Low
Di Gennaro et al. (2022) [54]	Regional Clinical sample of youth accessing health services	N = 2018 *(HIV + : 1096 / HIV-: 2012* 63% female 10 - 24 years-old	Sofala Province 2019 - 2021	**Cut-off scores:** CAGE questionnaire ≥ 2 Generalized Anxiety Disorder- 7 item (GAD-7) ≥ 10 Patient Health Questionnaire – 9 item (PHQ-9) ≥ 11 Post-Traumatic Stress Disorder – 5 item (PTSD-5) ≥ 3	**Positive screening (HIV+ group)**Alcohol use disorder: 15·1% PTSD / Trauma: 14·7% Anxiety: 10·3% Depression: 7·1% **Positive screening (HIV- group)** Alcohol use disorder: 10% PTSD / Trauma: 9·4% Anxiety: 7·6% Depression: 7·6%	High
Chimbutane et al. (2023) [63]	Regional Probabilistic School-based	N = 1383 60% female 13 - 16 years-old	Rural areas in Nampula province (Nacaroa, Muecete, and Murrupula) 2021	Generalized Anxiety Disorder- 7 item (GAD-7) > 10WHO-5 Wellbeing < 50	Anxiety symptoms (moderate to severe): 10% Low well-being: 31%	High
Disrupting harm survey [64]	Multi-country survey Probabilistic Online	N = 999 45% female 12 - 17tears	Nationwide 2020-2021	Center for Epidemiologic Studies Depression Scale (CES-D) Paykel Suicide Scale UNICEF’s Youth Empowerment Project scale *Prevalence was based on response to single items instead of cut-offs*	Depression: 25.3% Self-harm: 11.1% Suicidal ideation/attempt: 27.2%	High
Igreja et al. (2024) [25]	Regional Non-probabilistic Convenience sample in households and schools	N = 794 9 to 21 year-old 45·8% female	Gorongosa District in Sofala Province 2013	Beck Depressive Inventory-II (BDI-II) Self-Report Questionnaire (SRQ-20)Nocturnal Intrusions after Traumatic Experiences (NITE)	**Depressive symptoms** Mild: 22·2% Moderate: 17·8% Severe: 14·2% **Anxiety symptoms** (high): 39·3% **Sleep disturbances and anxiety dreams** (severe): 37·3%	High
Manafe et al· (2024) [65]	Regional Non-probabilistic Convenience community-based sample	N = 750 14 to 20 years-old 66·2% female	Cabo Delgado 2023	Generalized Anxiety Disorder- 7 item (GAD-7)>= 10Primary Care Post-Traumatic Stress Disorder Checklist (PC-PTSD-5)>= 4Patient Health Questionnaire – 9 item (PHQ-9)>= 10	PTSD symptoms: 14·6% Depressive symptoms: 9·6% Anxiety symptoms: 8%	High
*Langa et al· (2022)* [66]	*Regional* *Probabilistic* *Community-based sample (household)*	*N = 2772–14 years-old*	*Mocuba district, Zambezia province* *2018*	*Clinical question on whether the participant presented inappropriate behavior, had auditory hallucination or delusions*	*Psychotic symptoms: 10·5% (not based on a valid tool)*	*High*
CombinADO survey [55]	Regional Non-probabilistic Clinical sample of adolescents living with HIV	N = 213 15 - 18 years-old 74,87% female	Nampulo 2019	Generalized Anxiety Disorder- 7 item (GAD-7) > 10Patient Health Questionnaire – 9 item (PHQ-9) > 10	Anxiety symptoms: 12·2%Depression symptoms: 11·7%	High
Thumbo (2025) [29]	Regional Non-probabilistic Clinical sample from a service for vulnerable adolescents	N = 45 14 - 17 tears 48,9% female	Zimpeto 2025	Generalized Anxiety Disorder- 7 item (PHQ-9) Generalized Anxiety Disorder- 7 item (GAD-7)	Anxiety symptoms (mild): 71% Anxiety symptoms (moderate): 28,9% Depressive symptoms (mild): 73,3% Depressive symptoms (moderate): 26,7%	High

**Notes:** *Anxiety Disorders groups Generalized Anxiety Disorder, Separation Anxiety, Social Phobia, Panic Disorder, and Agoraphobia. **Disruptive Behavior Disorder groups Conduct and Oppositional Defiant Disorder

National data points on mental health disorders were limited to screening cut-off scores reported at the 2022–2023 Mozambique Demographic and Health Survey [56] [62],. This study involved 4548 participants aged 15–19 years-old completing the Generalized Anxiety Disorder 7-item (GAD-7) and the Patient Health Questionnaire 9-item (PHQ-9). It was estimated that 6·3% to 18·51% presented significant symptoms of generalized anxiety disorders (GAD-7 ≥ 5 or ≥ 7), whilst 6,6% to 10,25% experienced significant major depressive disorder symptoms (PHQ-9 ≥ 8 or ≥ 10). Although the Disrupting Harm project utilized a nationally representative survey of Mozambican adolescents incorporating structured scales, prevalence estimates relied on single-item responses rather than validated cut-off scores [67].

At regional level, a study involved a clinical sample of 2018 adolescents and young adults attending health services in Sofala found that 1096 HIV+ participants presented higher symptoms of mental disorders: 15·1% had positive screening for alcohol use disorder (CAGE ≥ 2); 14.7% for PTSD (PTSD-5  ≥ 3); 10,3% for anxiety disorders (GAD-7 ≥ 10), and 7·1% for major depressive disorder (PHQ-9 ≥ 1) [54]. Another study conducted in rural Nampula with a community based sample of 1,383 adolescents aged 13–16 years estimated that 10% presented moderate to severe (GAD-7 ≥ 10) [63]. Additional estimates came from convenience samples [25,29,65].

### Prevalence of mental health-related outcomes

Table 2 provides findings from 17 surveys reporting on the use of substance, exposure to adversities, and psychosocial symptoms. Table I in S1 Appendix summarizes an additional 8 register-based articles describing clinical outcomes.

**Table 2:** Prevalence surveys on mental-health related outcomes.

Study	Population	Sample	Assessment	Use of substance	Exposure to adversities	Other psychosocial symptoms
GYTS - Global Youth Tobacco Survey [68]	National School-based 2013-2018	N = 559913–17 years-old	Questionnaire items	Current smokers of cigarette: 11·7% Current use of smoke tobacco product other than cigarette: 4·2% Current use of smokeless tobacco: 4·3% Current use of any tobacco product: 20·7%		
2022-2023 Mozambique Demographic and Health Survey [56] [62],	National Community-based 2022-2023	N = 4548 15 - 19 years-old 68·35% female	Generalized Anxiety Disorder- 7 item (GAD-7) ≥ 5 or GAD-7 ≥ 7 Patient Health Questionnaire – 9 item (PHQ-9) ≥ 8 or PHQ-9 ≥ 10 Items from questionnaire	Current alcohol use (1 month): 6·33% Tobacco use (current, last month): 0·9%		Suicidal attempt, ideation, or plan (12 months): 3·65%
2015 Mozambique Global School-based Student Health Surveys (GSHS) [32–53]	National School-based 2015	N = 1918 13 - 17 years-old 48·9% female	Questionnaire items	**Substance use** Alcohol use (current): 13·7% Drunkenness (lifetime): 10% Alcohol use problems (lifetime): 3·2% Tobacco use (current): 2·8%	**Exposure to violence** (last year) Bullying (total): 45·4% Bullying (1–2 days/month): 31·1% Bullying (3–30 days/month): 13·4%Struck/hit by person: 3·6%	Social isolation: 37·6% Psychosocial distress: 21·2%Suicidal ideation:17·7%Anxiety: 10·3%Loneliness: 9·8%
2011 Mozambique Demographic and Health Survey [69]	National Community-based 2011	N = 1483 All female 15 - 19 years-old	Questionnaire items		Physical partner violence: 21·11% Sexual partner violence: 6·34% Physical or sexual partner violence: 23·08%	
2019 Violence Against Children and Youth Survey (VACS) [70]	National Community-based 2019	N = 1275 13 - 19 years-old	Questionnaire items	Recent heavy alcohol use (15–19 years-old): 4·8% Early alcohol use (13–14 years-old): 1·4%	History of pregnancy: 31·13% Out of school (current): 29% Orphanhood: 26·27% Physical or emotional violence (last year): 24·86%Sexual violence (lifetime): 16·55% Transactional sex (lifetime): 3·76%	
2017 Global Youth Tobacco Survey (GYTS) [47]	National School-based 2017	N = 1211	Questionnaire items	Tobacco use (current): 2·22%		
Disrupting harm survey [64]	Multi-country survey Probabilistic Online	N = 999 45% female 12 - 17 years	Questionnaire items			Self-harm: 11% Loneliness: 35.3%
Family Migration and Early Life Outcomes (FAMELO) [71]	Unclear regions Community-based 2017–2018	N = 651 11 - 17 years-old	Questionnaire items		Ever spank, slap, hit or beat the child (caregiver-rated): 44%	
Mozambique child soldier life outcome study [72]	National Former child soldiers in rehabilitation 1989	N = 504 6 to 15 years-old 50% female	Questionnaire items		Abducted from the family: 64%Experienced physical abuse: 51%Witnessed murder: 77%	
Carneiro et al. (2019) [73]	Maputo Convenience sample in Portuguese private schools 2017	N = 4383–13 years-old51% female	Children’s Sleep Habits Questionnaire (cutoff: 52)			Sleep problems: 28·4%
International Study of Pro/Antisocial Behavior in Young Adults [74] [75],	National Convenience sample 2018–2019	N = 400 18 - 20 years-old 55·6% female	Psychosocial and Family Vulnerability Questionnaire Adverse Childhood ExperiencesQuestionnaire (ACE Questionnaire)	Last year: • Drunkenness: 34·78% • Drug use: 13·4%	Experienced in the first 18 years of life: • Child maltreatment: 70%Physical neglect: 41·82% • Sexual abuse: 36·16% • Domestic violence: 35·22%Emotional neglect: 22·01%Physical abuse: 13·94% • Emotional abuse: 13·21%	
Maguele et al. (2020) [76]	Maputo School-based 2019	N = 368 15 - 19 years-old All female	Questionnaire adapted from the WHO Multi-country Survey of Women’s Health and Domestic Violence against Women		Intimate partner violence (last year): • Any: 65% • Physical: 18·5% • Sexual: 22·3% • Psychological: 57·6% Intimate partner violence (lifetime): • Any: 57·9% • Physical: 20% • Sexual: 25% • Psychological: 53%	
Stop Violence Against Girls in School [77]	Rural areas in Maputo Convenience-sample 2013 and 2019	N = 3638–17 years-old All female	Questionnaire items		2009: • Any sexual violence: 29·3% • Peeping: 18·5% • Touching: 16·2% • Forced sex: 8·8% • Sex for goods: 1·8% 2013: • Any sexual violence: 39·4% • Touching: 29·2% • Peeping: 23·7% • Forced sex: 8·8% • Sex for goods: 0%	
Ribeiro et al. (2020) [78]	Maputo School-based 2010	N = 208 12 - 18 years-old 65·3% female	Questionnaire items	Any alcohol use (lifetime): 55% Any alcohol use (last month): 22% Drunkenness (lifetime): 18%Drunkenness (last month): 8%Tobacco (lifetime):9%Tobacco (last month): 4%		
Gouveia et al. (2023) [79]	Maputo Case-control study during crisis intervention in a school with cases of Emotional Contagion Behavior (ECB), including girls referred to treatment and convenience-sampled controls	N = 154 (57 cases, 97 controls) 16 - 24 years-old All female	Questionnaire items	**Alcohol use (past two week)** Case: 5·3% Control: 7·2%		
Libombo et al. (2012) [80]	Case-control study with community-based convenience sample of orphans and non-orphans in a district of Tete	N = 150 (76 orphans and 74 non-orphans) 10 - 14 years-old	Questionnaire items		**Orphans**Rarely have time to play: 14% No trusted adult at home:35% Bullying: 58% Hunger at bedtime for two or more days in the last week: 57% Undeserved punishment 43% Child labor (work outside home) 61% Child labor (work outside home 6–7 days week 16% Currently not at school 20% **Non-orphans**Rarely have time to play 4% No trusted adult at home 7% Bullying 32% Hunger at bedtime for two or more days in the last week 26% Undeserved punishment 45% Child labor (work outside home) 58% Child labor (work outside home 6–7 days week 3% Currently not at school 5%	**Orphans** Suicidal ideation 37% Internalizing Disorders 6,1% **Non-orphans** Suicidal ideation 8% Internalizing Disorders 0,66%
Chaquisse et al. (2018) [81]	Nampula Pregnant women accessing antenatal care 2013 - 2014	N = 148 < 18 years-old All female	Conflict Tactics Scale 2		Physical abuse (lifetime): 34,5%Physical abuse (past year): 33,8%Sexual abuse (lifetime): 44,6%Sexual abuse (past year): 43,9%	

At national level, there were 7 probabilistic surveys with adolescents, with sample sizes ranging from 999 to 5,599 participants [32–53,56,62,67–70]. These provided several data points on substance use (e.g.,: current tobacco use: 20·7%; current alcohol use: 13·7%), psychosocial symptoms (e.g.,: social isolation: 37·6%; suicidal ideation: 17·7%), socioeconomic vulnerabilities (e.g.,: out of school: 29%; orphanhood: 26·27%), and exposure to violence (e.g.,: physical/emotional - last year: 24·86%; intimate partner violence suffered by girls - last year: 23·08%; lifetime sexual violence: 16·55%; frequent bullying - past month: 13·4%).

Health registers provided further nationwide data points: a suicide rate of 0·65 per 100,000 people aged 5–19 was estimated based on a study collecting data from 2014 to 2019, with the highest rate of 1·53 among adolescents aged 15–19 [28]. All violent deaths are expected to be referred to forensic facilities, with three referral services operating in Mozambique at the time of the study. However, only absolute numbers were reported (70 suicides).

### Instruments

Table 3 shows cross-cultural adaptation and psychometric properties of 22 tools, including 2 diagnostic interviews, 12 screening scales, and 6 neurodevelopment tests. Table J in S1 Appendix further lists 20 tools applied without formal adaptation or validation.

**Table 3:** Instruments adapted or validated in Mozambique.

Instrument	Population	Setting	Rater	Language		Psychometrics									REFERENCES
DIAGNOSTIC INTERVIEWS FOR MENTAL DISORDERS				**Cultural adaptation**	**Other dialects**	**CC**	**CR**	**CV**	**IC**	**IR**	**ME**	**R**	**SV**	**TR**	**References**
Mini-International Neuropsychiatric Interview for Children and Adolescents (MINI-KID)	Children Adolescents	School	Health Professional	Valid	NA	NA	NA	**+** ^a^	NA	NA	NA	NA	NA	NA	[8][57],
Kiddie Schedule for Affective Disorders and Schizophrenia for School-aged Children (KSADS-PL) - ADHD and Disruptive Behavior Disorders modules	Children Adolescents	School	Health Professional	Unadapted Brazilian version	NA	**?** ^b^	NA	NA	NA	NA	NA	NA		NA	[59][60],
SCREENING INSTRUMENTS FOR MENTAL DISORDERS				**Cultural adaptation**	**Other dialects**	**CC**	**CR**	**CV**	**IC**	**IR**	**ME**	**R**	**SV**	**TR**	**References**
General Anxiety Disorder (GAD-7)	Adolescents	School	Self-report	Valid	NA	NA	**+** ^c^	**+** ^d^	**+** ^e^	NA	NA	NA	NA	**+** ^f^	[8,55,63]
Patient Health Questionnaire-Adolescent (PHQ-A)	Adolescents	Clinical School	Self-report	Valid	NA	NA	**+** ^g^	NA	**+** ^h^	NA	NA	NA	NA	**+** ^i^	[8][55],
Strengths and Difficulties Questionnaire (SDQ)	Adolescents	Clinical	Self-report	Valid	NA	NA	**+** ^j^	NA	**+** ^k^	NA	NA	NA	NA	**+** ^l^	[8][57],
Children’s Sleep Habits Questionnaire (CSHQ)	Children Adolescents	School	Caregiver	Unadapted Portuguese version	NA	NA	**?** ^m^	NA	**+** ^n^	NA	NA	NA	NA	NA	[73]
Swanson, Nolan, and Pelham scale (SNAP-IV)	Children Adolescents	School	Caregiver Teacher	Unadapted Brazilian version	NA	**?** ^o^	**-** ^p^	**-** ^q^	NA	NA	NA	NA	NA	NA	[59,60]
Child Behavior Checklist (CBCL)	Children Adolescents	Community	TeacherHealth professional	Unadapted version	NA	**-** ^r^	NA	NA	NA	NA	NA	NA	NA	NA	[59,61,82]
Beck Depression Inventory (BDI-II)	Children Adolescents	Community School	Self-report	Valid	**Chi-Gorongose**	NA	NA	NA	**+** ^s^	NA	NA	NA	NA	NA	[25]
Nocturnal Intrusions after Traumatic Experiences Questionnaire (NITE)	Children Adolescents	Community School	Self-report	Valid*	**Chi-Gorongose**	NA	NA	NA	**+** ^t^	NA	NA	NA	NA	NA	[25]
Self Report Questionnaire (SRQ-20)	Children Adolescents	Community School	Self-report	Valid*	**Chi-Gorongose**	NA	NA	NA	**+** ^u^	NA	NA	NA	NA	NA	[25]
Depression Anxiety and Stress Scale-21	Adolescents	Community	Self-report	Valid	NA	NA	NA	NA	NA	NA	NA	NA	NA	NA	[83]
Youth Psychopathic Traits Inventory - Short Version	Adolescents	Community	Self-report	Valid	NA	NA	NA	NA	NA	NA	NA	NA	NA	NA	[83]
Deviant Behavior Variety Scale	Adolescents	Community	Self-report	Valid	NA	NA	NA	NA	NA	NA	NA	NA	NA	NA	[83]
NEURODEVELOPMENTAL ASSESSMENT				**Cultural adaptation**	**Other dialects**	**CC**	**CR**	**CV**	**IC**	**IR**	**ME**	**R**	**SV**	**TR**	**References**
MacArthur-Bates Communicative Development Inventories (MBCDI) Short Form	Children Adolescents	Community	Caregiver	Valid	None	NA	NA	NA	NA	NA	NA	NA	NA	NA	[84][85],
Ages & Stages Questionnaire (ASQ)	Children	Community	Caregiver	Valid	**Changana**	NA	NA	NA	NA	NA	NA	NA	NA	NA	[31][86],
Peabody Picture Vocabulary Test (PPVT)	Children	Community	Self-report	Valid	**Changana**	NA	NA	NA	NA	NA	NA	NA	NA	NA	[31]
Early Development Instrument (EDI)	Children	Community	Teacher	Valid	NA	NA	NA	NA	NA	NA	NA	NA	NA	NA	[31]
Cambridge Neuropsychological Test Automated Battery (CANTAB)	Children	Clinical Community	Health professional	Unclear	NA	NA	NA	NA	NA	NA	NA	NA	NA	NA	[61]
Malawi Developmental Assessment Tool (DMAT)	Children	Clinical Community	Health professional	Unclear	NA	NA	NA	NA	NA	NA	NA	NA	NA	NA	[61]
WELLBEING, POSITIVE HEALTH, AND LIFE OUTCOMES				**Cultural adaptation**	**Other dialects**	**CC**	**CR**	**CV**	**IC**	**IR**	**ME**	**R**	**SV**	**TR**	**References**
Family Migration and Early Life Outcomes (FAMELO) Questionnaire	Children Adolescents	Community	Caregiver	Developed locally	NA	NA	NA	NA	**+** ^v^	NA	NA	NA	**-** ^w^	NA	[27]
Rosenberg Self-Esteem Scale (RSES)	Adolescents	School	Self-report	Valid	NA	NA	NA	**+** ^x^	**+** ^y^	NA	NA	NA	**+** ^z^	NA	[87]
The World Health Organisation - Well-Being Index (WHO-5)	Children Adolescents	School	Self-report	Unadapted Portuguese version	NA	NA	NA	**+** ^aa^	**+** ^bb^	NA	NA	NA	NA	NA	[63]
Norwegian International Child Development Programme (ICDP) Evaluation Abridged Version	[Children	Community	Caregiver	Valid	**Changana**	NA	NA	NA	NA	NA	NA	**+** ^cc^	NA	NA	[31]
Adverse Childhood Experiences (ACE) Questionnaire	Adolescents	Community	Self-report	Valid	NA	NA	NA	NA	NA	NA	NA	NA	NA	NA	[83]
Brief COPE	Adolescents	Community	Self-report	Valid	NA	NA	NA	NA	NA	NA	NA	NA	NA	NA	[83]
The Altruistic Attitudes Scale	Adolescents	Community	Self-report	Valid	NA	NA	NA	NA	NA	NA	NA	NA	NA	NA	[83]

**Abbreviations**: cross-cultural validity (CC), criterion validity (CR), construct validity (CV), internal consistency (IC), interrater reliability (IR), measurement error (ME), post-traumatic stress disorder (PTSD), responsiveness (R), structural validity (SV), test-retest reliability (TR). “+” stands for a property with positive evidence for validation, “+/-” for mixed evidence, “-” for negative evidence, “?” for available evidence that cannot be classified as positive or negative, “NA” for non-available evidence (see Table B in S1 Appendix).

**Notes: [MINI-KID]** ^**a**^Correlations with SDQ (Area Under the Curve: 0·7 - 0·9; Sensitivity: 0·72 - 0·83; Specificity: 0·67 - 0·74), with GAD-7 (AUC 0·85; Sensitivity 0·78; Specificity 0·72) and with PHQ-A (AUC: 0·84; Sensitivity: 0·81; Specificity: 0·72); **[KSADS-PL]** ^**b**^There is no multigroup factorial analysis as per COSMIN criteria for cross-cultural validity, but authors debate a predictive performance and comorbidity pattern similar to the one found in studies worldwide; **[GAD-7]** ^**c**^Correlation Mini-KID (AUC: 0·85; Sensitivity: 0·78; Specificity: 0·72); ^**d**^Correlation index > 0·83 with WHO-5 well-being; ^**e**^Cronbach Alpha: 0·69 - 0·84; ^**f**^Intra-Class Correlation (ICC): 0·7 (one-week retest); **[PHQ-A]** ^**g**^Correlation with Mini-KID (AUC: 0·84; Sensitivity: 0·81; Specificity: 0·72); ^**h**^Cronbach Alpha: 0·8; ^**i**^ICC: 0·74 (one-week retest); **[SDQ]** ^**j**^Correlation with MINI-KID (AUC: 0·7 - 0·9; Sensitivity: 0·72 - 0·83; Specificity: 0·67 - 0·74); ^**k**^Cronbach Alpha: 0·73; ^**l**^ICC: 0·77 (one-week retest); **[CSHQ]** ^**m**^Correlation with Parent`s Report Form (AUC: not reported; Sensitivity: 0·89; Specificity: 0·71); ^**n**^Cronbach Alpha: 0·75; **[SNAP-IV]** ^**o**^Authors reported individual items were distributed differently than in other countries, as well as poor overall psychometric performance; ^**p**^Correlations with KSADS-PL clinical interview following DSM-5 criteria (AUCs  <  0·53); ^**q**^Correlations between individual symptoms rated by teachers and parents: kappa (k) scores < 0·35; **[CBCL]** ^**r**^The scale was also applied in Argentina, India, Kenya, and South Africa, but only in Mozambique it was unable to detect clinical cases, which authors attributed to poor cultural fit; **[BDI-II]** ^**s**^Cronbach alpha: 0·97; **[NITE]** ^**t**^Cronbach alpha: 0·94; **[SRQ-20]** ^**u**^Cronbach alpha: 0·93; **[FAMELO]** ^**v**^Cronbach Alpha: 0·74 to 0·93; ^**w**^RMSEA: 0·06 and CFI: 0·92; **[RSES]** ^**x**^ΔR2: 0·39 (between self-esteem and a model involving relatedness, competence, and autonomy); ^**y**^Cronbach Alpha: 0·73; ^**z**^Confirmatory Factor Analysis (CFA) for 2-factor model (CFI: 1; RMSEA: 0·000; TLI: 1; SRMR: 0·011); **[WHO-5]** ^**aa**^Correlation with GAD-7 (0·83); ^**bb**^Cronbach Alpha: 0·79; **[ICDP]** ^**cc**^Statistically significant differences between pre- and post-intervention assessment.

The Mini-International Neuropsychiatric Interview for Children and Adolescents (MINI-KID) was the only diagnostic interview that underwent cross-cultural adaptation, demonstrating positive evidence of construct validity according to correlation scores with SQD (Area Under the Curve: 0·7 - 0·9; Sensitivity: 0·72 - 0·83; Specificity: 0·67 - 0·74), GAD-7 (AUC 0·85; Sensitivity 0·78; Specificity 0·72) and PHQ-A (AUC: 0·84; Sensitivity: 0·81; Specificity: 0·72) [8,57].

There were nine cross-culturally adapted screening tools for mental health conditions, with psychometric assessment reported for six of them. The GAD-7, the PHQ-A, and the SDQ showed most favorable psychometric performance, with positive evidence for internal consistency (Cronbach’s alpha > 0·7), test-retest reliability (ICC ≥ 0·7 at one-week retest), and criterion validity (as per correlations with gold-standard MINI-KID) [8,55,63]. The other three tools were adapted to the local dialect *Chi-Gorongose* (Beck Depression Inventory - BDI-II; Self Report Questionnaire - SRQ-20; Nocturnal Intrusions after Traumatic Experiences Questionnaire - NITE), yet psychometric assessment was restricted to internal consistency [25].

Five instruments were used in non-adapted versions originally developed for Brazil or Portugal, which was associated with poor psychometric performance: The Child Behavior Checklist (CBCL) failed to detect clinical problems in the Mozambican sample, despite performing adequately in other national arms of the same study conducted in Argentina, India, Kenya, and South Africa [61,82]. The Swanson, Nolan, and Pelham scale (SNAP-IV) showed low predictive accuracy for ADHD diagnosis [59,60].

All neurodevelopment tests were cross-culturally adapted but lacked psychometric evaluation, with two tools translated to the local dialectic Changana (Ages & Stages Questionnaire - ASQ; Peabody Picture Vocabulary Test - PPVT) [31].

### Intervention studies

Table 4 presents eight intervention studies, including one RCT, four controlled trials, and three uncontrolled pre-post designs. Reports indicated a high risk of bias across all study designs. Programs consisted of 4 psychosocial interventions targeting neurodevelopment, 2 focusing on gender equality, and 1 on mental health promotion.

**Table 4:** Interventions.

Intervention	Target	Design	Setting / Sample	Sample size Age	Results	RoB*	Funding
Preschool program following a structured daily routine with play and learning activities [31]	Neurodevelopment	RCT	School Children from high-poverty rural areas	N = 1907 3 -5 years	Significant gains in developmental skills (cognitive, communication, motor, and socio-emotional) at a cost of $3 per child per month *(no measure of clinical effects)*	**High** Cluster allocation; post-randomization reassignment of groups; differences in control group; voluntary participant; only 41% of eligible children enrolled; unblinded	World Bank Group - Education Global Practice Group
Community-based program involving nutrition, sanitation, and hygiene (part of Transform Nutrition) [86]	Neurodevelopment	Quasi-experimental	Community Children from high-poverty areas enrolled in Transform Nutrition program	N = 961 children 0-23 months	No impact on developmental scores or maternal mental health	**High** Control group was not comparable; lack of valid measurement; unblinded assessment	USAID Advancing Nutrition Program
Adult–child communication (ACC) program developed by Go Girls! Initiative (GGI) with 11 sessions for caregivers [88]	Girl’s vulnerability to HIV	Controlled	Community Adolescent girls in Zambezia and Nampula	N = 603 *(555 caregivers)* 11 - 18 years	Girls reported improvement in relationship with parents, but hard outcomes were not assessed	**High** No baseline assessment; lack of valid instrument; families participating in the intervention might be more prone to benefits; no distal outcome assessing direct effects	President’s Emergency Plan for AIDS Relief (PEPFAR) US Agency for International Development (USAID)
School personnel training with 4-day workshops to promote gender-equitable teaching and safer environments for girls [89]	Girl’s vulnerability to HIV	Controlled	School Teachers working with adolescent girls in Zambezia and Nampula	N = 525 *(67 teachers)* 11 - 18 years	Distal outcomes not assessed, no significant differences in students perception of teachers’ gender bias attitudes nor perceived safety at school	**High** No baseline assessment; lack of valid instrument; indirect intervention delivered with teachers to benefit girls; no distal outcome assessing direct effects	President’s Emergency Plan for AIDS Relief (PEPFAR) US Agency for International Development (USAID)
Stop Violence Against Girls in School participatory program with multi-modal community activities [30] [77],	Violence against girls	Uncontrolled	Community Girls in a rural area in Maputo	N = 363 8 - 7 years	Increased percentage of girls reporting violence and showing gender-equitable attitudes Increased the percentage of teachers with knowledge of laws, policies, and available support. No test of statistical significance.	**High** No control group; lack of valid tool; no distal outcome assessing direct effects; convenience sample of girls participating in the project	An ActionAid’s program supported by the UK’s Big Lottery Fund
International Child Development Programme (ICDP) with 10–12 weekly group sessions on parenting skills for caregivers [90]	Neurodevelopment	Controlled¹	Community Caregivers participating in the ICDP in Maputo	N = 137² 4·4 years *(SD 2·2)*	Statistically significant decrease on child conduct problems scores and improvements on parents’ self-efficacy *(no measures of effect size)* Increased frequency of positive parenting practices	**High** No baseline assessment; differences in control and intervention group; unblinded assessment; lack direct distal outcomes	Part of the International Child Development Programme (ICDP), supported by multiple sources
Train community health workers to implement eight weekly psychodrama sessions [91]	Mental health promotion	Uncontrolled	Clinical Adolescents and young adults living with HIV attending supportive services in Beira	N = 35 15 - 24 years	Statistically significant improvement in mental health knowledge and alexithymia *(no measures of effect size)*	**High** No control group; researchers not blinded; lack of valid instrument; direct distal outcomes were not reported	Part of Doctors with Africa CUAMM, supported by the Italian Agency for Development Cooperation (AICS) through the Global Fund project
Individual or pair activities for psychomotor and adaptive skills (twice-weekly 45-min sessions over 4 months) [92]	Neurodevelopment	Uncontrolled	Clinical Children with developmental disorders institutionalized in a rehabilitation center in Maputo	N = 6 6 - 13 years	Significant improvement body awareness, gross motor skills, and adaptive skills *(no measures of effect size)*	**High** No control group; limited sample size; unblinded; proxy-reported measures; multiple statistical tests without prior hypothesis or adjustments	Not reported

Notes: *Methodological quality was assessed with the revised Cochrane tool for randomized trials (RoB 2) for the single RCT; the remaining assessments were based on the Joanna Briggs Institute (JBI) checklist for non-randomized designs [22,23].

^1^Post-hoc case-control design: 75 caregivers received the intervention, and 62 caregivers formed the control group. ^2^ The mean number of children per caregiver was 4·34, but analysis included only one child, focusing on the one closer to 4·4 years selecting

**Abbreviations:** RCT (Randomized Clinical Trial); RoB (Risk of Bias).

The RCT assessed a World Bank program among 1,907 preschool children in high-poverty areas [31]. It demonstrated significant developmental gains with an implementation cost of approximately $3 per child per month. However, it was judged to have a high risk of bias across all assessed domains, including selection bias, blinding of participants and outcome assessors, outcome measurement, and selective outcome reporting.

The single study focusing on mental health symptoms trained community health workers to deliver a psychodrama intervention for adolescents and young adults living with HIV+ [91]. Although lacking a control group, pre-post assessment with 35 participants showed statistically significant improvement in mental health knowledge and alexithymia.

## Discussion

This systematic review synthesizes evidence on child and adolescent mental health in Mozambique, encompassing 61 reports from 25 prevalence studies, 29 reports describing 42 assessment instruments, and 8 intervention trials. The prevalence of mental disorders according to structured clinical criteria was only assessed at regional samples, including a survey with adolescents in Maputo (any mental health diagnosis: 23%) and another study with children and adolescents in Nampula (ADHD: 13·4%) [8,57–60]. Nationally representative surveys report mental health-related outcomes and risk factors, indicating that one in four adolescents experience physical or emotional violence annually, while 16·55% report lifetime sexual abuse, with particular concern regarding gender-based violence against girls [32–53,56,62,67–70]. National suicide rates per age group could be estimated via epidemiological registers (15–19 years: 1·53; 10–14 years: 0·59; 5–9 years: 0·05) [28]. Only one clinical interview (MINI-KID) and three screening instruments (GAD-7, PHQ-A, and SDQ) have undergone cross-cultural adaptation and positive psychometric evaluation across multiple properties, while tools lacking cultural adaptation showed poor performance [8,55,57,59–61,63,82]. Among interventions, only one RCT was identified, targeting neurodevelopment in high-poverty settings and showing positive outcomes and cost-effectiveness [31].

With significant data gaps, the current evidence in Mozambique is far from conclusive, warranting caution when drawing comparisons with international indicators. Clinical data is limited to two urban samples comprising a total of 1,234 participants, yet point to a higher prevalence of mental health disorders compared to global estimates assessed with similar manualized criteria [10,93]. Although suicide rates in Mozambique appear lower than then global averages (ages 15–19: 1.53; ages 10–14: 0.59 per 100,000), they are likely underestimated [94]. The single national study was based on three referral services responsible for all violent death autopsies in the country [28]. Moreover, the reliability of epidemiological and civil registration data in Mozambique is limited, with reports that nearly half of children under the age of five are not registered in official records [3]. Similarly, other register-based studies in Mozambique are scarce and rely primarily on service-level data from isolated facilities, limiting their generalizability. An integrated national health information system that includes mental health outcomes is needed to strengthen epidemiological data and monitoring across the country [95].

National-level data with adolescent samples indicate alarming rates of exposure to adversities and emotional and behavioral symptoms [32–53]. In particular, the high prevalence of physical, emotional, and sexual violence stands out as a clear public health priority. These experiences are not only key social determinants of psychological distress and mental disorders, but also undermine healthy development and long-term prospects, potentially perpetuating cycles of violence and trauma across future generations [96–99]. While current data in Mozambique is restricted to adolescents, it is likely that such exposures begin at younger age groups. Surveys including infants, preschoolers, and school-aged children are recommended to support data-driven action from the earliest possible stages of development [97]. It is worth noting that Mozambique has made progress in strengthening child protection mechanisms, with special consideration to gender-based violence and sexual exploitation [3,67]. The National Mental Health Strategy and Action Plan (2016–2026) also emphasizes interinstitutional cooperation with child protection bodies as a priority area [100].

We identified substantial gaps in the cross-cultural adaptation and psychometric validation of mental health instruments in Mozambique. While such gaps are common across non-English-speaking countries, they are particularly pronounced in Mozambique, where only 42 tools were identified (compared to 912 in Brazil and 261 in Greece) [9,10]. As a Portuguese-speaking country, Mozambique has often relied on instruments translated for use in Brazil or Portugal without further cultural adaptation, which was linked to poor psychometric performance in a few validation studies [59–61,82]. This highlights the need to adhere to culturally-sensitive practices in the country, including translating tools to local dialects; to this end, it is necessary to prioritize locally trained specialists over the current reliance on international experts [101].

The volume and scope of scientific literature on child and adolescent mental health in Mozambique remain limited, presenting a recent research body in which most output emerged in the last decade. Reflecting the country’s socioeconomic challenges, existing studies largely focus on adversities such as maltreatment and violence, with some research clustered around neurodevelopment in vulnerable populations [31–53,61,84,85]. While this underscores the elevated psychosocial burden and risk of mental disorders, few studies directly address mental health conditions. There are no nationally representative estimates of mental disorders based on standardized diagnostic criteria. Existing data are limited to a small number of disorders assessed in two localized samples from Maputo and Nampula, leaving other regions and rural areas unrepresented [8,57–60]. No prevalence estimates are reported for ASD or eating disorders, and no assessment tools are available for research or clinical practice on these conditions. This reflects the early stage of service development, with fewer than 5% of people experiencing mental health conditions receiving any form of care [101]. Future efforts should prioritize closing the data gap on estimates of both overall and specific mental disorders among children and adolescents, improving representation of national and regional samples.

The majority of scientific literature has been produced through international collaborations and funded by development agencies. Few local experts appear as first or lead authors, and only two dissertations have been published by a local university [28,29]. This reflects a scarcity of postgraduate programs and fragile academic infrastructure. Mozambique has only five medical schools, and no domestic capacity for postgraduate psychiatric training existed until 2018 (the first three Mozambican psychiatrists trained abroad graduated in 2004–2005) [101]. The need to strengthen national research capacity is acknowledged in the Strategy and Action Plan for Mental Health 2016–2026, which outlines an action front for developing research and human resources [4]. The Department of Mental Health has established partnerships with institutions in Portugal, Brazil, and the United States to support postgraduate training in mental health [102,103]. As a result, Mozambique now has 30 psychiatrists (10 PhDs, 6 Master’s), 693 psychologists (2 PhDs, 1 Master’s), and 41 occupational therapists (1 Master’s). Nevertheless, national universities remain weakly connected to medical specialization and offer few advanced training opportunities [104]. Eduardo Mondlane University has a Master’s in Mental Health, and some doctoral programs in Biosciences have recently integrated mental health [28,29,102]. Strengthening postgraduate training opportunities within the country is critical to building sustainable research capacity and a skilled mental health workforce.

This work builds on the methodological strengths of previous systematic reviews conducted in Greece, Brazil, and South Africa, employing a comprehensive search strategy and structured guidelines that provide best practices for appraising prevalence estimates, assessment instruments, and interventions [9–11]. We further increased reach by including studies involving clinical samples and screening institutional reports and local catalogues. Although screening was often conducted by a single reviewer, the emphasis on maximizing inclusion and identifying as many relevant studies as possible helped reduce the risk of missing important evidence, and no major discrepancies were identified during the independent verification of screening and data extraction (this likely reflects the reviewers’ prior experience with a similar review conducted in Brazil, as well as pilot calibration exercise and ongoing discussions throughout the review process).

However, this review does not capture the totality of mental health research on children and adolescents in Mozambique. Additional data may be scattered across uncaptured sources, including multinational assessments reporting only aggregate results, non-academic sources, and conference abstracts [105]. Given the limited scope of local research, these sources could represent a substantial body of evidence. Meta-analysis was not feasible due to the limited number and substantial heterogeneity of the included studies, which yielded few comparable data points. For instance, no single prevalence estimate or measurement property was evaluated across two or more studies with comparable samples (notably the two community-based studies using clinical interviews did not report on the same mental disorders, further relying on distinct diagnostic manuals and age ranges). This heterogeneity also precluded a formal certainty-of-evidence assessment (e.g., GRADE) given the lack of overlap across reports.

The field of child and adolescent mental health is still emerging in Mozambique. While available data is scarce, it suggests a high burden of mental disorders, with public health priorities shaped by widespread socioeconomic adversity and alarming rates of gender-based violence. There are few available resources for screening mental health symptoms, and reliable assessment warrants cultural adaptation of instruments. Future research should target data gaps on prevalence rates of mental disorders across regional and national samples. There is an urgent need to strengthen local research capacity through investment in human resource development and the expansion of academic institutions.

## Supporting information

S1 Appendix Fig A. PRISMA flowchart. Table A. PRISMA checklist. Table B. Search strategy and query per database. Table C. Prevalence studies: extracted information for each estimate. Table D. Data extracted for each instrument reported at each study. Table E. Instruments: evaluation of psychometric properties and language. Table F. Intervention studies: extracted information. Table G. Synthesis of each instrument property: coding criteria. Table H. Reasons for exclusion. Table I. Mental-health related findings across epidemiological, clinical, and administrative registers. Table J. Instruments that were applied in Mozambique (without adaptation or validation).
(DOCX)

S1 Data Screening documentation. Original search files and report of all screening decisions.
(ZIP)

S2 Data Extraction sheets. All included studies, extracted data and risk of bias assessment.
(XLSX)
