# Supplementary material for: The science of child and adolescent mental health in Mozambique: A systematic review of prevalence estimates, measurement tools, and interventions
Source: PLOS Glob Public Health. 2026 Oct 23;6(10):e0007513. doi: 10.1371/journal.pgph.0007513 (PMC13693922; doi:10.1371/journal.pgph.0007513)
Supplement: Supplementary file 1 [file pgph.0007513.s001.docx]

S1 Appendix

The science of child and adolescent mental health in Mozambique: a systematic review of prevalence estimates, measurement tools, and interventions

# Table of Contents

Fig A. PRISMA flowchart	2

Table A. PRISMA checklist	3

Table B. Search strategy and query per database	6

Table C. Prevalence studies: extracted information for each estimate	8

Table D. Data extracted for each instrument reported at each study	9

Table E. Instruments: evaluation of psychometric properties and language	11

Table F. Intervention studies: extracted information	12

Table G. Synthesis of each instrument property: coding criteria	13

Table H. Reasons for exclusion	14

Table I. Mental-health related findings across epidemiological, clinical, and administrative registers	15

Table J. Instruments that were applied in Mozambique (without adaptation or validation)	17

Reference list for supporting material	18

## Fig A. PRISMA flowchart

**Notes: ***This is a raw sum of reasons for exclusion across prevalence, instrument, and intervention reports screenings; see S1 Appendix Table H for detailed reasons according to each specific area; **Records**: any entry/result registered during screening. **Report**: any specific document or publication. **Study**: underlying research project (database survey or clinical trial) that may ground multiple reports. PRISMA flowchart is licensed under CC BY 4.0. To view a copy of this license, visit <https://creativecommons.org/licenses/by/4.0/>. Source: Page MJ, et al. BMJ 2021;372:n71. doi: 10.1136/bmj.n71.

## Table A. PRISMA checklist

| **Section and Topic** | **Item #** | **Checklist item** | **Location where item is reported** |
| --- | --- | --- | --- |
| **TITLE** | | |  |
| Title | 1 | Identify the report as a systematic review. | #1 |
| **ABSTRACT** | | |  |
| Abstract | 2 | See the PRISMA 2020 for Abstracts checklist. | #2 |
| **INTRODUCTION** | | |  |
| Rationale | 3 | Describe the rationale for the review in the context of existing knowledge. | #3-4 |
| Objectives | 4 | Provide an explicit statement of the objective(s) or question(s) the review addresses. | #4 |
| **METHODS** | | |  |
| Eligibility criteria | 5 | Specify the inclusion and exclusion criteria for the review and how studies were grouped for the syntheses. | #5, #7, Supplementary Tables 3 to 7 |
| Information sources | 6 | Specify all databases, registers, websites, organisations, reference lists and other sources searched or consulted to identify studies. Specify the date when each source was last searched or consulted. | #6, Supplementary Table 2 |
| Search strategy | 7 | Present the full search strategies for all databases, registers and websites, including any filters and limits used. | #6, Supplementary Table 2 |
| Selection process | 8 | Specify the methods used to decide whether a study met the inclusion criteria of the review, including how many reviewers screened each record and each report retrieved, whether they worked independently, and if applicable, details of automation tools used in the process. | #6-7 |
| Data collection process | 9 | Specify the methods used to collect data from reports, including how many reviewers collected data from each report, whether they worked independently, any processes for obtaining or confirming data from study investigators, and if applicable, details of automation tools used in the process. | #7-8, Supplementary Table 2 |
| Data items | 10a | List and define all outcomes for which data were sought. Specify whether all results that were compatible with each outcome domain in each study were sought (e.g. for all measures, time points, analyses), and if not, the methods used to decide which results to collect. | #5, #7-8, Supplementary Tables 2 to 7 |
|  | 10b | List and define all other variables for which data were sought (e.g. participant and intervention characteristics, funding sources). Describe any assumptions made about any missing or unclear information. | #5, #7-8, Supplementary Tables 2 to 7 |
| Study risk of bias assessment | 11 | Specify the methods used to assess risk of bias in the included studies, including details of the tool(s) used, how many reviewers assessed each study and whether they worked independently, and if applicable, details of automation tools used in the process. | #7-8 |
| Effect measures | 12 | Specify for each outcome the effect measure(s) (e.g. risk ratio, mean difference) used in the synthesis or presentation of results. | #7-8, Supplementary Tables 2-7 |
| Synthesis methods | 13a | Describe the processes used to decide which studies were eligible for each synthesis (e.g. tabulating the study intervention characteristics and comparing against the planned groups for each synthesis (item #5)). | #6-8 |
|  | 13b | Describe any methods required to prepare the data for presentation or synthesis, such as handling of missing summary statistics, or data conversions. | #6-8 |
|  | 13c | Describe any methods used to tabulate or visually display results of individual studies and syntheses. | #7-8, Supplementary Tables 6 to 7 |
|  | 13d | Describe any methods used to synthesize results and provide a rationale for the choice(s). If meta-analysis was performed, describe the model(s), method(s) to identify the presence and extent of statistical heterogeneity, and software package(s) used. | #7-8 |
|  | 13e | Describe any methods used to explore possible causes of heterogeneity among study results (e.g. subgroup analysis, meta-regression). | Not applicable |
|  | 13f | Describe any sensitivity analyses conducted to assess robustness of the synthesized results. | Not applicable |
| Reporting bias assessment | 14 | Describe any methods used to assess risk of bias due to missing results in a synthesis (arising from reporting biases). | Not applicable |
| Certainty assessment | 15 | Describe any methods used to assess certainty (or confidence) in the body of evidence for an outcome. | Not applicable |
| **RESULTS** | | |  |
| Study selection | 16a | Describe the results of the search and selection process, from the number of records identified in the search to the number of studies included in the review, ideally using a flow diagram. | #8, Figure 1, Supplementary Figure 1 |
|  | 16b | Cite studies that might appear to meet the inclusion criteria, but which were excluded, and explain why they were excluded. | #5, #8,, Supplementary Tables 8 and 10 |
| Study characteristics | 17 | Cite each included study and present its characteristics. | #8-26, Tables 1 to 4, Supplementary Table 9 |
| Risk of bias in studies | 18 | Present assessments of risk of bias for each included study. | #8-26, Tables 1 to 4, Supplementary Table 9 |
| Results of individual studies | 19 | For all outcomes, present, for each study: (a) summary statistics for each group (where appropriate) and (b) an effect estimate and its precision (e.g. confidence/credible interval), ideally using structured tables or plots. | #8-26, Tables 1 to 4, Supplementary Table 9 |
| Results of syntheses | 20a | For each synthesis, briefly summarise the characteristics and risk of bias among contributing studies. | #8-26, Tables 1 to 4, Supplementary Table 9 |
|  | 20b | Present results of all statistical syntheses conducted. If meta-analysis was done, present for each the summary estimate and its precision (e.g. confidence/credible interval) and measures of statistical heterogeneity. If comparing groups, describe the direction of the effect. | Not Applicable |
|  | 20c | Present results of all investigations of possible causes of heterogeneity among study results. | Not Applicable |
|  | 20d | Present results of all sensitivity analyses conducted to assess the robustness of the synthesized results. | Not Applicable |
| Reporting biases | 21 | Present assessments of risk of bias due to missing results (arising from reporting biases) for each synthesis assessed. | Not Applicable |
| Certainty of evidence | 22 | Present assessments of certainty (or confidence) in the body of evidence for each outcome assessed. | Table 2 |
| **DISCUSSION** | | |  |
| Discussion | 23a | Provide a general interpretation of the results in the context of other evidence. | #27-29 |
|  | 23b | Discuss any limitations of the evidence included in the review. | #27-29 |
|  | 23c | Discuss any limitations of the review processes used. | #29 |
|  | 23d | Discuss implications of the results for practice, policy, and future research. | #27-30 |
| **OTHER INFORMATION** | | |  |
| Registration and protocol | 24a | Provide registration information for the review, including register name and registration number, or state that the review was not registered. | #4 |
|  | 24b | Indicate where the review protocol can be accessed, or state that a protocol was not prepared. | #4 |
|  | 24c | Describe and explain any amendments to information provided at registration or in the protocol. | #4 |
| Support | 25 | Describe sources of financial or non-financial support for the review, and the role of the funders or sponsors in the review. | #30 |
| Competing interests | 26 | Declare any competing interests of review authors. | #30 |
| Availability of data, code and other materials | 27 | Report which of the following are publicly available and where they can be found: template data collection forms; data extracted from included studies; data used for all analyses; analytic code; any other materials used in the review. | #4 |

*From:* Page MJ, McKenzie JE, Bossuyt PM, Boutron I, Hoffmann TC, Mulrow CD, et al. The PRISMA 2020 statement: an updated guideline for reporting systematic reviews. BMJ 2021;372:n71. doi: 10.1136/bmj.n71. This work is licensed under CC BY 4.0. To view a copy of this license, visit <https://creativecommons.org/licenses/by/4.0/>

## Table B. Search strategy and query per database

| **Database / Repositoire** | **Date** | **Strategy** | Query |
| --- | --- | --- | --- |
| Pubmed  PsycInfo  Web of Science  CINAHL | August 6th, 2026 | Full query English terms (syntax adapted per databases)  Results are exported and deduplicated  Two independent reviewers perform primary (title/abstract). A single review performs secondary screening (full text). | *(adolesc* OR preadolesc* OR pre-adolesc* OR child* OR boy OR girl OR infant* OR juvenil* OR minors OR paediatri* OR pediatri* OR pubescen* OR puberty OR school* OR student* OR teen* OR young OR youth* OR class* OR orphan* OR high-school OR "high school" OR preschool* OR pre-school*)*  ***AND*** *(Mozambique OR Mozambican OR Moçambique)*  ***AND*** *(Mental OR "Mental health" OR "Mental Disorder" OR Psychiatr* OR Psycho* OR behavior OR Mood OR Emotion OR Cognitive OR Neurocognitive OR Autism OR Enuresis OR Encopresis OR ADHD OR "Attention Deficit Hyperactivity Disorder" OR Intellectual disability OR development delay OR neurodevelopment OR Oppositional Defiant OR Conduct OR Depression OR depressive OR Bipolar OR Suicide OR Suicidality OR Selfharm OR Obsessive-compulsive OR Trauma OR PTSD OR Mutism OR "Substance use" OR Cannabis OR Alcohol OR Tobacco OR Anorexia OR Bulimia OR "Eating disorder" OR "Personality Disorder" OR Schizophrenia OR Psychosis OR "Quality of life" OR wellbeing OR "learning disorder" OR "learning delay" OR anxi* OR phob* OR panic)** |
| African Index Medicus | August 6th, 2026 | Simplified query in English and Portuguese  Single-reviewer screening of all results | *Mozambique OR Moçambique* |
| Google Scholar | August 6th, 2026 | Simplified queries in English and Portuguese  A single reviewer screening results until reaching 100 sequential articles without novel inclusions | ***1. English search:*** *Mozambique (Mental OR Psychiatry OR Psychology) (Child OR Adolescent OR school)*  ***2. Portuguese search****: Moçambique (Mental OR Psico* OR Psiq*) (Criança OR adolescente OR escola OR infancia OR juventude)* |
| Universidade Eduardo Modlane (UEM) | August 6th, 2026 | Simplified query in Portuguese  Single-reviewer screening of all results | *adolescente OR pré-adolescente OR criança OR menino OR menina OR bebê OR juvenil OR menores OR pediátrico OR puberdade OR escola OR estudante OR adolescente OR jovem OR juventude OR órfão OR "ensino médio" OR "escola secundária"* |
| Instituto Nacional de Saúde (INS)  Universidade Lúrio (UniLurio)  Revista Médica de Moçambique  Revista Moçambicana de Ciência de Saúde | August 6th, 2026 | Single-reviewer screening of all publications |  |

## Table C. Prevalence studies: extracted information for each estimate

| **Topic** | **Variable** |
| --- | --- |
| **Study details** | First author |
| **Study details** | Year of publication |
| **Study details** | Study description |
| **Study details** | Region |
| **Data of each estimate** | Diagnostic domain |
| **Data of each estimate** | Condition or construct |
| **Data of each estimate** | Year of data collection |
| **Data of each estimate** | Sampling/representativeness |
| **Screening sample** *(if available)* | Instrument |
| **Screening sample** *(if available)* | Cut-off for positive |
| **Screening sample** *(if available)* | Informant |
| **Screening sample** *(if available)* | Recruitment method |
| **Screening sample** *(if available)* | Sample size |
| **Screening sample** *(if available)* | Response rate |
| **Screening sample** *(if available)* | Age range |
| **Screening sample** *(if available)* | Percentage of males |
| **Screening sample** *(if available)* | Number positive |
| **Diagnostic sample** | Sample description |
| **Diagnostic sample** | Sample size |
| **Diagnostic sample** | Response rate |
| **Diagnostic sample** | Percentage of males |
| **Diagnostic sample** | Assessment instrument |
| **Diagnostic sample** | If instrument includes interview |
| **Diagnostic sample** | Informants |
| **Diagnostic sample** | Diagnostic criteria |
| **Diagnostic sample** | If diagnosis requires functional impairment |
| **Diagnostic sample** | Definition of functional impairment |
| **Diagnostic sample** | Type of prevalence |
| **Results** | Prevalence estimate (SD or 95% CI) |

## Table D. Data extracted for each instrument reported at each study

| **Topics / Procedures** | **Extracted information** |
| --- | --- |
| General information | Details on the study, instrument, and sample |
| Study procedures | Whether it develops, validates and/or translates, or only applies an instruments |
| Development quality* | Methodological quality** |
| Content validity quality* | Methodological quality** |
| Instrument translation | The presence of procedures such as back-and-forth translation, independent translators, expert committee assessment, and pilot testing |
| Structural validity | Type of analysis (e.g., exploratory factor analysis, principal component analysis, or confirmatory factor analysis)  How many factors best accounted for how much of the variance  Results of statistical procedures (e.g., Root Mean Square Error of Approximation (RMSEA), Comparative Fit Index (CFI), Standardized Root Mean Residuals (SRMS))  Sample size and methodological quality** |
| Internal consistency | Cronbach’s alpha or equivalent measures  Sample size and methodological quality** |
| Cross-cultural validity | Multi-group confirmatory factor analysis with measurement invariance evaluation  Sample size and methodological quality** |
| Inter-rater reliability | Intraclass correlation coefficient, pearson or spearman correlation, kappa score, or equivalent statistics  Sample size and methodological quality** |
| Test-retest reliability | Intraclass correlation coefficient, pearson or spearman correlation, kappa score, or equivalent statistics  Sample size and methodological quality** |
| Measurement error | Statistics on the patient’s score error (e.g., standard error measurement, smallest detectable change, or limits of agreement)  Sample size and methodological quality** |
| Criterion validity | Performance of the instrument against a gold standard (e.g., area under the curve, sensitivity, and specificity)  Sample size and methodological quality** |
| Construct validity | Correlation coefficients with other instruments  Sample size and methodological quality** |
| Responsiveness | Detection of statistically significant differences in measures over time (e.g., after an intervention)  Sample size and methodological quality** |

**Notes:** This table is reproduced from Marchionatti et al. (2026) [(1)](https://paperpile.com/c/n4yb50/oGVyR)**.** *Only for studies reporting instrument development. **Classified as very good, adequate, doubtful, inadequate, or not applicable, following the Consensus-based Standards for the Selection of Health Measurement Instruments (COSMIN) guidelines [(2)](https://paperpile.com/c/n4yb50/QF3Yi).

## Table E. Instruments: evaluation of psychometric properties and language

| **Code** | **Criteria for each psychometric property evaluation** |
| --- | --- |
| + | The property was measured and the study provides positive evidence according to standard definitions from COSMIN, except for adapted definition for responsiveness   - **Structural validity** with CFA with CFI >0.95/RMSEA <0.06 - **Internal consistency** with Cronbach's alpha > 0.7 - **Reliability** with Pearson’s *r*, ICC or weighted Kappa ≥ 0.70 - **Cross-cultural validity** with no differences between group factors; reliability with ICC or Kappa >= 0.70 - **Measurement error** with SDC or LoA < MIC - **Criterion validity** with correlation with gold standard >= 0.70 - **Construct validity** with correlations superior 0.10 or 0.5, depending on the hypothesis - **Responsiveness** statistically significant differences between time-points |
| - | The property was measured and the study provides negative evidence according to standard definitions (e.g., Cronbach's alpha < 0.7 for internal consistency, confirmatory factor analyses with CFI <0.95/RMSEA >0.06 for structural validity) |
| +/- | Mixed outcomes that may be classified either as “+” or “-” |
| ? | Information was provided for the property, but it is insufficient to ascertain it in previous categories |
| NA | Information was not available for that property |
| **Code** | **Criteria for study language and procedures** |
| **D** | Development study (in Portuguese or other local language) |
| + | Study translates the instrument with structured procedures as back-and-forth translation, independent translators, expert committee assessment, and pilot testing |
| - | Study translated the instrument without structured procedures |
| ? | Employs a previously translated version of the instrument |

**Notes**: This table is reproduced from Marchionatti et al. (2024) [(1)](https://paperpile.com/c/n4yb50/oGVyR).. The summary appraisal table evaluates data from the extraction table, with each entry corresponding for each instrument procedure at each study. Refer to Consensus-based Standards for the selection of health Measurement Instruments Manual (COSMIN) for details of criteria [(2)](https://paperpile.com/c/n4yb50/QF3Yi). For responsiveness, we considered sufficient (“+”) evidence of validation if the instrument presents statistically significant differences in a clinical trial. This procedure was chosen because studies did not provide the area under the curve in this property, which was the original parameter in the COSMIN manual. **Abbreviations**: Confirmatory Factor Analysis (CFA), Comparative Fit Index (CFI), Intraclass Correlation Coefficient (ICC), Limits of Agreement (LoA), Minimal Important Change (MIC), Root Mean Square Error of Approximation (RMSEA), Smallest Detectable Change (SDC).

## Table F. Intervention studies: extracted information

| Field | Details |
| --- | --- |
| Year |  |
| Author |  |
| Condition |  |
| Design | RCT, controlled, uncontrolled, adaptation or development protocol |
| Type of intervention | Psychosocial, life style or biomedical |
| Experimental interventions |  |
| Control Interventions |  |
| Target construct |  |
| Primary outcome |  |
| Primary outcome measurement |  |
| Secondary outcome |  |
| Secondary outcome measurement |  |
| Other measures |  |
| Population description |  |
| Sample (age, gender, race, location) |  |
| Inclusion criteria |  |
| Exclusion criteria |  |
| Method of recruitment |  |
| Allocation methods | Include unit of allocation (cluster or individual) |
| Size per group | Intervention and control, total number randomized |
| Baseline imbalances |  |
| Duration of intervention |  |
| Withdrawals and exclusion |  |
| Other treatment received |  |
| Time points measured |  |
| Informant |  |
| Effect size | Controlled and uncontrolled |
| Results |  |
| Demonstrated efficacy? |  |
| Risk of bias[(3,4)](https://paperpile.com/c/n4yb50/DUfwR+pw8H4) | The revised Cochrane tool for randomized trials (RoB 2)  The Joanna Briggs Institute (JBI) tool for non-randomized designs |
| Funding |  |

## Table G. Synthesis of each instrument property: coding criteria

| **Code** | **Criteria for psychometric properties** |
| --- | --- |
| + | Available reports with positive validation results on that psychometric property |
| +/- | Available reports with both positive and negative validation results on that psychometric property |
| - | Available reports with negative validation results on that psychometric propertie |
| ? | Available reports for that psychometric property that cannot be classified as positive or negative |
| NA | No available reports for that psychometric property |
| **Code** | **Criteria for study language and procedures** |
| D | An instrument developed in Portuguese or other local language with reports on its development |
| PT-BR | Instruments originally in Portuguese or other local language without reports on its development |
| P | Instruments with a previous translation to Portuguese or other local language, without reports on its procedures |
| + | Instruments that were translated or cross-culturally adapted to Portuguese or other local language with at least one of the following procedures: back-and-forth translation, independent translators, expert committee assessment, and pilot testing |
| - | Instruments that were freely translated to Portuguese or other local language |

**Note:** This table is reproduced from Marchionatti et al. (2024) [(1)](https://paperpile.com/c/n4yb50/oGVyR). The synthesis table aggregates all information reported for each instrument across extracted studies.

## Table H. Reasons for exclusion

**Primary Screening**

Wrong outcome: 4192

Wrong population: 194

Wrong study design: 47

Wrong publication type: 17

Background article: 1

**Secondary Screening: Prevalence Studies**

Wrong population: 211

Wrong outcome: 70

Wrong study design: 33

Background/secondary article: 19

Wrong publication type: 12

Full report unavailable 7

**Secondary Screening: Instrument Studies**

Wrong population: 137

Wrong study design: 127

Only applies an instrument already Included: 70

Wrong outcome: 37

Background article: 8

Wrong publication type: 5

**Secondary Screening: Intervention Studies**

Wrong study design: 29

Wrong population: 13

Wrong publication type: 5

Wrong outcome: 4

Full report unavailable: 3

Background article: 1

## **Table I. Mental-health related findings across epidemiological, clinical, and administrative registers**

| **REGISTER-BASED STUDIES** | | | | | |
| --- | --- | --- | --- | --- | --- |
| Study | Methods | Sample representativeness | Location  Year | Diagnostic criteria | Findings |
| [^(5)^](https://paperpile.com/c/n4yb50/9ZTGt) | Record book of autopsies at Forensic Services | National population  All suicides should undergo autopsy and data was collected at all forensic central services attending Mozambique at the time | Central Hospitals of Maputo, Beira and Nampula  2014 - 2019 | Autopsy registers | Absolute number of suicides   - 5 - 19 years-old: 70 - 5 - 9 years-old: 2 - 10 - 14 years-old: 21 - 15 - 19 years-old: 47   *Suicide rates per 100,000:^1^*   - *5 - 19 years-old: 0.65* - *5 - 9 years-old: 0.05* - *10 - 14 years-old: 0.59* - *15 - 19 years-old: 1.53* |
| [^(6)^](https://paperpile.com/c/n4yb50/n5L0b) | Record book of autopsies at a regional forensic service  Emergency visits records at a regional hospital | Sofala and adjacent catchment area of Beira Central Hospital | Beira Central Hospital in Sofala  2013 - 2014 | Autopsy registers of suicide  Clinical registers of emergency visits | Absolute number of suicides in (up to 18 years-old): 19  Emergency visits due to suicidality (up to 18 years-old): 36 |
| [^(7)^](https://paperpile.com/c/n4yb50/MDJrP) | Data is collected from statistical tables of the The State of the World's Children 2016, which are currently unavailable | National  Data is currently unavailable in the dataset | Unclear | Unclear | Child labor: 22%  Child marriage (married by 15): 14%  Child marriage (married by 18): 48%  Child neglect (children with inadequate supervision): 33% |
| [^(8)^](https://paperpile.com/c/n4yb50/KPyAC) | Clinical registers from the four regional referral hospitals in Mozambique | Nationwide  Age: 0 - 14 years | Hospitals in Maputo, Beira, Quelimane and Nampula  2019 - 2020 | Clinical registers of paediatric patients admitted with acute injuries | Injury hospital admission due to interpersonal violence:   - 2019: 5 cases - 2020: 4 cases |
| [^(9)^](https://paperpile.com/c/n4yb50/8fKr8) | Clinical registers from a regional hospital | Maputo Central Hospital catchment area  Age: 0 - 14 years | Maputo Central Hospital  2019 | Clinical registers of paediatric patients admitted with acute injuries | Hospital visit due to physical violence: 64 cases  Hospital visit due to sexual violence: 79 cases |
| [^(10)^](https://paperpile.com/c/n4yb50/IwqMu) | Clinical registers from a regional hospital | Hospital Central da Beira catchment area  Age: 0 - 14 years | Hospital Central da Beira  2017 - 2018 | Clinical registers of paediatric patients admitted with acute injury | Hospital visit due to violent injury: 143 |
| [^(11)^](https://paperpile.com/c/n4yb50/Nlzom) | Clinical registers from a regional hospital | Hospital Central de Maputo catchment area  Age: 0 - 18 years | Hospital Central de Maputo  2015 - 2020 | Clinical registers from the Forensic Service | Child Sexual Abuse victims: 2610  93.7% girls  Assaults at the victims’ or perpetrator’s home: 54.8%  Perpetrator identified as an acquaintance: 72.9%  Concomitant physical violence: 54.1%  Perpetrator did not use condom: 74.9% |
| [^(12)^](https://paperpile.com/c/n4yb50/1SP3u) | Clinical sample  HIV-infected infants aged <12 months at the time of hospital admission who had never initiated ART | N = 27  Up to 1 year-old  2019 | Hospital Central de Maputo (HCM) and Hospital Central de Beira (HCB). | Clinical charts classified according to established guidelines for developmental milestones | Delayed developmental milestones: 58,06% |

**Notes:** ¹We estimated relative rates considering the median of Mozambique population in each age group, from 2014 to 2019, as per the United States Census Bureau [(13)](https://paperpile.com/c/n4yb50/Zb3Xb)*: 1.08 million (5-19 years); 4,2 million (5 - 9 years); 3,5 million (10 - 14 years); 3 million (15 - 19 years).*

## Table J. Instruments that were applied in Mozambique (without adaptation or validation)

| **Tools applied without providing data on psychometric validation** | |
| --- | --- |
| **Assessment instruments** | **Questionnaires from global surveys** |
| Children’s Global Assessment Scale (CGAS) [(14,15)](https://paperpile.com/c/n4yb50/VrwCX+YYaRt) | Demographic and Health Surveys (DHS) [(16,17)](https://paperpile.com/c/n4yb50/xXlxJ+qFpNF) |
| Global Scales for Early Development (GSED) [(18)](https://paperpile.com/c/n4yb50/Fmy7) | Global Youth Tobacco Survey (GYTS) [(19)](https://paperpile.com/c/n4yb50/sR7hv) |
| Alcohol, Smoking, and Substance Involvement Screening Test (ASSIST) [(14)](https://paperpile.com/c/n4yb50/VrwCX) | WHO Multi-country Survey of Women’s Health and Domestic Violence against Women [(20)](https://paperpile.com/c/n4yb50/eDm1L) |
| Pediatric Quality of LIfe Inventory (PedsQL)[(21)](https://paperpile.com/c/n4yb50/zCoSa) | Global School Health Survey (GSHS) [(22)](https://paperpile.com/c/n4yb50/yX8vt) |
| Eysenck Personality Questionnaire - Revised (EPQR)[(23)](https://paperpile.com/c/n4yb50/HzH1U) | Determined, Resilient, Empowered, AIDS-Free, Mentored, and Safe (DREAMS) Questionnaire [(24)](https://paperpile.com/c/n4yb50/Yc6P7) |
| Centre of Epidemiologic Studies Depression Scale (CES-D-10) [(25)](https://paperpile.com/c/n4yb50/XfUE) | Children’s Worlds Psychological Well-Being Scale (CW-PWBS) [(25)](https://paperpile.com/c/n4yb50/XfUE) |
| Paykel Suicide Scale [(25)](https://paperpile.com/c/n4yb50/XfUE) | UNICEF'sYouth Empowerment Project - Anxiety Scale [(25)](https://paperpile.com/c/n4yb50/XfUE) |
| Beck Anxiety Inventory (BAI) [(23)](https://paperpile.com/c/n4yb50/HzH1U) |  |
| Bateria Psicomotora (BPM) [(26)](https://paperpile.com/c/n4yb50/IjqNI) |  |
| Portuguese Adaptive Behavior Scale (PABS) [(26)](https://paperpile.com/c/n4yb50/IjqNI) |  |
| Child PTSD Symptom Scale for DMS-V (CPSS-V) [(27)](https://paperpile.com/c/n4yb50/6sHRO) |  |
| SCL-90 Symptom Checklist-90 [(28)](https://paperpile.com/c/n4yb50/kNu6) |  |
| Conflict and Tactics Scale (CTS)* [(29)](https://paperpile.com/c/n4yb50/wHlZ1) |  |

**Note:** Chaquisse 2018 Sexual and physical: Conflict and Tactics Scale (CTS) previously adapted to Mozambique, but for adult populatio

## Reference list for supporting material

1.	[Marchionatti LE, Campello AC, Veronesi JA, Ziebold C, Tonon AC, Casella CB, et al. The science of child and adolescent mental health in Brazil: a nationwide systematic review and compendium of evidence-based resources [Internet]. medRxiv. 2024. Available from:](http://paperpile.com/b/n4yb50/oGVyR) <https://www.medrxiv.org/content/10.1101/2024.11.10.24317061.abstract>

2.	[Prinsen CAC, Mokkink LB, Bouter LM, Alonso J, Patrick DL, de Vet HCW, et al. COSMIN guideline for systematic reviews of patient-reported outcome measures. Qual Life Res [Internet]. 2018 May;27(5):1147–57. Available from:](http://paperpile.com/b/n4yb50/QF3Yi) <http://dx.doi.org/10.1007/s11136-018-1798-3>

3.	[Sterne JAC, Savović J, Page MJ, Elbers RG, Blencowe NS, Boutron I, et al. RoB 2: a revised tool for assessing risk of bias in randomised trials. BMJ [Internet]. 2019 Aug 28;366:l4898. Available from:](http://paperpile.com/b/n4yb50/DUfwR) <http://dx.doi.org/10.1136/bmj.l4898>

4.	[Aromataris E, Stern C, Lockwood C, Barker TH, Klugar M, Jadotte Y, et al. JBI series paper 2: tailored evidence synthesis approaches are required to answer diverse questions: a pragmatic evidence synthesis toolkit from JBI. J Clin Epidemiol [Internet]. 2022 Apr 14; Available from:](http://paperpile.com/b/n4yb50/pw8H4) <http://dx.doi.org/10.1016/j.jclinepi.2022.04.006>

5.	[Magaia-Jamela IPA. Perfil e tendência dos casos de suicídio admitidos nos Serviços de Medicina Legal dos Hospitais Centrais de Maputo, Beira e Nampula no período de 2014 a 2018 [Internet] [Dissertation]. Augusto G, editor. [Maputo]: Universidade Eduardo Mondlane; 2023. Available from:](http://paperpile.com/b/n4yb50/9ZTGt) <http://www.repositorio.uem.mz/jspui/handle/258/1193>

6.	[Wagenaar BH, Raunig-Berhó M, Cumbe V, Rao D, Napúa M, Sherr K. Suicide Attempts and Deaths in Sofala, Mozambique, From 2011 to 2014. Crisis [Internet]. 2016;37(6):445–53. Available from:](http://paperpile.com/b/n4yb50/n5L0b) <http://dx.doi.org/10.1027/0227-5910/a000383>

7.	[Kobulsky JM, Dubowitz H, Xu YF. The global challenge of the neglect of children. Child Abuse Negl [Internet]. 2020;110. Available from:](http://paperpile.com/b/n4yb50/MDJrP) <http://dx.doi.org/10.1016/j.chiabu.2019.104296>

8.	[Amado V, Trott S, Möller J, Couto MT, Wallis L, Laflamme L. Changing patterns in the burden of paediatric injuries during the COVID-19 pandemic: a study in Mozambique’s central hospitals. BMC Health Serv Res [Internet]. 2023;23(1):1071. Available from:](http://paperpile.com/b/n4yb50/KPyAC) <http://dx.doi.org/10.1186/s12913-023-10073-x>

9.	[Nhassengo SK, Matsinhe SO, Jethá E, Laflamme L. Circumstances and Consequences of Violence-Related Injuries Presenting at Hospital. A Study at the Pediatric Emergency and Forensic Medicine Units of Maputo Central Hospital, Mozambique. Int J Environ Res Public Health [Internet]. 2021;18(22). Available from:](http://paperpile.com/b/n4yb50/8fKr8) <http://dx.doi.org/10.3390/ijerph182212125>

10.	[Brugnolaro V, Fovino LN, Calgaro S, Putoto G, Muhelo AR, Gregori D, et al. Pediatric emergency care in a low-income country: Characteristics and outcomes of presentations to a tertiary-care emergency department in Mozambique. PLoS One [Internet]. 2020;15(11):e0241209. Available from:](http://paperpile.com/b/n4yb50/IwqMu) <http://dx.doi.org/10.1371/journal.pone.0241209>

11.	[Matsinhe SO, Suffla S, Hector TJ. Occurrence and circumstances of child sexual assault in Maputo, Mozambique. J Forensic Leg Med [Internet]. 2024;108:102778. Available from:](http://paperpile.com/b/n4yb50/Nlzom) <http://dx.doi.org/10.1016/j.jflm.2024.102778>

12.	[Chaúque S, Mohole J, Zucula H, Lambo L, Lisboa A, Ferreira D, et al. HIV Encephalopathy in ART-Naïve, Hospitalized Infants in Mozambique. J Trop Pediatr [Internet]. 2021;67(6). Available from:](http://paperpile.com/b/n4yb50/1SP3u) <http://dx.doi.org/10.1093/tropej/fmab106>

13.	[United States Census Bureau. World Population Estimates and Projections [Internet]. International Database. 2024 [cited 2025 Jul 30]. Available from:](http://paperpile.com/b/n4yb50/Zb3Xb) <https://www.census.gov/programs-surveys/international-programs/about/idb.html>

14.	[Daniel HMC, Duarte I, Caye A, Suleman A, Fumo W, Rohde LA. Attention-deficit/hyperactivity disorder in Mozambique: an epidemiological investigation in a primary school sample. Rev Bras Psiquiatr [Internet]. 2024 Jan 19;46:e20233343. Available from:](http://paperpile.com/b/n4yb50/VrwCX) <https://www.bjp.org.br/details/2493/en-US/attention-deficit-hyperactivity-disorder-in-mozambique--an-epidemiological-investigation-in-a-primary-school-sample>

15.	[Daniel HMC, Duarte I, Caye A, Rohde LA. Exploring diagnostic performance of a screening instrument for ADHD and DSM-5 number of symptoms criterion in primary school students from Mozambique. J Atten Disord [Internet]. 2024 Mar;28(5):583–8. Available from:](http://paperpile.com/b/n4yb50/YYaRt) <http://dx.doi.org/10.1177/10870547231215290>

16.	[Wilson NL. Violence against female adolescents in low- and middle-income countries: Evidence from 36 national household surveys [Internet]. Vol. 36, Journal of Interpersonal Violence. Wilson, Nicholas L.: Reed College, 3203 SE Woodstock Blvd., Portland, OR, US, 97202, nwilson@reed.edu US: Sage Publications; 2021. p. NP4964–78. Available from:](http://paperpile.com/b/n4yb50/xXlxJ) <http://dx.doi.org/10.1177/0886260518792971>

17.	[Kebede S, Van Harmelen AL, Roman-Urrestarazu A. Wealth inequality and intimate Partner Violence: An individual and ecological level analysis across 20 countries. J Interpers Violence [Internet]. 2022 Sep;37(17-18):NP15568–93. Available from:](http://paperpile.com/b/n4yb50/qFpNF) <https://journals.sagepub.com/doi/10.1177/08862605211016337>

18.	[de Castro F, Momade K, Yourkavitch J, Arnold CD, Manhiça A, Zano F, et al. The impact of an integrated nurturing care intervention to improve early childhood development outcomes in Nampula Province, Mozambique. Public Health Nutr [Internet]. 2025 Jun 13 [cited 2026 Aug 6];29(1):e93. Available from:](http://paperpile.com/b/n4yb50/Fmy7) <http://dx.doi.org/10.1017/S1368980025100554>

19.	[Aboagye RG, Mohammed A, Duodu PA, Adnani QES, Seidu AA, Ahinkorah BO. Sex-related inequalities in current cigarette smoking among adolescents in Africa. Subst Abuse Treat Prev Policy [Internet]. 2024 Sep 5;19(1):41. Available from:](http://paperpile.com/b/n4yb50/sR7hv) <https://substanceabusepolicy.biomedcentral.com/articles/10.1186/s13011-024-00619-5>

20.	[Maguele MS, Tlou B, Taylor M, Khuzwayo N. Risk factors associated with high prevalence of intimate partner violence amongst school-going young women (aged 15-24years) in Maputo, Mozambique. PLoS One [Internet]. 2020 Dec 9;15(12):e0243304. Available from:](http://paperpile.com/b/n4yb50/eDm1L) <https://dx.plos.org/10.1371/journal.pone.0243304>

21.	[Issa F, Dang BN, Buck WC, Chicumbe S, Nicolau N, Virate C, et al. Quality of life assessments in a cohort of Mozambican children with sickle cell disease. Pan Afr Med J [Internet]. 2020 Aug 25;36:343. Available from:](http://paperpile.com/b/n4yb50/zCoSa) <http://dx.doi.org/10.11604/pamj.2020.36.343.24837>

22.	[Asante KO, Atorkey P. Cannabis and amphetamine use among school-going adolescents in sub-Saharan Africa: a multi-country analysis of prevalence and associated factors. BMC Psychiatry [Internet]. 2023 Oct 24;23(1):778. Available from:](http://paperpile.com/b/n4yb50/yX8vt) <http://dx.doi.org/10.1186/s12888-023-05283-w>

23.	[Gouveia L, Mandlate F, Ziebold C, Fumo W, Mabunda D, Lovero KL, et al. Emotional contagion behavior in a group of young girls in a secondary school in Maputo, Mozambique. Int J Soc Psychiatry [Internet]. 2023 Mar;69(2):447–53. Available from:](http://paperpile.com/b/n4yb50/HzH1U) <https://journals.sagepub.com/doi/10.1177/00207640221111265>

24.	[Howard AL, Chiang L, Picchetti V, Zhu L, Hegle J, Patel P, et al. Population estimates of HIV risk factors to inform HIV prevention programming for adolescent girls and young women. AIDS Educ Prev [Internet]. 2023 Jul;35(Supplement A):20–38. Available from:](http://paperpile.com/b/n4yb50/Yc6P7) <http://dx.doi.org/10.1521/aeap.2023.35.suppA.20>

25.	[Albajara Sáenz A, Kurten S, Saxton J, Kardefelt-Winther D, Ford T, Orben A, et al. Mental health measures among adolescents in 12 low- and middle-income countries: Measurement invariance and cross-sectional analyses of Disrupting Harm survey data. JCPP Adv [Internet]. 2025 Dec 13;(e70087):e70087. Available from:](http://paperpile.com/b/n4yb50/XfUE) <http://dx.doi.org/10.1002/jcv2.70087>

26.	[Silva AF, Santos S. As competências psicomotoras e adaptativas na qualidade de vida de crianças e jovens moçambicanos com perturbações do desenvolvimento: o contributo da intervenção psicomotora. Motricidade [Internet]. 2021;17(2):1–24. Available from:](http://paperpile.com/b/n4yb50/IjqNI) <https://search.ebscohost.com/login.aspx?direct=true&db=c8h&AN=153083471&site=ehost-live>

27.	[Nguyen N, Lovero KL, Falcao J, Brittain K, Zerbe A, Wilson IB, et al. Mental health and ART adherence among adolescents living with HIV in Mozambique. AIDS Care [Internet]. 2023 Feb;35(2):182–90. Available from:](http://paperpile.com/b/n4yb50/6sHRO) <https://www.tandfonline.com/doi/full/10.1080/09540121.2022.2032574>

28.	[Benoni R, Sartorello A, Malesani C, Cardoso H, Chaguruca I, Matope MDS, et al. How social-relational context impacts the mental health of adolescent and young adults living with and without HIV in Mozambique: A social network analysis study. J Epidemiol Glob Health [Internet]. 2025 Jun 10 [cited 2026 Aug 6];15(1):81. Available from:](http://paperpile.com/b/n4yb50/kNu6) <http://dx.doi.org/10.1007/s44197-025-00417-7>

29.	[Chaquisse E, Fraga S, Meireles P, Macassa G, Soares J, Mbofana F, et al. Sexual and physical intimate partner violence among women using antenatal care in Nampula, Mozambique. J Public Health Africa [Internet]. 2018 May 21;9(1):744. Available from:](http://paperpile.com/b/n4yb50/wHlZ1) <https://publichealthinafrica.org/index.php/jphia/article/view/934>
