# Supplementary material for: The science of child and adolescent mental health in Mozambique: A systematic review of prevalence estimates, measurement tools, and interventions
Source: PLOS Glob Public Health. 2026 Oct 23;6(10):e0007513. doi: 10.1371/journal.pgph.0007513 (PMC13693922; doi:10.1371/journal.pgph.0007513)
Supplement: Supplementary file 2 [file pgph.0007513.s005.docx]

Dear Editorial Office,

We would like to thank the Editor and the Reviewers for their careful examination of our manuscript and their constructive contributions. We have addressed all reviewer suggestions, which are detailed in the point-by-point response below. Overall, this revised manuscript incorporates the following main improvements:

- Updated literature search (rerun in August 2026)
- Full dataset available as supplementary material, including data extraction sheets and the complete risk of bias assessments.
- Full screening records available as supplementary material, including all search files and documentation of inclusion/exclusion decisions.

**[Methods]**

- Clarification over the rationale for selecting the scope of review (in terms of areas and outcomes)
- Clarification of the screening procedures (inclusion criteria and deduplication tools)
- New section (“Data synthesis and presentation”) outlining the framework for organizing the results and presenting the evidence catalogue

**[Results]**

- We clearly detailed study limitations and the overall quality of evidence, both in-text and across tables

**[Discussion]**

- We toned down some conclusions to align with the strength of evidence, discussing limitations in data quality and availability
- We link findings to policy frameworks in Mozambique

Next, we provide a comprehensive response to reviewers' concerns. Please refer to the following hyperlinks for direct access:

- **Journal requirements**
- **Reviewer #1**
- **Reviewer #2**
- **Revirewer #3**
- **Reviewer #4**

## JOURNAL REQUIREMENTS

*1. We note that the most recent literature search was completed over a year before the manuscript was submitted to the journal.*

**Answer:** We have updated all literature searches to include publications up to August 6, 2026. This yielded five new studies, including:

- A prevalence study reporting overall mental health disorder rates, derived from the dataset of a previously included study (see Table 1);
- A national survey conducted as part of the multinational Disrupting Harm initiative (see Table 2)
- A study reporting further data for Ages & Stages Questionnaire (ASQ), included as additional reference (see Table 3)
- An intervention study nested within a nutrition program (see Table 4)
- Data on new instruments applied without providing data on psychometric validation (see Table J in S1 Appendix)

We have updated the manuscript and supplementary materials to reflect these additions.

*2. Please amend your detailed Financial Disclosure statement. This is published with the article. It must therefore be completed in full sentences and contain the exact wording you wish to be published. a. Please clarify all sources of funding (financial or material support) for your study. List the grants (with grant number) or organizations (with url) that supported your study, including funding received from your institution. b. State the initials, alongside each funding source, of each author to receive each grant. c. If any authors received a salary from any of your funders, please state which authors and which funders.*

**Answer:** We further detailed the Financial Disclosure statement as following:

***Funding:*** *This work is conducted by the Stavros Niarchos Foundation (SNF) Global Center at the Child Mind Institute with funding support from the Stavros Niarchos Foundation (SNF) as part of its Global Health Initiative (GHI). [https://www.snfghi.org/]. Grant funding partially supported the salaries of the research team (LEM, JAV, ACC, CBC, GAS, ZM) and covered essential research expenditures, including software, database access, and publication fees. The funder had no role in the methodology, execution, analyses, or interpretation of the data.*

*3.Please provide separate figure files in .tif or .eps format.*

**Answer:** We now provide all figures in .Tif formats.

*4. Your manuscript is missing the following sections: Introduction.*

**Answer:** We have renamed the subheading from "Background" to "Introduction" to address this issue.

*5. We have noticed that you have uploaded Supporting Information files, but you have not included a list of legends. Please add a full list of legends for your Supporting Information files after the references list.*

**Answer:** We have included the following list of legends after the references lists:

**S1 Appendix. Figure A.** PRISMA flowchart

**S1 Appendix. Table A.** PRISMA checklist

**S1 Appendix. Table B.** Search strategy and query per database

**S1 Appendix. Table C.** Prevalence studies: extracted information for each estimate

**S1 Appendix. Table D.** Data extracted for each instrument reported at each study

**S1 Appendix. Table E.** Instruments: evaluation of psychometric properties and language

**S1 Appendix. Table F.** Intervention studies: extracted information

**S1 Appendix. Table G.** Synthesis of each instrument property: coding criteria

**S1 Appendix. Table H.** Reasons for exclusion

**S1 Appendix. Table I.** Mental-health related findings across epidemiological, clinical, and administrative registers

**S1 Appendix. Table J.** Instruments that were applied in Mozambique (without adaptation or validation)

**S1 Appendix.** Reference list for supporting material

**S2 Dataset. Screening documentation.** Original search files and report of all screening decisions.

**S3 Dataset. Extraction sheets.** All included studies, extracted data and risk of bias assessment.

*6. As required by our policy on Data Availability, please ensure your manuscript or supplementary information includes the following: * A numbered table of all studies identified in the literature search, including those that were excluded from the analyses. For every excluded study, the table should list the reason(s) for exclusion. * A table of all data extracted from the primary research sources for the systematic review and/or meta-analysis. The table must include the following information for each study: Name of data extractors and date of data extraction. Confirmation that the study was eligible to be included in the review. All data extracted from each study for the reported systematic review and/or meta-analysis that would be needed to replicate your analyses.* ******* *If applicable for your analysis, a table showing the completed risk of bias and quality/certainty assessments for each study or outcome. Please ensure this is provided for each domain or parameter assessed. For example, if you used the Cochrane risk-of-bias tool for randomized trials, provide answers to each of the signalling questions for each study. If you used GRADE to assess certainty of evidence, provide judgements about each of the quality of evidence factor. This should be provided for each outcome. * An explanation of how missing data were handled.*

*This information can be included in the main text, supplementary information, or relevant data repository. Please note that providing these underlying data is a requirement for publication in this journal, and if these data are not provided your manuscript might be rejected.*

**Answer:** We have included the full datasets in the following files:

- S2 Dataset - Screening. Original search files and report of all screening decisions.
- S3 Dataset - Extraction. All included studies, extracted data and risk of bias assessment.

*7. We note that your Data Availability Statement is currently as follows: "All data relevant to the study are included in the article or uploaded as online supplementary information. No additional data are available." Please confirm at this time whether or not your submission contains all raw data required to replicate the results of your study. Authors must share the “minimal data set” for their submission.*

**Answer:** We have included the full datasets in supporting material. We have improved the Data Availability Statement as following:

"The datasets generated and analyzed during the current study are included in this published article and its supplementary information files (S2 Dataset and S3 Dataset).

We confirm this submission contains all raw data required to replicate the results of the study.

## REVIEWER #1

*Thank you for the opportunity to review this manuscript. This is a highly timely and valuable piece of work for global public health, specifically within child and adolescent mental health.
The authors have done a great job pulling together and synthesizing the scattered evidence on youth mental health in Mozambique. By mapping out prevalence data, existing assessment tools, and current interventions, this paper fills a major knowledge gap. It has real potential to serve as a practical guide for policymakers, researchers, and development partners looking to strengthen mental health infrastructure in the region.
Key Strengths
1.Comprehensive Scope: The decision to track prevalence, tools, and interventions all in one place provides a remarkably thorough overview.
2.Rigorous Approach: The systematic review methodology is solid and aligns well with standard reporting guidelines.
3.Critical Lens: The discussion doesn't just list data; it does a great job highlighting where the current research and service delivery fall short.
4.Cultural Context: The focus on culturally adapted assessment tools is excellent and highly relevant for low-resource settings where Western tools often miss the mark.
5.Actionable Next Steps: The recommendations for future research and capacity-building are practical and forward-looking.

Suggestions for Improvement
1.Policy Integration: It would be great to see a brief discussion on exactly how these findings can plug into Mozambique’s current national mental health policy and service planning. Connecting the data directly to local policy frameworks would make the paper even more impactful.*

**Answer:** We have expanded the discussion section to further connect data to local policy frameworks. Below, we reproduce some text extracts addressing this topic:

[Discussion]

“This highlights the need to adhere to culturally-sensitive practices in the country, including translating tools to local dialects; to this end, it is necessary to prioritize locally trained specialists over the current reliance on international experts (100)”

“There are no studies on ASD, eating disorders, or self-harm, and no assessment tools are available for research or clinical practice on these conditions. This reflects the early stage of service development, with fewer than 5% of people experiencing mental health conditions receiving any form of care (100).”

“It is worth noting that Mozambique has made progress in strengthening child protection mechanisms, with special consideration to gender-based violence and sexual exploitation [(3,66)](https://paperpile.com/c/Z8hP6m/x0pA+5krx). The National Mental Health Strategy and Action Plan (2016–2026) also emphasizes interinstitutional cooperation with child protection bodies as a priority area [(99)](https://paperpile.com/c/Z8hP6m/l4ciH).”

“This reflects a scarcity of postgraduate programs and fragile academic infrastructure. Mozambique has only five medical schools, and no domestic capacity for postgraduate psychiatric training existed until 2018 (the first three Mozambican psychiatrists trained abroad graduated in 2004–2005) [(100)](https://paperpile.com/c/Z8hP6m/6ghH). The need to strengthen national research capacity is acknowledged in the Strategy and Action Plan for Mental Health 2016–2026, which outlines an action front for developing research and human resources [(4)](https://paperpile.com/c/Z8hP6m/AdLYe). The Department of Mental Health has established partnerships with institutions in Portugal, Brazil, and the United States to support postgraduate training in mental health [(101,102)](https://paperpile.com/c/Z8hP6m/4LXxd+SSTcg). As a result, [...].”

*2.Nuance on Prevalence Data: I suggest pushing the limitations of the current prevalence studies a bit further. It’s worth explicitly reminding the reader how few of these studies are actually nationally representative or based on structured diagnostic interviews.*

**Answer:** We further specified these limitations both in results and discussion. In the latter, we specifically included the following debate:

[Discussion] “There are no nationally representative estimates of mental disorders based on standardized diagnostic criteria. Existing data are limited to a small number of disorders assessed in two localized samples from Maputo and Nampula, leaving other regions and rural areas unrepresented. No prevalence estimates are reported for ASD or eating disorders, and no assessment tools are available for research or clinical practice on these conditions.”

*3.Quality / Risk of Bias: While the methodology is clear, adding a quick summary of the risk of bias findings directly into the Results section would help readers immediately grasp the weight and reliability of the evidence being presented.*

**Answer:** We have now added an additional column to Table 1 to present the risk of bias (RoB) for prevalence studies reporting mental health estimates. Similarly, Table 4 includes a column reporting the RoB for each intervention study. Unfortunately, due to the highly heterogeneous scope and methodologies of the prevalence reports on other outcomes, we could not provide a uniform brief summary for all of them. Instead, we extensively detail the methodological data supporting such assessments (such as the use of probabilistic versus non-probabilistic sampling and the validity of assessment scores) both in the text and within the tables. For instrument studies, psychometric validation is fully reported utilizing COSMIN grading codes. We have also included the complete risk of bias assessment sheets in the S3 Dataset.

*4.Elevating the GBV Discussion: The intersection of gender-based violence (GBV) and mental health is a critical point in this paper. Given the high prevalence rates reported, this section could be expanded slightly to dive deeper into the specific mental health trajectories for survivors.*

**Answer:** While we strongly agree with the importance of this intersection, we were unable to locate data on the prospective mental health trajectories of survivors within Mozambique. Instead, we have enriched this section by further connecting the issue to existing national policy frameworks:

[Discussion] “It is worth noting that Mozambique has made progress in strengthening child protection mechanisms, with special consideration to gender-based violence and sexual exploitation.[^6,106^](https://paperpile.com/c/WuM7S4/zuQSN+dVOAY) The National Mental Health Strategy and Action Plan (2016–2026) also emphasizes interinstitutional cooperation with child protection bodies as a priority area.[^1^](https://paperpile.com/c/WuM7S4/72ABd)”

*5.Copyediting: A final, quick proofread would help smooth out a few awkward sentences and ensure that percentages and key technical terms are formatted consistently throughout the text.*

**Answer:** The manuscript has been fully proofread for clarity and consistency, with all copyediting changes highlighted in red throughout the text.

## REVIEWER #2

*This paper presents highly ambitious and culturally tailored systemic review protocol, which involved mapping an entire country's mental health literature across three distinct methodological dimensions (epidemiological prevalence, psychometric validation, and clinical interventions) is a massive undertaking.
The title: It reads as commentary; a direct title would be more appropriate to look like this…“child and adolescent mental health in Mozambique; a systemic review of prevalence, measurement tools and interventions” … so that it is searchable and aligned with indexing.*

**Answer:** We have revised the title to incorporate these specific details. However, we opted to leave the first section unaltered to maintain consistency with similar national studies conducted in other countries..

[Title] “The science of child and adolescent mental health in Mozambique: a systematic review of prevalence estimates, measurement tools, and interventions”

*1. Background:
a. As in most LMICs, the scarcity of ‘some’ studies makes systemic reviews impossible and this is one of such cases. Statements about the “near absence of nationally representative data” and limited research are important but should be balanced by acknowledging existing regional surveys and emerging research efforts. The distinction between limited evidence and absence of evidence should be maintained.*

**Answer:** Thanks for the kind comment.

*b. The review does not provide nationwide epidemiological prevalence estimates; diagnostic evidence is also limited, hence the lack of nationwide representation. Hence, calling this a “nationwide” review overstates its representativeness.*

**Answer:** We have removed the word "nationwide" from the title to prevent any misinterpretation. However, we clarify within the text that the review maintains a national scope in its methodology (i.e., mapping all available studies conducted in Mozambique), thus including studies with regional samples.

*c. The rationale for reviewing prevalence assessment instruments and interventions should be explained earlier. Currently, objectives appears near the end but the reason for integrating these three evidence streams is not fully developed.*

**Answer:** We have introduced this rationale earlier in the manuscript. The introduction section now contextualizes the scope of review by making reference to other national-level compilations:

[Introduction] “In non-Western contexts, synthesizing and appraising **extant** research is essential to enhance the reach and availability of **evidence-based resources such as prevalence rates, assessment instruments, or intervention programs**. National-level compilations on child and adolescent mental health have been conducted in countries such as Greece, Brazil, and South Africa, providing valuable **tools** to inform practice, policy, and research [(9–11)](https://paperpile.com/c/hQSfjA/tCohf+HEVlU+7dthH). This study aims to synthesize the scientific literature on child and adolescent mental health in Mozambique. We conducted a systematic review of prevalence estimates, assessment instruments, and interventions addressing mental health–related outcomes in this population. Our goal is to present a comprehensive overview of evidence-based resources available in the country and to take practical insights for public health.”

*2. Methodology
Overall methodological recommendation: The methodology is generally appropriate and ambitious, but revisions are needed to improve transparency, reduce reviewer bias, and strengthen reproducibility.
• Rigorous Arm-Specific Frameworks: Rather than trying to force one quality appraisal tool onto three very different study designs,you appropriately decoupled your analysis using:
-COSMIN for psychometric evaluation.
-Cochrane RoB 2 and JBI for interventions.
-Using a dedicated prevalence appraisal tool.
• Review design and protocol: The use of PRISMA, PROSPERO registration, COSMIN, and Cochrane/JBI approaches strengthens methodological rigor. However, the rationale for combining prevalence, psychometric validation, and intervention studies into a single systematic review should be clarified.*

**Answer:** We appreciate the reviewer's positive remarks regarding our methodology. We have now elaborated on the rationale for combining these evidence domains (see previous point). Furthermore, the full data extraction sheets and risk of bias assessments have been provided as supplementary material.

*• Appropriate Inclusion Boundaries: Broadly defining mental health outcomes (incorporating life adversities, bullying, and neurodevelopment) while strictly limiting the age to an average of 19 years ensures the dataset will be highly reflective of pediatric and adolescent realities in Mozambique.*

**Answer:** We thank the reviewer for the positive comment.

*1. Search strategy: The search appears comprehensive, including international databases, African sources, institutional repositories, and local journals.
• However, the full, reproducible search strategies for each database should be provided, including the search dates and the number of records retrieved.*

**Answer:** We have now included the full datasets, contain the search files (references exported from each database) and the complete screening documentation. Figure 1 and Figure A (S1 Appendix) report the search results per database; they have been updated to reflect the most recent search run from August, 2026.. Table B (S1 Appendix) details the specific queries utilized for each database, and has now been expanded with a new column stating the date of each search.

*• Moreover, the use of the “Rayyan” tool was used to “automatically [remove] pairs with an overall similarity index above 95%.” The Epidemiological Impact of this action is that it would automatically delete at a 95% threshold, which is dangerous. It frequently swallows distinct papers that share highly similar titles, such as sequential cohort updates (e.g., "Childhood Trauma in Maputo: Part I" vs. "Part II"), multi-author consortium papers, or different chapters within the same doctoral thesis.
• Best practice dictates that automated deduplication should only occur for 100% exact matches (identical DOIs or identical title/author/year strings). Anything else should be sent to a manual verification queue.*

**Answer:** We thank the reviewer for raising this important methodological point. Our initial description lacked clarity: we did not use a standard "similarity index", but the “Overall Similarity Percentage”, which is a specific metric provided by Rayyan platform. It calculates similarity considering all metadata fields (including titles, authors, dates, database, and DOIs). As references exported from different databases inherently contain formatting variations, a 100% similarity score is exceedingly rare, and even highly similar titles would not result in exact matches.

We make reference to a review assessing the performance of this metric (doi:[10.2196/94317](https://doi.org/10.2196/94317)): *“Higher thresholds of ≥95% approximate exact matching with tolerance for minor variation and carry a lower risk of false positives. Lower thresholds increase the number of duplicates detected but require more careful manual review of flagged records. We recommend beginning at ≥95% and reviewing the results before considering a lower threshold.”.*

The manuscript has been revised to more clearly detail deduplication procedures:

[Methods] “The review was managed using Rayyan software. Potential duplicates were detected, automatically removing pairs with a 100% match in title/DOI or Overall Similarity Percentage above 95% (a metric calculated based on all characters and metadata associated with the reference); remaining candidates were manually verified. This was informed by an assessment of the deduplication tool, which suggested that an initial 95% cut-off carries a negligible risk of false exclusions **(17).”**

*2. Study selection: The use of independent dual screening at title/abstract level is appropriate, but the single-reviewer screening protocol is a concerning “high risk of selection bias” at the crucial stage of the screening process. • You state that after title/abstract screening, “a single reviewer performed full-text screening.” Furthermore, for Google Scholar, regional databases, and local repositories, “regional and local data sources were also screened by a single author.” • While standard systematic review guidelines (PRISMA, Cochrane) strictly recommend two independent reviewers for both title/abstract and full-text screening. • Relying on a single reviewer, even with a 20% random verification check, significantly increases the risk of missing eligible studies (false negatives), especially when navigating local grey literature or diverse Portuguese thesis repositories.*

**Answer:** We have expanded upon the rationale for this procedure. Drawing on previous national systematic reviews aimed at compiling evidence, this strategy was designed to prioritize inclusion sensitivity over specificity (i.e., when in doubt, studies were included to make the review as comprehensive as possible). This choice is supported by empirical evaluations discussed in the Cochrane manuals. We clarify this in the following quote:

[Methods] “When adopting single-reviewer screening procedures, 20% of the records were randomly verified by a second reviewer (LEM); this author also resolved discrepancies arising from two author independent screening. This strategy draws on the national systematic review conducted in Brazil, which utilized a single-reviewer approach emphasizing sensitivity over specificity to maximize article inclusion [(10)](https://paperpile.com/c/Z8hP6m/Dq76). This approach is particularly well-suited to compilation reviews and has demonstrated reliable performance in empirical evaluations informing Cochrane guidance [(14–16)](https://paperpile.com/c/Z8hP6m/Ihju+s0ov+XM1n)**.”**

*3. Eligibility criteria: Inclusion criteria are broad and cover multiple mental health domains; however, the scope should be better structured using a framework such as PCC/PICO to improve clarity and reproducibility.*

**Answer:** We could not strictly apply the traditional PCC or PICO frameworks, as they were not suitable for the comprehensive scope of this review, which encompasses a broad range of methodological areas and outcomes. Instead, we have provided a clearer structure for the review's scope, modeled after similar national reviews conducted in Brazil, Greece, and South Africa. The diagnostic domains were initially based on the DSM and subsequently expanded drawing on the literature mapping from those aforementioned national reviews. The decision to focus on prevalence, assessment instruments, and interventions was made to generate a highly informative evidence base for both clinical practice and future research. This rationale is now reflected in the manuscript as follows:

[Methods] "This scope builds upon comprehensive cataloguing efforts in Brazil, Greece, and South Africa [(9–11)](https://paperpile.com/c/hQSfjA/tCohf+HEVlU+7dthH). It was initially designed to encompass all DSM-5 diagnostic domains and was subsequently expanded to incorporate additional areas identified through mapping of the national literature corpus.”

[Introduction] “In non-Western contexts, synthesizing and appraising **extant** research is essential to enhance the reach and availability of **evidence-based resources such as prevalence rates, assessment instruments, or intervention programs**. National-level compilations on child and adolescent mental health have been conducted in countries such as Greece, Brazil, and South Africa, providing valuable **tools** to inform practice, policy, and research [(9–11)](https://paperpile.com/c/Z8hP6m/kJsJW+Dq76+HSjoU). This study aims to synthesize the scientific literature on child and adolescent mental health in Mozambique. We conducted a systematic review of prevalence estimates, assessment instruments, and interventions addressing mental health–related outcomes in this population. Our goal is to present a comprehensive overview of evidence-based resources available in the country and to take practical insights for public health.”

*4. Risk of bias assessment: Although appropriate tools are mentioned, the manuscript should clearly report which tools were applied to each study category and how risk-of-bias judgments influenced interpretation of findings.*

**Answer:** This concern was also expressed by Reviewer #1. We copy our response addressing this point: “We have now added an additional column to Table 1 to present the risk of bias (RoB) for prevalence studies reporting mental health estimates. Similarly, Table 4 includes a column reporting the RoB for each intervention study. Unfortunately, due to the highly heterogeneous scope and methodologies of the prevalence reports on other outcomes, we could not provide a uniform brief summary for all of them. Instead, we extensively detail the methodological data supporting such assessments (such as the use of probabilistic versus non-probabilistic sampling and the validity of assessment scores) both in the text and within the tables. For instrument studies, psychometric validation is fully reported utilizing COSMIN grading codes. We have also included the complete risk of bias assessment sheets in the S3 Dataset."

Moreover, we have now clearly indicated which RoB tool was used in intervention studies presented in Table 4 (Cochrane RoB 2 for the single RCT; and the Joanna Briggs Institute (JBI) checklist for non-randomized designs for the remaining trials).

We have also indicated quality concerns when reporting findings:

[Results] “National data points on mental health disorders were limited to screening cut-off scores reported at the 2022-2023 Mozambique Demographic and Health Survey [(55,61)](https://paperpile.com/c/Z8hP6m/AQfJ+AuWO). This study involved 4548 participants aged 15 - 19 years-old completing the Generalized Anxiety Disorder 7-item (GAD-7) and the Patient Health Questionnaire 9-item (PHQ-9). It was estimated that 6·3% to 18·51% presented significant symptoms of generalized anxiety disorders (GAD-7 ≥ 5 or ≥ 7), whilst 6,6% to 10,25% experienced significant major depressive disorder symptoms (PHQ-9 ≥ 8 or ≥ 10). Although the Disrupting Harm project utilized a nationally representative survey of Mozambican adolescents incorporating structured scales, prevalence estimates relied on single-item responses rather than validated cut-off scores [(66)](https://paperpile.com/c/Z8hP6m/x0pA).”

[Results] “The RCT assessed a World Bank program among 1,907 preschool children in high-poverty areas [(30)](https://paperpile.com/c/Z8hP6m/qCW5). It demonstrated significant developmental gains with an implementation cost of approximately $3 per child per month. However, it was judged to have a high risk of bias across all assessed domains, including selection bias, blinding of participants and outcome assessors, outcome measurement, and selective outcome reporting.”

*5. Data synthesis: Given the substantial heterogeneity of outcomes, populations, and instruments, the narrative synthesis approach is reasonable. However, the methods should describe more explicitly how studies were grouped and how conflicting evidence was handled.*

**Answer:** We have added a “Data Synthesis and Presentation” subsection to the Methods (reproduced below). Because the available literature was scattered and seldom yielded multiple studies on the exact same subject, there was no conflicting evidence to address.

[Methods]

“Data synthesis & presentation

We followed previous national compilation efforts to synthesize all available evidence in an accessible format, aiming at presenting a "catalogue of evidence" that was informative, comprehensive, and able to account for the substantial heterogeneity across populations, study methodologies, and outcomes [(9–11)](https://paperpile.com/c/Z8hP6m/kJsJW+Dq76+HSjoU).

Data were presented for each major area (prevalence, instruments, and interventions) and organized according to diagnostic domains. These domains included mental health disorders, mental health symptoms, and mental health-related outcomes (such as substance use, psychosocial adversities, and quality of life).

For prevalence, data was presented for each study (aggregating findings across multiple reports and secondary analyses referring to the same parent dataset). To align with the diagnostic domains, studies were initially grouped by methodological design: clinical estimates utilizing diagnostic interviews (reporting mental health disorders), estimates using mental health screening scores (reporting mental health symptoms), and findings from unstructured questionnaires and other surveys (reporting mental health-related outcomes). Within each of these categories, studies were sequentially sorted by sample size.

For assessment instruments, data was presented by each tool. The psychometric classification per instrument was summarized by aggregating data across studies according to the codes presented in Table G in S1 Appendix (with all supporting data detailed in the footnotes).

For interventions, data were presented for each trial (encompassing findings reported across one or multiple publications). Due to the limited number of included studies, all data could be synthesized within a single table. We opted to order the studies by sample size rather than by diagnostic category.”

*Assessment instruments: Application of COSMIN is a methodological strength, but reporting should include clearer details on quality ratings, domains assessed, and certainty of evidence for each instrument.*

**Answer:** We have now included the full extraction sheets (including quality grading) in S3 Dataset. When presenting data for each instrument, we now detail the associated parameters grounding psychometric rating:

[Table 3] **“Abbreviations**: cross-cultural validity (CC), criterion validity (CR), construct validity (CV), internal consistency (IC), interrater reliability (IR), measurement error (ME), post-traumatic stress disorder (PTSD), responsiveness (R), structural validity (SV), test-retest reliability (TR). “+” stands for a property with positive evidence for validation, “+/-” for mixed evidence, “-” for negative evidence, “?” for available evidence that cannot be classified as positive or negative, “NA” for non-available evidence (see S1 Table B in S1 Appendix*).*

**Notes:** **[MINI-KID]** **^a^**Correlations with SDQ (Area Under the Curve: 0·7 - 0·9; Sensitivity: 0·72 - 0·83; Specificity: 0·67 - 0·74), with GAD-7 (AUC 0·85; Sensitivity 0·78; Specificity 0·72) and with PHQ-A (AUC: 0·84; Sensitivity: 0·81; Specificity: 0·72); **[KSADS-PL] ^b^**There is no multigroup factorial analysis as per COSMIN criteria for cross-cultural validity, but authors debate a predictive performance and comorbidity pattern similar to the one found in studies worldwide; **[GAD-7] ^c^**Correlation Mini-KID (AUC: 0·85; Sensitivity: 0·78; Specificity: 0·72); **^d^**Correlation index > 0·83 with WHO-5 well-being; **^e^**Cronbach Alpha: 0·69 - 0·84; **^f^**Intra-Class Correlation (ICC): 0·7 (one-week retest); **[PHQ-A] ^g^**Correlation with Mini-KID (AUC: 0·84; Sensitivity: 0·81; Specificity: 0·72); **^h^**Cronbach Alpha: 0·8; **^i^**ICC: 0·74 (one-week retest); **[SDQ] ^j^**Correlation with MINI-KID (AUC: 0·7 - 0·9; Sensitivity: 0·72 - 0·83; Specificity: 0·67 - 0·74); **^k^**Cronbach Alpha: 0·73; **^l^**ICC: 0·77 (one-week retest); **[CSHQ] ^m^**Correlation with Parent`s Report Form (AUC: not reported; Sensitivity: 0·89; Specificity: 0·71); **^n^**Cronbach Alpha: 0·75; **[SNAP-IV] ^o^**Authors reported individual items were distributed differently than in other countries, as well as poor overall psychometric performance; **^p^**Correlations with KSADS-PL clinical interview following DSM-5 criteria (AUCs < 0·53); **^q^**Correlations between individual symptoms rated by teachers and parents: kappa (k) scores < 0·35; **[CBCL]** **^r^**The scale was also applied in Argentina, India, Kenya, and South Africa, but only in Mozambique it was unable to detect clinical cases, which authors attributed to poor cultural fit; **[BDI-II] ^s^**Cronbach alpha: 0·97; **[NITE] ^t^**Cronbach alpha: 0·94; **[SRQ-20] ^u^**Cronbach alpha: 0·93; **[FAMELO] ^v^**Cronbach Alpha: 0·74 to 0·93; **^w^**RMSEA: 0·06 and CFI: 0·92; **[RSES] ^x^**ΔR2: 0·39 (between self-esteem and a model involving relatedness, competence, and autonomy); **^y^**Cronbach Alpha: 0·73; **^z^**Confirmatory Factor Analysis (CFA) for 2-factor model (CFI: 1; RMSEA: 0·000; TLI: 1; SRMR: 0·011); **[WHO-5] ^aa^**Correlation with GAD-7 (0·83); **^bb^**Cronbach Alpha: 0·79; **[ICDP]** **^cc^**Statistically significant differences between pre- and post-intervention assessment.”

*7. Intervention evidence: The inclusion of RCTs and non-randomized studies is appropriate; however, conclusions regarding intervention effectiveness should be cautious because most studies had high risk of bias.*

**Answer:** To address this, we excluded the column synthesizing efficacy as + or - (Table 4). Instead, we present key findings in a dedicated text column. We revised the manuscript section reporting intervention findings to more clearly indicate RoB concerns:

[Results] “The RCT assessed a World Bank program among 1,907 preschool children in high-poverty areas [(30)](https://paperpile.com/c/Z8hP6m/qCW5). It demonstrated significant developmental gains with an implementation cost of approximately $3 per child per month. However, it was judged to have a high risk of bias across all assessed domains, including selection bias, blinding of participants and outcome assessors, outcome measurement, and selective outcome reporting.”

*8. Reproducibility: Additional supplementary material should include the screening process, excluded full-text studies with reasons, extraction templates, and complete risk-of-bias tables to enhance transparency.*

**Answer:** We have now included the full datasets as supporting material. S2 Dataset documents all screening records and decisions. S3 Dataset presents all extraction sheets, including risk-of-bias tables.

*“Results:
1. Organization and clarity: The results are generally well structured, covering prevalence, assessment instruments, and interventions. However, clearer separation between study characteristics, risk-of-bias findings, and main outcomes would improve readability.”*

**Answer:** Thanks for the positive appraisal. We have now added a section in the methods outlining the rationale and structure for data synthesis and presentation. We have also improved the presentation of findings across manuscript text.

*“2. Quantitative reporting: The review provides the number of included studies and key findings (58 prevalence reports, 35 instruments, and 7 intervention studies), it could benefit from more detailed quantitative summaries, including sample sizes, geographic distribution, study designs, and outcome estimates.”*

**Answer:** We have now added a section in the methods outlining the rationale and structure for data synthesis and presentation. Data is presented per area (prevalence, instrument, intervention) and, within each area, grouped per diagnostic domain and sorted according to methodological characteristics. For detailed data, we have now included the full dataset in supporting material.

*3. Prevalence findings: Reported prevalence estimates are important but should be interpreted cautiously because many findings are based on localized samples rather than nationally representative populations. Was there any an attempt made to conduct a meta-analysis to estimate a pooled prevalence of specific conditions, if data allowed?*

**Answer:** We have now more clearly indicated limitations regarding prevalence estimates, considering both data availability and methodological quality. Moreover, the discussion section debates reasons precluding meta-analysis.

[Discussion] “There are no nationally representative estimates of mental disorders based on standardized diagnostic criteria. Existing data are limited to a small number of disorders assessed in two localized samples from Maputo and Nampula, leaving other regions and rural areas unrepresented. No prevalence estimates are reported for ASD or eating disorders, and no assessment tools are available for research or clinical practice on these conditions.”

[Discussion] “Meta-analysis was not feasible due to the small number and significant heterogeneity of the available studies (i.e., there were no two estimates for the same prevalence outcome or instrument property within a similar population or study design to allow for data aggregation).”

*“4. Assessment instruments: The results provide useful information on cross-cultural adaptation and psychometric performance. However, presenting a summary table comparing instruments, validation properties, and quality ratings would strengthen the findings.”*

**Answer:** Table 3 summarizes the psychometric properties of all instruments based on COSMIN codes, allowing for some comparison between tools. However, robust head-to-head comparisons were limited, as available literature rarely reported more than one instrument per outcome.

*“5. Intervention evidence: The limited number of intervention studies and high risk of bias are appropriately highlighted. The results should avoid implying effectiveness beyond what the available evidence supports.”*

**Answer:** We have previously addressed this in point 7.

*6. Missing evidence synthesis: A clearer description of evidence gaps (e.g., disorders not studied, regions lacking data, age groups underrepresented) would improve the contribution of the results.*

**Answer:** We tried to represent an evidence map in Figure 2 using a heatmap. However, we could not summarize all literature gaps within a single representation. We then debated data gaps across text, as in the the following extracts:

[Discussion] “There are no nationally representative estimates of mental disorders based on standardized diagnostic criteria. Existing data are limited to a small number of disorders assessed in two localized samples from Maputo and Nampula, leaving other regions and rural areas unrepresented. No prevalence estimates are reported for ASD or eating disorders, and no assessment tools are available for research or clinical practice on these conditions.”

[Discussion] “National-level data with adolescent samples indicate alarming rates of exposure to adversities and emotional and behavioral symptoms. In particular, the high prevalence of physical, emotional, and sexual violence stands out as a clear public health priority. These experiences are not only key social determinants of psychological distress and mental disorders, but also undermine healthy development and long-term prospects, potentially perpetuating cycles of violence and trauma across future generations [(86–89)](https://paperpile.com/c/hQSfjA/nAT3w+Xol3I+xCt4R+aOoHp). While current data in Mozambique is restricted to adolescents, it is likely that such exposures begin at younger age groups. Surveys including infants, preschoolers, and school-aged children are recommended to support data-driven action from the earliest possible stages of development [(87)](https://paperpile.com/c/hQSfjA/Xol3I).”

*“Discussion 1. The discussion appropriately highlights major evidence gaps and the need for improved mental health research capacity. However, some statements should be more cautious given the limited representativeness of available studies.”*

**Answer:** As shown in previous quotes, we have toned down some statements to more cautiously reflect limitations in available evidence.

*“2. The comparison suggesting higher prevalence of mental disorders than global estimates should be interpreted carefully due to differences in sampling methods, diagnostic tools, and study settings.”*

**Answer:** We have now clarified that the comparison relied on similar methodologies, but was inherently limited by the sparse sample representation in the Mozambican literature.

[Discussion] “Although limited to two urban samples comprising a total of 1,234 participants, clinical data from Mozambique indicate a higher prevalence of mental health disorders compared to global estimates assessed with similar manualized criteria [(10,83)](https://paperpile.com/c/hQSfjA/mnFnL+HEVlU).”

*3. The discussion effectively links findings to mental health system strengthening, workforce development, and research priorities. This is a major strength.*

**Answer:** We appreciate this positive remark.

*4. Limitations are discussed, including missed grey literature and heterogeneity. However, the impact of single-reviewer screening and potential publication bias should be emphasized further.*

**Answer:** We now expanded upon this point:

[Limitations] “Although screening was often conducted by a single reviewer, the emphasis on maximizing inclusion and identifying as many relevant studies as possible helped reduce the risk of missing important evidence. However, this review does not capture the totality of mental health research on children and adolescents in Mozambique. Additional data may be scattered across uncaptured sources, including multinational assessments reporting only aggregate results, non-academic sources, and conference abstracts [(93)](https://paperpile.com/c/hQSfjA/5YXzu). Given the limited scope of local research, these sources could represent a substantial body of evidence.”

*5. The conclusion should distinguish between the lack of evidence and evidence of absence. For example, the absence of studies on certain disorders (e.g., ASD, eating disorders, self-harm) reflects research gaps rather than absence of these conditions.*

**Answer:** We agree with this comment. As shown in previous points, we have more clearly indicated gaps concerning data availability, while also clarifying methodological limitations of available research.

*Overall assessment: The results and discussion provide a valuable overview of evidence on child and adolescent mental health in Mozambique, but the interpretation should be more conservative and better aligned with the limitations of the available evidence.*

**Answer:** Thanks for this careful appraisal. As previously highlighted, the interpretation was revised to reflect the limitations of available data.

## REVIEWER #3

*This abstract entitled "The science of child and adolescent mental health in Mozambique: a nationwide systematic review of prevalence rates, assessment instruments, and interventions" presents a systematic review of child and adolescent mental health research in Mozambique. The study synthesizes evidence on prevalence, assessment instruments, and interventions among individuals aged 0–19 years, addressing an important gap in mental health knowledge within a low-resource setting.
Some strengths observed, leading to the above conclusions in my review are:
i. The study addresses an important Public Health gap by focusing on a largely understudied population. Mozambique has a large youth population, yet limited mental health research exists. By compiling available evidence, the review provides valuable information for policymakers, clinicians, and researchers.
ii. The Methodology utilized was comprehensive in that the authors searched multiple international and regional databases, including PubMed, Web of Science, PsycINFO, CINAHL, Google Scholar, African Index Medicus, and local catalogues. This broad search strategy increased the likelihood of identifying relevant studies and minimized publication bias.
iii. Use of Established Guidelines- Data was extracted using recognized methodological standards like COSMIN for measurement instruments and Cochrane guidance for systematic reviews, enhancing the credibility and transparency of the review.
iv. Identification of Major Mental Health Concerns- the findings reveal significant rates of anxiety, depression, ADHD, violence exposure, and suicide among Mozambican youth. These results highlight an urgent need for mental health services and preventive interventions.
v. The review not only examined prevalence but also assessed the quality of screening and diagnostic tools. Identifying culturally adapted instruments such as the MINI-KID, GAD-7, PHQ-A, and SDQ was particularly useful for future research and clinical practice. The fact that assessment instruments were evaluated gives the study more credibility.

The study also had some limitations which are outlined below: these formed the basis for the response “No” to question 2.
• It was noted that only four instruments were properly adapted and psychometrically validated. Reliance on unadapted tools could potentially affect the accuracy and validity of prevalence estimates reported in some studies.
• Although methodological frameworks were used, there is lack of detailed information regarding risk of bias, heterogeneity, or the quality of included studies. These factors are a very important consideration for interpreting the strength of evidence availed.
• Inadequate Geographic spread: The prevalence studies were conducted in Nampula and Maputo. Mozambique is geographically and culturally diverse, and findings from these regions may not reflect mental health conditions in other provinces.*

*• Scarcity of Intervention Studies
The review identified only seven trials. This indicates a significant lack of evidence regarding effective mental health interventions for children and adolescents in Mozambique.
• Only two studies provided clinical prevalence estimates for mental disorders. Consequently, the reported prevalence rates may not have accurately represented the entire country. This relatively low prevalence consequently also limits generalizability.*

*The review has major significance and implications. It demonstrates that Mozambican children and adolescents experience substantial mental health challenges alongside high levels of violence and psychosocial adversity. At the same time, there is a shortage of validated assessment tools and evidence-based interventions. The findings suggest the need for:
• Nationally representative epidemiological studies.
• Cultural adaptation and validation of mental health assessment instruments.
• Development and testing of evidence-based interventions.
• Integration of mental health services into schools and primary healthcare settings.
• Increased investment in youth mental health research and policy.

On review of the conclusion of this study, I found it to be consistent with the results presented. The evidence indicates substantial exposure to violence, abuse, and other psychosocial stressors among children and adolescents in Mozambique. The reported prevalence of anxiety, depression, and ADHD supports the assertion of a considerable mental health burden.
The review acknowledges evidence limitations- the phrase "according to limited clinical data" appropriately recognizes that prevalence estimates are based on only a small number of clinical studies. This demonstrates caution and avoids overstating the findings.
The conclusion correctly identifies the shortage of validated mental health assessment instruments which is a key research gap. However, since only a few tools were culturally adapted and psychometrically tested, this is an important limitation affecting both research and clinical practice.

The recommendation for culturally sensitive approaches is particularly important in mental health research. Assessment tools and interventions developed in high-income countries may not adequately capture local experiences, languages, and expressions of psychological distress.*

**Answer:** We thank the reviewer for this careful appraisal and positive comments on your work.

*It however, also had some limitations including:*

*i. Lack of specific recommendations:
While the conclusion calls for culturally sensitive research approaches, it does not explicitly recommend actions such as: Expanding epidemiological studies, developing intervention programs, training mental health professionals and integrating mental health services into schools and primary healthcare. Including these recommendations would strengthen the practical implications of the review.*

**Answer:** We have included the following specific recommendation:

[Discussion] “This highlights the need to adhere to culturally-sensitive practices in the country, including translating tools to local dialects; to this end, it is necessary to prioritize locally trained specialists over the current reliance on international experts (100)".

We also had previously debated other practical implications regarding epidemiological studies:

[Discussion] “Moreover, the reliability of epidemiological and civil registration data in Mozambique is limited, with reports that nearly half of children under the age of five are not registered in official records [(3)](https://paperpile.com/c/hQSfjA/9LWLU). Similarly, other register-based studies in Mozambique are scarce and rely primarily on service-level data from isolated facilities, limiting their generalizability. An integrated national health information system that includes mental health outcomes is needed to strengthen epidemiological data and monitoring across the country

While current data in Mozambique is restricted to adolescents, it is likely that such exposures begin at younger age groups. Surveys including infants, preschoolers, and school-aged children are recommended to support data-driven action from the earliest possible stages of development [(87)](https://paperpile.com/c/hQSfjA/Xol3I). “

*ii. General Statement on Mental Disorder Burden
The phrase "high estimated burden" is reasonable, but because national prevalence data are limited, the wording could be interpreted cautiously. A statement such as "available evidence suggests a substantial burden" might more accurately reflect the strength of the evidence.*

**Answer:** We revised this section accordingly:

[Abstract] “Conclusion: Children and adolescents in Mozambique face significant psychosocial adversity, with available evidence suggesting a substantial burden of mental disorders according to limited clinical data.”

*The conclusion is generally concise, balanced, and supported by the review findings. It effectively summarized the major messages which are pointed out below:
1. Mozambican children and adolescents experience significant psychosocial adversity.
2. Mental health disorders appear to represent an important public health concern.
3. There is a shortage of validated assessment tools.
4. More culturally appropriate research is needed.
The conclusion could be improved by providing clearer recommendations for policy, service development, and future intervention research, but it appropriately reflects the available evidence and avoids overstating the findings. The conclusion is therefore consistent with findings, very clear and acknowledges the limitations. However, it does not articulate the practical implications of the results.*

**Answer:** In the previous point, we highlighted some changes made to address practical implications. We also reproduce some quotes connecting findings to local policy frameworks (as detailed in responses to Reviewer #1):

[Discussion]

“This highlights the need to adhere to culturally-sensitive practices in the country, including translating tools to local dialects; to this end, it is necessary to prioritize locally trained specialists over the current reliance on international experts (100)”

“There are no studies on ASD, eating disorders, or self-harm, and no assessment tools are available for research or clinical practice on these conditions. This reflects the early stage of service development, with fewer than 5% of people experiencing mental health conditions receiving any form of care (100).”

“It is worth noting that Mozambique has made progress in strengthening child protection mechanisms, with special consideration to gender-based violence and sexual exploitation [(3,66)](https://paperpile.com/c/Z8hP6m/x0pA+5krx). The National Mental Health Strategy and Action Plan (2016–2026) also emphasizes interinstitutional cooperation with child protection bodies as a priority area [(99)](https://paperpile.com/c/Z8hP6m/l4ciH).”

“This reflects a scarcity of postgraduate programs and fragile academic infrastructure. Mozambique has only five medical schools, and no domestic capacity for postgraduate psychiatric training existed until 2018 (the first three Mozambican psychiatrists trained abroad graduated in 2004–2005) [(100)](https://paperpile.com/c/Z8hP6m/6ghH). The need to strengthen national research capacity is acknowledged in the Strategy and Action Plan for Mental Health 2016–2026, which outlines an action front for developing research and human resources [(4)](https://paperpile.com/c/Z8hP6m/AdLYe). The Department of Mental Health has established partnerships with institutions in Portugal, Brazil, and the United States to support postgraduate training in mental health [(101,102)](https://paperpile.com/c/Z8hP6m/4LXxd+SSTcg). As a result, [...].”

*“Overall, this was a valuable and timely systematic review that provided the most comprehensive overview currently available; of child and adolescent mental health research in Mozambique. Its major contribution lies in highlighting both the burden of mental health problems and the substantial gaps in research infrastructure. However, the limited number of prevalence studies, validated instruments, and intervention trials means that conclusions should be interpreted cautiously. Overall, the review offers strong evidence for prioritizing child and adolescent mental health as a public health and research agenda in Mozambique. It is a very relevant study with great policy and clinical significance. It has fairly strong methodological rigor but relatively low generalizability. Evidence completeness could have been better however the study results remain very significant and valuable with critical evidence to facilitate improved clinical and community interventions.”*

**Answer:** We appreciate the reviewer’s detailed and positive appraisal of our work. We believe we have addressed the main limitations in our preceding responses. Additionally, we have enhanced the completeness of our evidence base by updating the literature searches and providing the full datasets in the supporting information, including screening documentation and data sheets.

## REVIEWER #4

*This manuscript addresses an important and underexplored area by synthesizing the available evidence on child and adolescent mental health in Mozambique. The study is notable for its nationwide scope, its focus on a population and setting where mental health research remains limited, and its effort to compile prevalence estimates, assessment instruments, and interventions in a single review. Furthermore, the review employed a comprehensive search strategy that included grey literature sources, as it improves the completeness of the evidence synthesis and helps mitigate publication bias. By bringing together these different strands of evidence, the manuscript provides a useful overview of the current state of the field and highlights important gaps for future research and service development. However, there are several matters requiring clarification.
Major comments:
1. The inclusion criteria state that the review considered "Our scope was the mental health of children and/or adolescents in Mozambique considering outcomes in a broad sense..." It would be helpful to clarify the rationale for the selection of the mental health outcomes included in the review. For example, whether these outcomes chosen based on disease burden, International Statistical Classification of Diseases, or another prespecified framework. Providing this information would improve the transparency of the review.*

**Answer:** We have now provided a clearer framework for the scope of outcomes, which drew in previous national reviews conducted in Brazil, Greece, and South Africa.

[Methods] "This scope builds upon comprehensive cataloguing efforts in Brazil, Greece, and South Africa [(9–11)](https://paperpile.com/c/hQSfjA/tCohf+HEVlU+7dthH). It was initially designed to encompass all DSM-5 diagnostic domains and was subsequently expanded to incorporate additional areas identified through mapping of the national literature corpus.”

*2. The inclusion criteria state, "We excluded multi-country studies that did not provide specific data for Mozambique, as well as general population studies without specific data for children or adolescents". However, Skar et al. (2014) appears to involve caregivers only, and adolescent-specific data are not clearly reported. The manuscript would benefit from a clearer explanation of how studies primarily targeting caregivers or teachers were handled in the eligibility criteria, particularly in relation to the stated focus on children or adolescents. Please also clarify how Skar et al. (2014) was considered eligible, as the rationale is not sufficiently clear.*

*Reference: Skar, A.-M. S., Sherr, L., Clucas, C., & von Tetzchner, S. (2014). Evaluation of Follow-Up Effects of the International Child Development Programme on Caregivers in Mozambique. Infants & Young Children, 27(2), 120–135.* [*https://doi.org/10.1097/IYC.0000000000000006*](https://doi.org/10.1097/IYC.0000000000000006)

**Answer:** We included studies with outcomes reported for children, including those based on proxy-report measures (e.g.: rated by teachers) or proxy-delivered interventions (e.g.: intervention delivered to caregivers reporting outcomes for children). In Skar (2014), the intervention was delivered to parents but reported outcomes for children (*“Statistically significant decrease on child conduct problems scores")*. We have clarified eligibility criteria in the manuscript (see quote below) and further provided full screening records in S2 Datasets, including documentation of inclusion/exclusion decisions per study..

[Methods] “For eligibility, studies had to report specific data for samples in Mozambique with an average age up to 19-years-old (regardless of outliers), including data collected through self- or proxy-reported measures (for instance, caregiver- or teacher-rated scales measuring children's symptoms).”

*3. The methods section would benefit from a clearer description of the planned synthesis approach. Specifically, please indicate whether a meta-analysis was conducted. If quantitative synthesis was not feasible, please describe the narrative synthesis methods in sufficient detail. Referring to the Synthesis Without Meta-Analysis (SWiM) reporting guideline by Campbell et al. (2020) may help ensure transparent and comprehensive reporting of synthesis without meta-analysis. In addition, please describe how the results were synthesized and presented. Providing this information would improve the transparency and reproducibility of the review and is consistent with the reporting recommendations outlined in the PRISMA guidelines.*

*References:
Campbell, M., McKenzie, J. E., Sowden, A., Katikireddi, S. V., Brennan, S. E., Ellis, S., Hartmann-Boyce, J., Ryan, R., Shepperd, S., Thomas, J., Welch, V., & Thomson, H. (2020). Synthesis without meta-analysis (SWiM) in systematic reviews: Reporting guideline. BMJ, 368, l6890.* [*https://doi.org/10.1136/bmj.l6890*](https://doi.org/10.1136/bmj.l6890)

**Answer:** We have added a new subsection describing the rationale and framework for data synthesis and reporting (see below). While the SWiM checklist provided useful guidance, it was not formally included as a reference tool because it was not entirely suitable for the comprehensive scope of this review (which encompasses a broad range of methodological areas and outcomes).

[Methods]

“Data synthesis & presentation

We followed previous national compilation efforts to synthesize all available evidence in an accessible format, aiming at presenting a "catalogue of evidence" that was informative, comprehensive, and able to account for the substantial heterogeneity across populations, study methodologies, and outcomes [(9–11)](https://paperpile.com/c/Z8hP6m/kJsJW+Dq76+HSjoU).

Data were presented for each major area (prevalence, instruments, and interventions) and organized according to diagnostic domains. These domains included mental health disorders, mental health symptoms, and mental health-related outcomes (such as substance use, psychosocial adversities, and quality of life).

For prevalence, data was presented for each study (aggregating findings across multiple reports and secondary analyses referring to the same parent dataset). To align with the diagnostic domains, studies were initially grouped by methodological design: clinical estimates utilizing diagnostic interviews (reporting mental health disorders), estimates using mental health screening scores (reporting mental health symptoms), and findings from unstructured questionnaires and other surveys (reporting mental health-related outcomes). Within each of these categories, studies were sequentially sorted by sample size.

For assessment instruments, data was presented by each tool. The psychometric classification per instrument was summarized by aggregating data across studies according to the codes presented in Table G in S1 Appendix (with all supporting data detailed in the footnotes).

For interventions, data were presented for each trial (encompassing findings reported across one or multiple publications). Due to the limited number of included studies, all data could be synthesized within a single table. We opted to order the studies by sample size rather than by diagnostic category.”

*4. The manuscript would benefit from clarification regarding whether an assessment of reporting bias or certainty of evidence was considered in the methods section, as recommended on PRISMA checklist item #14 and #15. The authors may find references by Page et al. (2022) and Schünemann et al. (2024) helpful, for guiding the implementation and reporting of these methodological components. In addition, as this is a systematic review, please indicate whether these assessments were performed; if not, a brief rationale would be appreciated, especially considering the heterogeneity of the included evidence (prevalence studies, assessment instruments, and interventions), along with a discussion of how this may affect interpretation of the findings.*

*Page, M. J., Higgins, J. P. T., & Sterne, J. A. (2022). Chapter 13: Assessing risk of bias due to missing evidence in a meta-analysis | Cochrane. In J. P. T. Higgins, J. Thomas, J. Chandler, M. Cumpston, T. Li, & M. J. Page (Eds.), Cochrane Handbook for Systematic Reviews of Interventions. Cochrane.* [*https://www.cochrane.org/authors/handbooks-and-manuals/handbook/current/chapter-13*](https://www.cochrane.org/authors/handbooks-and-manuals/handbook/current/chapter-13)
*Schünemann, H. J., Higgins, J. P. T., Vist, G. E., Glasziou, P., Akl, E. A., Skoetz, N., & Guyatt, G. H. (2024). Chapter 14: Completing ‘Summary of findings’ tables and grading the certainty of the evidence [last updated August 2023]. In J. Higgins, J. Thomas, J. Chandler, M. Cumpston, T. Li, & M. Page (Eds.), Cochrane Handbook for Systematic Reviews of Interventions version 6.5. Cochrane.* [*cochrane.org/handbook*](http://cochrane.org/handbook)*.*

**Answer:** As detailed in our previous responses, we have expanded the Results section to present the RoB in greater detail, while the Discussion appraises the methodological strengths and weaknesses of the supporting evidence for each finding. We have also included a summary of the RoB in Tables 1 and 4, and the complete RoB assessment sheets are fully available in the S2 Dataset. However, because the literature was highly heterogeneous and rarely yielded multiple studies reporting on the same outcome, we did not include a formal certainty of evidence summary.

*5. The manuscript would benefit from a brief limitations section. In line with the PRISMA checklist, it would be helpful to discuss both the limitations of the included evidence and any limitations of the review process, as this would improve transparency and help readers interpret the findings more appropriately.*

**Answer:** Our discussion has been expanded to address the limitations of the included evidence regarding both data availability and methodological quality. We have also elaborated on our screening procedures and other potential limitations of the review process itself. In our previous responses, we highlight the specific excerpts addressing these points which are incorporated across multiple paragraphs in the Discussion section.

*6. Based on PRISMA flow diagram provided in the supplementary material (Supplementary Figure 1), the diagram appears to contain numerical inconsistencies. Specifically, the reported numbers of records screened, records excluded, and reports assessed for eligibility do not reconcile, leaving approximately 300 records unaccounted for. As the PRISMA flow diagram is essential for ensuring transparency and reproducibility of the study selection process, please review and correct the reported figures so that all stages of screening are internally consistent.*

**Answer:** We have updated the PRISMA flow diagram to reflect our August 2026 search update. All figures have been revised and double checked for consistency.

*7. The manuscript would benefit from a clearer presentation of the risk-of-bias judgments and a more precise description of the study designs in Table 4. While the use of Risk of Bias 2 (RoB 2) and Joanna Briggs Institute (JBI) critical appraisal tools is stated (page 7–8), reporting the study-level or domain-level assessments would strengthen transparency and help readers interpret the findings more confidently. In addition, the included evidence appears to comprise cross-sectional and mixed-methods studies alongside controlled and uncontrolled designs. Clearer classification of study design is important because it allows readers to see whether the appropriate critical appraisal tool was applied to each study type, thereby strengthening the transparency, consistency, and credibility of the quality assessment.*

**Answer:** We have detailedly addressed RoB in previous points. We copy our response to Reviewer #2: We have now added an additional column to Table 1 to present the risk of bias (RoB) for prevalence studies reporting mental health estimates. Similarly, Table 4 includes a column reporting the RoB for each intervention study. Unfortunately, due to the highly heterogeneous scope and methodologies of the prevalence reports on other outcomes, we could not provide a uniform brief summary for all of them. Instead, we extensively detail the methodological data supporting such assessments (such as the use of probabilistic versus non-probabilistic sampling and the validity of assessment scores) both in the text and within the tables. For instrument studies, psychometric validation is fully reported utilizing COSMIN grading codes. We have also included the complete risk of bias assessment sheets in the S3 Dataset." Moreover, we have now clearly indicated which RoB tool was used in intervention studies presented in Table 4 (Cochrane RoB 2 for the single RCT; and the Joanna Briggs Institute (JBI) checklist for non-randomized designs for the remaining trials). We have also indicated quality concerns when reporting findings.

[Results] “National data points on mental health disorders were limited to screening cut-off scores reported at the 2022-2023 Mozambique Demographic and Health Survey [(55,61)](https://paperpile.com/c/Z8hP6m/AQfJ+AuWO). This study involved 4548 participants aged 15 - 19 years-old completing the Generalized Anxiety Disorder 7-item (GAD-7) and the Patient Health Questionnaire 9-item (PHQ-9). It was estimated that 6·3% to 18·51% presented significant symptoms of generalized anxiety disorders (GAD-7 ≥ 5 or ≥ 7), whilst 6,6% to 10,25% experienced significant major depressive disorder symptoms (PHQ-9 ≥ 8 or ≥ 10). Although the Disrupting Harm project utilized a nationally representative survey of Mozambican adolescents incorporating structured scales, prevalence estimates relied on single-item responses rather than validated cut-off scores [(66)](https://paperpile.com/c/Z8hP6m/x0pA).”

[Results] “The RCT assessed a World Bank program among 1,907 preschool children in high-poverty areas [(30)](https://paperpile.com/c/Z8hP6m/qCW5). It demonstrated significant developmental gains with an implementation cost of approximately $3 per child per month. However, it was judged to have a high risk of bias across all assessed domains, including selection bias, blinding of participants and outcome assessors, outcome measurement, and selective outcome reporting.”

*8. The discussion would benefit from a more critical and balanced interpretation of the findings. At present, it largely repeats the results and focuses on implications and future research, but it does not sufficiently engage with previous studies, including those with conflicting or contrasting results. In addition, it does not address risk of bias, heterogeneity, differences in study design, or the strength of the evidence. Strengthening the discussion with more direct support from the literature and aligning the conclusions more closely with the scope and quality of the included evidence would improve the credibility and balance of the manuscript.*

**Answer:** We have fully addressed this issue in our response to point 5. We also clarify the lack of conflict results, as the available literature was scattered and seldom yielded multiple studies on the exact same subject.

*Minor comments:
1. Overall, the manuscript is well organized. However, I believe it would benefit from a careful revision of the writing to improve the flow and readability, particularly in the Results and Discussion sections. In several places, the presentation is quite concise, making it difficult to fully appreciate the progression of the findings and their interpretation. Expanding key explanations where appropriate and improving the transitions between ideas would strengthen the overall presentation.*

**Answer:** We have carefully revised both the Results and Discussion sections, with all improvements highlighted throughout the text.

*2. The readability of the manuscript could also be improved by organizing the presentation of the included studies more systematically. For example, Table 1 might be easier to follow if the studies were grouped by sample recruitment setting (e.g., nationwide, regional, then school-based) and, where appropriate, by diagnostic criteria. Similarly, Table 2 could be organized by recruitment setting and type of adversity exposure, while Table 4 might benefit from grouping studies according to intervention target and setting. A more consistent organization across the tables may help readers identify patterns and compare studies more easily.*

**Answer:** We have now added a section in the methods outlining the rationale and structure for data synthesis and presentation. Data is presented per area (prevalence, instrument, intervention) and, within each area, grouped per diagnostic domain and sorted according to methodological characteristics. While other possible grouping strategies would be certainly possible, we retained the current approach for aligning with the framework reported in previous national compilation reviews. We claim this format suited the evidence base identified in Mozambican literature, supporting an informative, comprehensive catalogue of findings, while also accounting for the heterogeneity across populations, study methodologies, and outcomes.

*3. The study selection process is described inconsistently between the main text and Supplementary Table 2. Page 6 states that two independent authors conducted title/abstract screening and a single reviewer performed full-text screening, whereas Supplementary Table 2 indicates that two independent reviewers conducted both primary and secondary screening (title/abstract and full-text screening). Please reconcile these descriptions and clarify the actual screening procedure used.*

**Answer: W**e have double-checked the sections and corrected the inconsistency in the Supplementary Material. To clarify, for studies retrieved from international databases (PubMed, PsycInfo, CINAHL, and Web of Science), two independent reviewers assessed eligibility during the primary screening (title/abstract), and a single reviewer conducted the full-text screening.

*4. According to PRISMA 2020 item #8, the review should report how automation tools were integrated into the study selection process. If records were removed by automation before screening, this should be shown in the PRISMA flow diagram as “Records marked as ineligible by automation tools,” similar to how deduplication is reported.*

**Answer:** We reproduce our response to Reviewer #2: We thank the reviewer for raising this important methodological point. Our initial description lacked clarity: we did not use a standard "similarity index", but the “Overall Similarity Percentage”, which is a specific metric provided by Rayyan platform. It calculates similarity considering all metadata fields (including titles, authors, dates, database, and DOIs). As references exported from different databases inherently contain formatting variations, a 100% similarity score is exceedingly rare, and even highly similar titles would not result in exact matches.

We make reference to a review assessing the performance of this metric (doi:[10.2196/94317](https://doi.org/10.2196/94317)): *“Higher thresholds of ≥95% approximate exact matching with tolerance for minor variation and carry a lower risk of false positives. Lower thresholds increase the number of duplicates detected but require more careful manual review of flagged records. We recommend beginning at ≥95% and reviewing the results before considering a lower threshold.”.*

The manuscript has been revised to more clearly detail deduplication procedures:

[Methods] “The review was managed using Rayyan software. Potential duplicates were detected, automatically removing pairs with a 100% match in title/DOI or Overall Similarity Percentage above 95% (a metric calculated based on all characters and metadata associated with the reference); remaining candidates were manually verified. This was informed by an assessment of the deduplication tool, which suggested that an initial 95% cut-off carries a negligible risk of false exclusions **(17).”**

*5. The eligibility criteria state that studies were included if they reported specific data for samples in Mozambique with an average age up to 19 years old, regardless of outliers. However, on Table 1, 2, and 4, several included studies appear to involve age ranges extending beyond 19 years. To avoid potential confusion, it would be helpful to report the mean age for each study, if available, along with the standard deviation, rather than only the age range.*

**Answer:** Unfortunately, mean age and standard deviation data were often missing, especially in broader population studies. To align with the comprehensive scope of our national mapping, we opted to include studies with incomplete information (e.g., when the exact average age was not available) if they presented subgroup data for age ranges of interest.

*6. In the page 7–8 “Methodological quality was assessed with the revised Cochrane tool for randomized trials (RoB 2) and the Joanna Briggs Institute (JBI) checklist for non-randomized designs.” Please provide additional details on who performed the assessments, whether reviewers worked independently, how disagreements were resolved, and how the tool outputs were incorporated into the review process, as recommended by PRISMA.*

**Answer:** As addressed in previous points, we have detailed how the risk of bias assessments were presented and incorporated into the discussion of our findings. We now clarify the data extraction and quality appraisal procedures, which were conducted by a single reviewer and verified by a second researcher:

[Methods] “A single reviewer performed the initial extraction and quality appraisal, and all datasheets were subsequently verified by LEM.”
 *7. In Table 4, the “effects” column is unclear. Please specify what effect was measured for each intervention. If it is regarding the effect of the interventions, please report the corresponding estimate and precision measures, such as the point estimate, 95% confidence interval, and p value where available, rather than only stating “statistically significant improvement...”. Additionally, Table 4 should include a note explaining the meaning of the symbols “+” and “?” used in the effect column. At present, the table is not fully interpretable without a legend defining these symbols.*

**Answer:** In Table 4, we have now excluded the column synthesizing efficacy as + or - as it was imprecise. Instead, we present key findings in a dedicated text column. Additionally, S2 Dataset now provides full data sheets with comprehensive metrics.

*8. In Page 28, second paragraph, the study being discussed should be cited consistently every time it is mentioned. Please add the relevant reference after each statement derived from that study to improve clarity and ensure proper attribution. The same issue also appears in Page 30, second paragraph, where the study should likewise be cited consistently throughout the discussion.*

**Answer:** We have corrected it.

*9. In page 29, first paragraph, the statement “Similarly, other register-based studies in Mozambique are scarce and rely primarily on service-level data from isolated facilities, limiting their generalizability” should be cited. Please add the relevant reference to support this claim.*

**Answer:** We have corrected it.

*10. In the page 31, the conclusion appears stronger than the evidence presented. While the review suggests an emerging field and important gaps in screening tools and culturally adapted instruments, the certainty of evidence is not clearly summarized, and the strength of these claims should be tempered accordingly. Please align the conclusion more closely with the quality and certainty of the underlying evidence and explicitly acknowledge the limitations of the included studies.*

**Answer:** We have addressed these concerns which are detailed in previous responses. We highlight the following alteration in conclusion:

[Discussion] “The field of child and adolescent mental health is still emerging in Mozambique. While available data is scarce, it suggests a high burden of mental disorders, with public health priorities shaped by widespread socioeconomic adversity and alarming rates of gender-based violence. There are few available resources for screening mental health symptoms, and reliable assessment warrants cultural adaptation of instruments. Future research should target data gaps on prevalence rates of mental disorders across regional and national samples. There is an urgent need to strengthen local research capacity through investment in human resource development and the expansion of academic institutions.”
