# Supplementary material for: The science of child and adolescent mental health in Mozambique: A systematic review of prevalence estimates, measurement tools, and interventions
Source: PLOS Glob Public Health. 2026 Oct 23;6(10):e0007513. doi: 10.1371/journal.pgph.0007513 (PMC13693922; doi:10.1371/journal.pgph.0007513)
Supplement: Supplementary file 3 [file pgph.0007513.s006.docx]

Dear Editorial Office,

We sincerely thank the Editor and the Reviewers for their thorough evaluation of our manuscript and for their positive assessment of our work. We have addressed all minor revision recommendations raised by Reviewers #1 and #4 (as detailed in the point-by-point response below). These revisions have enhanced the clarity and transparency of reporting, whilst the core manuscript components remained unchanged.

Please refer to the following hyperlinks for direct access:

- **Journal requirements**
- **Reviewer #1**
- **Revirewer #3**
- **Reviewer #4**

## JOURNAL REQUIREMENTS

*1. Figure Resubmissions - While revising your submission, we strongly recommend that you use PLOS’s NAAS tool (https://ngplosjournals.pagemajik.ai/artanalysis) to test your figure files.* **Answer:** We have submitted revised figure files that meet all format specifications following the adjustments recommended by the tool.

*2. If the reviewer comments include a recommendation to cite specific previously published works, please review and evaluate these publications to determine whether they are relevant and should be cited. There is no requirement to cite these works unless the editor has indicated otherwise.* **Answer:** No recommendation to cite specific publications was made by the reviewers.

*3. Please review your reference list to ensure that it is complete and correct. If you have cited papers that have been retracted, please include the rationale for doing so in the manuscript text, or remove these references and replace them with relevant current references. Any changes to the reference list should be mentioned in the rebuttal letter that accompanies your revised manuscript. If you need to cite a retracted article, indicate the article’s retracted status in the References list and also include a citation and full reference for the retraction notice.* **Answer:** We reviewed the reference list to ensure its completeness and accuracy and confirmed that no cited studies have been retracted. One additional citation was included in the Introduction and Methods sections to provide context regarding previous national systematic reviews (Brooke-Summer et al. 2026; listed as reference 12). No other changes were made to the reference list.

## REVIEWER #1

*I have made substantial revisions to the manuscript and have adequately addressed the major concerns raised in the previous round of review. The revised manuscript is clearer and more transparent, particularly regarding the search strategy, study selection, risk of bias assessment, reporting of the findings, and discussion of limitations. I have only a few minor points that I recommend addressing before publication. These points do not substantially affect the overall value or conclusions of the review and should be relatively straightforward to address. Overall, the manuscript has been considerably improved and provides a valuable synthesis of the available evidence on child and adolescent mental health in Mozambique. I believe it is suitable for publication following these minor revisions.*

**Answer:** We thank the reviewer for this positive comment. All minor revision recommendations have been incorporated in the manuscript. We outline changes below.

*1. The authors should provide a little more clarity regarding the single reviewer screening process and the extent to which the 20% independent verification identified any disagreements or additional eligible studies.*

**Answer:** We have expanded the Methods section to provide additional detail regarding the single-reviewer screening approach and the independent verification process. Although corrections made during the 20% independent verification were not systematically recorded, no major discrepancies nor missed eligible studies were identified during this process. As I myself conducted this verification, I can only recall excluding a small number of reports that, upon closer inspection, revealed to be duplicates (e.g., a thesis reporting the findings that were already included from a peer-reviewed article). This consistency likely reflects the fact that the very same research team conducted the screening/extraction procedures of the Brazilian review, engaged in pilot calibration exercise for the present work, and adopted an approach prioritizing sensitivity over specify (in doubt, reports were included).

The revised manuscript now clarifies both the rationale for the single-reviewer approach and the procedures used to maintain consistency and reliability throughout the review process.

**[METHODS]** “The research team responsible for screening and data extraction (LEM, ACC, and JAV) was the same that had conducted these procedures in the national review in Brazil [(10)](https://paperpile.com/c/5tLXZo/kw3b). Prior to primary and secondary screening, a pilot calibration exercise was performed on 10% of the dataset to align inclusion decisions across reviewers. Regular meetings were held throughout screening and extraction to maintain consistency and shared understanding of criteria. When adopting single-reviewer screening procedures, 20% of the records were randomly verified by a second reviewer (LEM); this author also resolved discrepancies arising from two author independent screening. This strategy draws on prior national reviews which utilized a single-reviewer approach emphasizing sensitivity over specificity to maximize article inclusion (i.e., when in doubt, studies were included to make the review as comprehensive as possible) [(9–12)](https://paperpile.com/c/5tLXZo/g8kG+kw3b+QHMg+5BWJ). The rationale is fully detailed in the Brazilian systematic review [(10)](https://paperpile.com/c/5tLXZo/kw3b). In brief, this approach is particularly well-suited to compilation reviews and has demonstrated reliable performance in empirical evaluations informing Cochrane guidance [(15–17)](https://paperpile.com/c/5tLXZo/VjQdr+XD333+gVS5O).”

**[DISCUSSION]** "Although screening was often conducted by a single reviewer, the emphasis on maximizing inclusion and identifying as many relevant studies as possible helped reduce the risk of missing important evidence, and no major discrepancies were identified during the independent verification of screening and data extraction (this likely reflects the reviewers' prior experience with a similar review conducted in Brazil, as well as pilot calibration exercise and ongoing discussions throughout the review process).”

*2. Given the limited number and geographical concentration of studies providing clinical prevalence estimates, the authors should continue to be cautious when discussing the overall burden of mental disorders in Mozambique and clearly distinguish between lack of evidence and evidence of absence.*

**Answer:** We have thoroughly checked and refined the manuscript to ensure these limitations and data gaps are clearly reported when presenting results and interpreting findings. In particular, we reinforced major points across the revised Abstract and Discussion:

**[ABSTRACT] “**Children and adolescents in Mozambique face significant psychosocial adversity. While there is very limited clinical data, available evidence suggests a substantial burden of mental disorders. There are few available instruments assessing mental conditions, and culturally-sensitive approaches are warranted for strengthening local research.”

**[DISCUSSION]** “With significant data gaps, the current evidence in Mozambique is far from conclusive, warranting caution when drawing comparisons with international indicators. Clinical data is limited to two urban samples comprising a total of 1,234 participants, yet point to a higher prevalence of mental health disorders compared to global estimates assessed with similar manualized criteria [(10,93)](https://paperpile.com/c/5tLXZo/2utGI+kw3b). Although suicide rates in Mozambique appear lower than then global averages (ages 15–19: 1.53; ages 10–14: 0.59 per 100,000), they are likely underestimated [(94)](https://paperpile.com/c/5tLXZo/ZBqQl). The single national study was based on three referral services responsible for all violent death autopsies in the country [(28)](https://paperpile.com/c/5tLXZo/OV9Kt). Moreover, the reliability of epidemiological and civil registration data in Mozambique is limited, with reports that nearly half of children under the age of five are not registered in official records [(3)](https://paperpile.com/c/5tLXZo/6on5d). Similarly, other register-based studies in Mozambique are scarce and rely primarily on service-level data from isolated facilities, limiting their generalizability. An integrated national health information system that includes mental health outcomes is needed to strengthen epidemiological data and monitoring across the country [(95)](https://paperpile.com/c/5tLXZo/kvfgn).”

*3. The limitations associated with heterogeneity of study designs, assessment instruments, and populations should remain clearly reflected when interpreting and comparing prevalence estimates.*

**Answer:** Given the manageable volume of available literature, we were able to report detailed characteristics for most included studies, providing granular information on scope, methods, populations, recruitment procedures, and assessment criteria. We have revised the Discussion to more clearly synthesize the sources of heterogeneity and their implications for interpreting the results.

**[DISCUSSION]** “With significant data gaps, the current evidence in Mozambique is far from conclusive, warranting caution when drawing comparisons with international indicators. Clinical data is limited to two urban samples comprising a total of 1,234 participants, yet point to a higher prevalence of mental health disorders compared to global estimates assessed with similar manualized criteria (10,93).”

**[DISCUSSION]** “Given the limited scope of local research, these sources could represent a substantial body of evidence. Meta-analysis was not feasible due to the limited number and substantial heterogeneity of the included studies, which yielded few comparable data points. For instance, no single prevalence estimate or measurement property was evaluated across two or more studies with comparable samples (notably the two community-based studies using clinical interviews did not report on the same mental disorders, further relying on distinct diagnostic manuals and age ranges). This heterogeneity also precluded a formal certainty-of-evidence assessment (e.g., GRADE) given the lack of overlap across reports.”

## REVIEWER #3

*This research report is exceptionally well-structured, thorough, and insightful. The author demonstrates a clear understanding of, with strong methodology, relevant data, and logical analysis. What I found most impressive was the clarity of presentation - complex findings are explained in an accessible way, supported by credible sources and well-organized evidence. The conclusions are well-grounded and offer practical implications. It is a valuable contribution to the field and I would highly recommend it to anyone interested in child and adolescent psychiatry. It's an excellent work.*

*Strengths:*

*1. Relevance & Originality:* The report addresses a timely and important problem in.. 2. Methodology:* The research design is sound, rigorous, and appropriate for the research questions.*

*3. Analysis & Clarity:Data is analyzed comprehensively and presented with great clarity. Figures/Tables are effective.*

*4. Conclusion: Findings are well-supported and the recommendations are actionable.*

*Overall, this is a high-quality report that reflects deep research and critical thinking. It meets academic and professional standards with distinction.*

*An excellent and well-researched report! Very informative, well-organized, and data-driven. The author did a fantastic job breaking down into clear, actionable insights. Highly recommended..*

**Answer:** We thank the reviewer for this careful appraisal and positive comments on your work.

## REVIEWER #4

*The revised manuscript has been considerably improved, and the authors are to be commended for their careful attention to the comments raised in the previous round. This review addresses an important and underexplored area of child and adolescent mental health in Mozambique, and its nationwide scope, comprehensive search strategy, inclusion of grey literature, and synthesis of prevalence estimates, assessment instruments, and interventions remain important strengths of the study. The revisions have also improved the clarity and overall presentation of the manuscript. Most of the concerns raised previously have been adequately addressed, with only a few minor points remain for consideration. The following minor points are suggested for further consideration:*

**Answer:** We thank the reviewer for such encouraging assessment of our work. All suggested minor points have been incorporated and are detailed below.

*1. The authors explain in their response that a formal certainty-of-evidence assessment was not conducted given the substantial heterogeneity across the included studies. However, the implications of this heterogeneity for the interpretation of the findings should be more clearly reflected in the manuscript. I suggest that the authors explicitly describe the main sources of heterogeneity across the studies (e.g., differences in outcome definitions or assessment instruments). This would help readers understand that the reported findings should be interpreted with caution and may not be directly comparable or conclusive.*

**Answer:** This concern was also raised by Reviewer #1. We reproduce our response to that comment and the corresponding modifications made to the text: Given the manageable volume of available literature, we were able to report detailed characteristics for most included studies, providing granular information on scope, methods, populations, recruitment procedures, and assessment criteria. We have revised the Discussion to more clearly synthesize the sources of heterogeneity and their implications for interpreting the results.

**[DISCUSSION]** “With significant data gaps, the current evidence in Mozambique is far from conclusive, warranting caution when drawing comparisons with international indicators. Clinical data is limited to two urban samples comprising a total of 1,234 participants, yet point to a higher prevalence of mental health disorders compared to global estimates assessed with similar manualized criteria (10,93).”

**[DISCUSSION]** “Given the limited scope of local research, these sources could represent a substantial body of evidence. Meta-analysis was not feasible due to the limited number and substantial heterogeneity of the included studies, which yielded few comparable data points. For instance, no single prevalence estimate or measurement property was evaluated across two or more studies with comparable samples (notably the two community-based studies using clinical interviews did not report on the same mental disorders, further relying on distinct diagnostic manuals and age ranges). This heterogeneity also precluded a formal certainty-of-evidence assessment (e.g., GRADE) given the lack of overlap across reports.”

*2. There appears to be lack of clarity in the reporting of the number of studies, reports, and publications. For example, in the page 9, “…61 prevalence reports are described as comprising 15 studies on mental health disorders, 9 on substance use, 15 on psychosocial adversities, and 9 on other psychosocial symptoms”, which sum to 48. In addition, the manuscript reports 29 reports on assessment instruments and 9 reports describing intervention trials, while the total number of unique publications is stated as 81. Please clarify how these numbers relate to one another, including the distinction between a study, report, and publication, and ensure that the numbers are consistent throughout the manuscript. It may also be helpful to add the corresponding number of studies for each domain or theme in the PRISMA flow diagram, so that readers can more easily follow how the reports relate to the total number of unique studies.*

**Answer:** We thank the reviewer for raising this point. We revised the manuscript to clarify use of terms and ensure consistent reporting. As per PRISMA definitions, we consider a report to be any specific document or publication included in the review, whilst a study refers to the actual underlying research project (such as a survey, cohort, or clinical trial). This way, several reports may refer to a single study; this is the case of references 56 and 62, which are two reports from the same parent study (2022-2023 Mozambique Demographic and Health Survey). In turn, this single survey could count across multiple domains (a study on mental health conditions, a study on substance use, and a study on childhood adversities); we have therefore removed this secondary classification count to avoid confusion.

As our umbrella review has three independent components, some amendments were necessary to the PRISMA diagram. While the full review included 81 unique reports, each of them could count as an inclusion in one or more areas: there were 61 prevalence reports, 29 assessment instrument reports, and 9 intervention reports. Due to overlaps, the sum of inclusions per area surpasses the number of unique inclusions in the full review.

The operational definition of a study also varied by area. A prevalence study represents the underlying dataset supporting one or more publications (we included 45 prevalence studies described across 61 prevalence reports). An intervention study represents an individual trial that may be described across multiple publications (we included 8 trials described across 9 intervention reports). In turn, no equivalent definition of a parent study was feasible for assessment instruments: data on measurement tools were typically scattered across publications that evaluated multiple distinct instruments and reported varied psychometric properties for each. We therefore decided not to group them under parent studies, reporting instead the total number of relevant publications (29) alongside the count of unique assessment instruments (42) described across them.

In accordance, we made the following revisions to the manuscript:

**[FIGURES]**

- **Figure 1 - Flowchart of inclusions**: corrected inconsistencies for reflecting standardized terminology differentiating studies from reports.
- **Figure 2 - Availability of evidence**: revised labels for consistency.
- **S1 Appendix. Figure A - PRISMA Flowchart**: provided the definition of “report” and “study” and added specific numbers for each area (prevalence, instrument, intervention).

**[ABSTRACT]** “The search yielded 81 unique publications, including 61 reports presenting prevalence estimates from 25 studies, 29 reports providing data on 42 assessment instruments, and 9 intervention reports describing 8 intervention trials.”

**[RESULTS]** “[Figure 1](https://docs.google.com/document/d/1xR3dUIrxSA1N48RzkPZbYuThVX1vx9dDJHdr7msy_48/edit?tab=t.0#heading=h.p5mv5gty5h00) presents a flowchart with search outcomes (see Figure A in S1 Appendix for PRISMA flowchart; see Table H in S1 Appendix for exclusion reasons; S2 Dataset - Screening provides full access to original search files and documents all screening decisions). Following the screening of 5,760 records, 81 unique publications were eligible for inclusion in one or more areas: 61 reports on prevalence estimates from 25 datasets, 29 reports containing data on 42 assessment instruments, and 9 reports detailing 8 intervention trials. S3 Dataset - Extraction contains the full data sheets, which detail all included reports, extracted data, and risk of bias assessments.”

**[RESULTS] “**[Table 1](https://docs.google.com/document/d/1xR3dUIrxSA1N48RzkPZbYuThVX1vx9dDJHdr7msy_48/edit?tab=t.0#heading=h.sa4ucxhsm3pa) presents estimates on the prevalence of mental health disorders. There were only 3 studies including clinical data according to manualized criteria. A study in Maputo recruited 486 adolescents, leading to three reports estimating the prevalence of any mental diagnosis (23%), any anxiety disorders (17·5%), major depression episode (8·5%), disruptive behavior disorder (6·6%), substance use disorders (4·5%), ADHD (3·3%), and PTSD (1·2%) [(8,57,58)](https://paperpile.com/c/5tLXZo/J6hwl+yQGN9+3DHxh). Another survey in Nampula included 748 children and adolescents, with two reports on the prevalence of ADHD at 13·4% [(59,60)](https://paperpile.com/c/5tLXZo/pReM+vOea). Additionally, a non-probabilistic study included 110 individuals aged 1-20, using clinical batteries to estimate the prevalence of neurodevelopmental impairment (mild: 18·2%; moderate-to-severe: 0·9%) [(61)](https://paperpile.com/c/5tLXZo/fieW4).”

**[DISCUSSION]** “This systematic review synthesizes evidence on child and adolescent mental health in Mozambique, encompassing 61 reports from 25 prevalence studies, 29 reports describing 42 assessment instruments, and 8 intervention trials. “

*3. There are some discrepancies that would benefit from clarification in the reporting of the numbers throughout the manuscript and PRISMA flow diagram. In the PRISMA diagram, 451 reports are indicated as excluded, whereas the numbers provided by the different reasons for exclusion appear to sum to 440, leaving 11 reports unaccounted for. Please clarify this discrepancy.*

**Answer:** The original PRISMA flow diagram did not sufficiently account for our three-arm review design. We have amended the diagram to clarify the total number of included publications as well as the specific study and report counts within each domain. In addition, because full-text eligibility was appraised separately for each area (prevalence, assessment instruments, and interventions), exclusion reasons cannot be cleanly aggregated without overlap. We have clarified this distinction, providing the overall sum of exclusion reasons in the flow diagram and referring to S1 Table H for a detailed breakdown across each review area.

**[S1 APPENDIX]**

**Figure A - PRISMA Flowchart.**

“Reports excluded: (n = 451)

*Overall reasons**

*Wrong sample (n = 361)*

*Wrong study design (n = 189)*

*Background article (n = 28)*

*Wrong publication type (n = 22*

*Wrong outcome (n = 11)*”

“Notes: *This is a raw sum of reasons for exclusion across prevalence, instrument, and intervention reports screenings; see S1 Appendix Table H for detailed reasons according to each specific area”

*4. In addition, the PRISMA diagram (supplementary material) reports 81 reports included in the review, while only 45 studies are reported as being included. While multiple reports may originate from the same study, the relationship between reports and studies should be made clearer. For example, where multiple reports relate to the same study or represent follow-up or subsequent publications from the same study, it would be helpful to clarify how these were identified. This would help readers understand how the 81 reports were consolidated into 45 studies and ensure that the included evidence is not double counted.*

**Answer:** As noted above, we have updated the PRISMA flow diagram to accurately reflect the structure of our three-arm review and detail findings across domains:

**[S1 APPENDIX]**

**Figure A - PRISMA Flowchart.**

“**Unique reports included (n = 81)**.

- Prevalence reports (n = 61)
  - *Prevalence studies (n = 25)*
- Instrument reports (n = 29)
  - *Instruments reported (n = 42)*
- Intervention reports (n = 9)
  - *Intervention studies (n = 8)*”

“Notes: **Records**: any entry/result registered during screening. **Report**: any specific document or publication. **Study**: underlying research project (database survey or clinical trial) that may ground multiple reports.

*5. Figure 2 (page 10) presents the availability of evidence-based resources across the different domains of inquiry. However, the figure is not sufficiently described in the main text. It would be helpful to briefly summarize the key findings presented in the figure, rather than leaving the interpretation entirely to the reader.*

**Answer:** In accordance with this recommendation, we have incorporated an in-text summary highlighting the primary findings presented in Figure 2:

**[RESULTS]** “[Figure 2](https://docs.google.com/document/d/1xR3dUIrxSA1N48RzkPZbYuThVX1vx9dDJHdr7msy_48/edit?tab=t.0#heading=h.g88zwb60u9qj) presents the extent of evidence-based resources, while [Figure 3](https://docs.google.com/document/d/1xR3dUIrxSA1N48RzkPZbYuThVX1vx9dDJHdr7msy_48/edit?tab=t.0#heading=h.q6l1d87xekjv) shows temporal trends of publications. There were 75 peer-reviewed articles, 4 theses/dissertations, and 2 institutional research reports. Research output in Mozambique is relatively recent, with the vast majority of reports published in the past decade. The evidence base is skewed toward prevalence estimates, featuring very few intervention trials. Childhood adversity and trauma constitute a prominent line of inquiry, accounting for 28 of the included reports. Prevalence reports are also centered on substance use, and assessment instruments have a substantial concentration on neurodevelopment tests..”

*6. For the non-randomized studies, the supplementary file provides the Yes/No/Unclear/Not Applicable responses to the JBI checklist. Given that the critical appraisal was conducted by a single reviewer (page 7), it would be helpful if the authors could consider adding a “Justification” column, as provided in the JBI checklist, to briefly explain the basis for each judgement of each question. This would further enhance the transparency of the appraisal process and allow readers to better understand how the judgements were made..*

**Answer:** To maximize accessibility and transparency, we incorporated an explicit summary of bias appraisal directly into the main intervention table (Table 3). This allows readers to immediately contextualize each study’s overall risk-of-bias judgement for different study designs without navigating loads of supplementary datasheets. We reproduce below the summary provided for each study:

**[RESULTS - Table 3]**

**“Study 1**:. Cluster allocation; post-randomization reassignment of groups; differences in control group; voluntary participant; only 41% of eligible children enrolled; unblinded.

**Study 2:** Control group was not comparable; lack of valid measurement; unblinded assessment

**Study 3**: No baseline assessment; lack of valid instrument; families participating in the intervention might be more prone to benefits; no distal outcome assessing direct effects

**Study 4:** No baseline assessment; lack of valid instrument; indirect intervention delivered with teachers to benefit girls; no distal outcome assessing direct effects

**Study 5:** No control group; lack of valid tool; no distal outcome assessing direct effects; convenience sample of girls participating in the project

**Study 6:** No baseline assessment; differences in control and intervention group; unblinded assessment; lack direct distal outcomes

**Study 7:** No control group; researchers not blinded; lack of valid instrument; direct distal outcomes were not reported

**Study 8:** No control group; limited sample size; unblinded; proxy-reported measures; multiple statistical tests without prior hypothesis or adjustments.”
