# Supplementary material for: The science of child and adolescent mental health in Mozambique: A systematic review of prevalence estimates, measurement tools, and interventions
Source: PLOS Glob Public Health. 2026 Oct 23;6(10):e0007513. doi: 10.1371/journal.pgph.0007513 (PMC13693922; doi:10.1371/journal.pgph.0007513)
Supplement: Peer review history

# Peer review: Decision Letter 0

**DOI:** 10.1371/journal.pgph.0007513.r001

**Authors:** Nawi Ng

19 Jul 2026

PGPH-D-26-01930

The science of child and adolescent mental health in Mozambique: a nationwide systematic review of prevalence rates, assessment instruments, and interventions

PLOS Global Public Health

Dear Dr. Marchinatti,

Thank you for submitting your manuscript to PLOS Global Public Health. After careful consideration, we feel that it has merit but does not fully meet PLOS Global Public Health’s publication criteria as it currently stands. Therefore, we invite you to submit a revised version of the manuscript that addresses the points raised during the review process.

Please submit your revised manuscript by Sep 17 2026 11:59PM. If you will need more time than this to complete your revisions, please reply to this message or contact the journal office at globalpubhealth@plos.org. When you're ready to submit your revision, log on to https://www.editorialmanager.com/pgph/ and select the 'Submissions Needing Revision' folder to locate your manuscript file.

Please include the following items when submitting your revised manuscript:

- A letter that responds to each point raised by the editor and reviewer(s). You should upload this letter as a separate file labeled 'Response to Reviewers'.
- A marked-up copy of your manuscript that highlights changes made to the original version. You should upload this as a separate file labeled 'Revised Manuscript with Track Changes'.
- An unmarked version of your revised paper without tracked changes. You should upload this as a separate file labeled 'Manuscript'.

Guidelines for resubmitting your figure files are available below the reviewer comments at the end of this letter.

As the corresponding author, your ORCID iD is verified in the submission system and will appear in the published article. PLOS supports the use of ORCID, and we encourage all coauthors to register for an ORCID iD and use it as well. Please encourage your coauthors to verify their ORCID iD within the submission system before final acceptance, as unverified ORCID iDs will not appear in the published article. *Only*  the individual author can complete the verification step; PLOS staff *cannot*  verify ORCID iDs on behalf of authors.

We look forward to receiving your revised manuscript.

Kind regards,

Nawi Ng

Academic Editor

PLOS Global Public Health

Journal Requirements:

1. We note that the most recent literature search was completed over a year before the manuscript was submitted to the journal. Systematic and scoping reviews should provide an up-to-date overview of the research topic covered. Therefore, we request that you please update your searches as part of your revision to ensure that the most up-to-date literature is included in your review, or provide scientific justification for not doing so. Please ensure you mention this in your response to reviewers, and discuss whether this has any impact on the Results and Conclusions of the study. If you are submitting a protocol, it is only necessary to state whether the searches will be updated prior to the publication of the results.

2. Please amend your detailed Financial Disclosure statement. This is published with the article. It must therefore be completed in full sentences and contain the exact wording you wish to be published.

a. Please clarify all sources of funding (financial or material support) for your study. List the grants (with grant number) or organizations (with url) that supported your study, including funding received from your institution.

b. State the initials, alongside each funding source, of each author to receive each grant.

c. If any authors received a salary from any of your funders, please state which authors and which funders.

3.Please provide separate figure files in .tif or .eps format.

For more information about figure files please see our guidelines:  LINK

https://journals.plos.org/globalpublichealth/s/figures

https://journals.plos.org/globalpublichealth/s/figures#loc-file-requirements

4. Your manuscript is missing the following sections: Introduction. Please ensure these are present, and in the correct order, and that any references to subheadings in your main text are correct. An outline of the required sections can be consulted in our submission guidelines here: [LINK]

https://journals.plos.org/globalpublichealth/s/submission-guidelines#loc-parts-of-a-submission

5. We have noticed that you have uploaded Supporting Information files, but you have not included a list of legends. Please add a full list of legends for your Supporting Information files after the references list.

6. As required by our policy on Data Availability, please ensure your manuscript or supplementary information includes the following:

A numbered table of all studies identified in the literature search, including those that were excluded from the analyses.

For every excluded study, the table should list the reason(s) for exclusion.

If any of the included studies are unpublished, include a link (URL) to the primary source or detailed information about how the content can be accessed.

A table of all data extracted from the primary research sources for the systematic review and/or meta-analysis. The table must include the following information for each study:

Name of data extractors and date of data extraction

Confirmation that the study was eligible to be included in the review.

All data extracted from each study for the reported systematic review and/or meta-analysis that would be needed to replicate your analyses.

If data or supporting information were obtained from another source (e.g. correspondence with the author of the original research article), please provide the source of data and dates on which the data/information were obtained by your research group.

If applicable for your analysis, a table showing the completed risk of bias and quality/certainty assessments for each study or outcome.  Please ensure this is provided for each domain or parameter assessed. For example, if you used the Cochrane risk-of-bias tool for randomized trials, provide answers to each of the signalling questions for each study. If you used GRADE to assess certainty of evidence, provide judgements about each of the quality of evidence factor. This should be provided for each outcome.

An explanation of how missing data were handled.

This information can be included in the main text, supplementary information, or relevant data repository. Please note that providing these underlying data is a requirement for publication in this journal, and if these data are not provided your manuscript might be rejected.

7. We note that your Data Availability Statement is currently as follows: "All data relevant to the study are included in the article or uploaded as online supplementary information. No additional data are available."

Please confirm at this time whether or not your submission contains all raw data required to replicate the results of your study. Authors must share the “minimal data set” for their submission. PLOS defines the minimal data set to consist of the data required to replicate all study findings reported in the article, as well as related metadata and methods (https://journals.plos.org/plosone/s/data-availability#loc-minimal-data-set-definition).

For example, authors should submit the following data:

- The values behind the means, standard deviations and other measures reported;

- The values used to build graphs;

- The points extracted from images for analysis.

Authors do not need to submit their entire data set if only a portion of the data was used in the reported study.

If your submission does not contain these data, please either upload them as Supporting Information files or deposit them to a stable, public repository and provide us with the relevant URLs, DOIs, or accession numbers. For a list of recommended repositories, please see https://journals.plos.org/plosone/s/recommended-repositories.

If there are ethical or legal restrictions on sharing a de-identified data set, please explain them in detail (e.g., data contain potentially sensitive information, data are owned by a third-party organization, etc.) and who has imposed them (e.g., an ethics committee). Please also provide contact information for a data access committee, ethics committee, or other institutional body to which data requests may be sent. If data are owned by a third party, please indicate how others may request data access.

**Reviewers' comments to the Authors**

publication criteria?

Reviewer #1: Yes

Reviewer #2: Yes

Reviewer #3: Yes

Reviewer #4: Partly

**********

2. Has the statistical analysis been performed appropriately and rigorously?

Reviewer #1: Yes

Reviewer #2: N/A

Reviewer #3: No

Reviewer #4: N/A

**********

3. Have the authors made all data underlying the findings in their manuscript fully available (please refer to the Data Availability Statement at the start of the manuscript PDF file)?

The PLOS Data policy

Reviewer #1: Yes

Reviewer #2: Yes

Reviewer #3: Yes

Reviewer #4: Yes

**********

4. Is the manuscript presented in an intelligible fashion and written in standard English?

Reviewer #1: Yes

Reviewer #2: Yes

Reviewer #3: Yes

Reviewer #4: Yes

**********

**Reviewer #1:**

Thank you for the opportunity to review this manuscript. This is a highly timely and valuable piece of work for global public health, specifically within child and adolescent mental health.

The authors have done a great job pulling together and synthesizing the scattered evidence on youth mental health in Mozambique. By mapping out prevalence data, existing assessment tools, and current interventions, this paper fills a major knowledge gap. It has real potential to serve as a practical guide for policymakers, researchers, and development partners looking to strengthen mental health infrastructure in the region.

Key Strengths

1.Comprehensive Scope: The decision to track prevalence, tools, and interventions all in one place provides a remarkably thorough overview.

2.Rigorous Approach: The systematic review methodology is solid and aligns well with standard reporting guidelines.

3.Critical Lens: The discussion doesn't just list data; it does a great job highlighting where the current research and service delivery fall short.

4.Cultural Context: The focus on culturally adapted assessment tools is excellent and highly relevant for low-resource settings where Western tools often miss the mark.

5.Actionable Next Steps: The recommendations for future research and capacity-building are practical and forward-looking.

Suggestions for Improvement

1.Policy Integration: It would be great to see a brief discussion on exactly how these findings can plug into Mozambique’s current national mental health policy and service planning. Connecting the data directly to local policy frameworks would make the paper even more impactful.

2.Nuance on Prevalence Data: I suggest pushing the limitations of the current prevalence studies a bit further. It’s worth explicitly reminding the reader how few of these studies are actually nationally representative or based on structured diagnostic interviews.

3.Quality / Risk of Bias: While the methodology is clear, adding a quick summary of the risk of bias findings directly into the Results section would help readers immediately grasp the weight and reliability of the evidence being presented.

4.Elevating the GBV Discussion: The intersection of gender-based violence (GBV) and mental health is a critical point in this paper. Given the high prevalence rates reported, this section could be expanded slightly to dive deeper into the specific mental health trajectories for survivors.

5.Copyediting: A final, quick proofread would help smooth out a few awkward sentences and ensure that percentages and key technical terms are formatted consistently throughout the text.

**Reviewer #2:**

This paper presents highly ambitious and culturally tailored systemic review protocol, which involved mapping an entire country’s mental health literature across three distinct methodological dimensions (epidemiological prevalence, psychometric validation, and clinical interventions) is a massive undertaking.

The title: It reads as commentary; a direct title would be more appropriate to look like this…“child and adolescent mental health in Mozambique; a systemic review of prevalence, measurement tools and interventions” … so that it is searchable and aligned with indexing.

1. Background:

a. As in most LMICs, the scarcity of ‘some’ studies makes systemic reviews impossible and this is one of such cases. Statements about the “near absence of nationally representative data” and limited research are important but should be balanced by acknowledging existing regional surveys and emerging research efforts. The distinction between limited evidence and absence of evidence should be maintained.

b. The review does not provide nationwide epidemiological prevalence estimates; diagnostic evidence is also limited, hence the lack of nationwide representation. Hence, calling this a “nationwide” review overstates its representativeness.

c. The rationale for reviewing prevalence assessment instruments and interventions should be explained earlier. Currently, objectives appears near the end but the reason for integrating these three evidence streams is not fully developed

2. Methodology

Overall methodological recommendation: The methodology is generally appropriate and ambitious, but revisions are needed to improve transparency, reduce reviewer bias, and strengthen reproducibility.

• Rigorous Arm-Specific Frameworks: Rather than trying to force one quality appraisal tool onto three very different study designs,you appropriately decoupled your analysis using:

-COSMIN for psychometric evaluation.

-Cochrane RoB 2 and JBI for interventions.

-Using a dedicated prevalence appraisal tool.

• Review design and protocol: The use of PRISMA, PROSPERO registration, COSMIN, and Cochrane/JBI approaches strengthens methodological rigor. However, the rationale for combining prevalence, psychometric validation, and intervention studies into a single systematic review should be clarified.

• Appropriate Inclusion Boundaries: Broadly defining mental health outcomes (incorporating life adversities, bullying, and neurodevelopment) while strictly limiting the age to an average of 19 years ensures the dataset will be highly reflective of pediatric and adolescent realities in Mozambique.

1. Search strategy: The search appears comprehensive, including international databases, African sources, institutional repositories, and local journals.

• However, the full, reproducible search strategies for each database should be provided, including the search dates and the number of records retrieved.

• Moreover, the use of the “Rayyan” tool was used to “automatically [remove] pairs with an overall similarity index above 95%.” The Epidemiological Impact of this action is that it would automatically delete at a 95% threshold, which is dangerous. It frequently swallows distinct papers that share highly similar titles, such as sequential cohort updates (e.g., "Childhood Trauma in Maputo: Part I" vs. "Part II"), multi-author consortium papers, or different chapters within the same doctoral thesis.

• Best practice dictates that automated deduplication should only occur for 100% exact matches (identical DOIs or identical title/author/year strings). Anything else should be sent to a manual verification queue

2. Study selection: The use of independent dual screening at title/abstract level is appropriate, but the single-reviewer screening protocol is a concerning “high risk of selection bias” at the crucial stage of the screening process.

• You state that after title/abstract screening, “a single reviewer performed full-text screening.” Furthermore, for Google Scholar, regional databases, and local repositories, “regional and local data sources were also screened by a single author.”

• While standard systematic review guidelines (PRISMA, Cochrane) strictly recommend two independent reviewers for both title/abstract and full-text screening.

• Relying on a single reviewer, even with a 20% random verification check, significantly increases the risk of missing eligible studies (false negatives), especially when navigating local grey literature or diverse Portuguese thesis repositories.

3. Eligibility criteria: Inclusion criteria are broad and cover multiple mental health domains; however, the scope should be better structured using a framework such as PCC/PICO to improve clarity and reproducibility.

4. Risk of bias assessment: Although appropriate tools are mentioned, the manuscript should clearly report which tools were applied to each study category and how risk-of-bias judgments influenced interpretation of findings.

5. Data synthesis: Given the substantial heterogeneity of outcomes, populations, and instruments, the narrative synthesis approach is reasonable. However, the methods should describe more explicitly how studies were grouped and how conflicting evidence was handled.

6. Assessment instruments: Application of COSMIN is a methodological strength, but reporting should include clearer details on quality ratings, domains assessed, and certainty of evidence for each instrument.

7. Intervention evidence: The inclusion of RCTs and non-randomized studies is appropriate; however, conclusions regarding intervention effectiveness should be cautious because most studies had high risk of bias.

8. Reproducibility: Additional supplementary material should include the screening process, excluded full-text studies with reasons, extraction templates, and complete risk-of-bias tables to enhance transparency.

Results and Discussions

Results:

1. Organization and clarity: The results are generally well structured, covering prevalence, assessment instruments, and interventions. However, clearer separation between study characteristics, risk-of-bias findings, and main outcomes would improve readability.

2. Quantitative reporting: The review provides the number of included studies and key findings (58 prevalence reports, 35 instruments, and 7 intervention studies), it could benefit from more detailed quantitative summaries, including sample sizes, geographic distribution, study designs, and outcome estimates.

3. Prevalence findings: Reported prevalence estimates are important but should be interpreted cautiously because many findings are based on localized samples rather than nationally representative populations. Was there any an attempt made to conduct a meta-analysis to estimate a pooled prevalence of specific conditions, if data allowed?

4. Assessment instruments: The results provide useful information on cross-cultural adaptation and psychometric performance. However, presenting a summary table comparing instruments, validation properties, and quality ratings would strengthen the findings.

5. Intervention evidence: The limited number of intervention studies and high risk of bias are appropriately highlighted. The results should avoid implying effectiveness beyond what the available evidence supports.

6. Missing evidence synthesis: A clearer description of evidence gaps (e.g., disorders not studied, regions lacking data, age groups underrepresented) would improve the contribution of the results.

Discussion

1. The discussion appropriately highlights major evidence gaps and the need for improved mental health research capacity. However, some statements should be more cautious given the limited representativeness of available studies.

2. The comparison suggesting higher prevalence of mental disorders than global estimates should be interpreted carefully due to differences in sampling methods, diagnostic tools, and study settings.

3. The discussion effectively links findings to mental health system strengthening, workforce development, and research priorities. This is a major strength.

4. Limitations are discussed, including missed grey literature and heterogeneity. However, the impact of single-reviewer screening and potential publication bias should be emphasized further.

5. The conclusion should distinguish between the lack of evidence and evidence of absence. For example, the absence of studies on certain disorders (e.g., ASD, eating disorders, self-harm) reflects research gaps rather than absence of these conditions.

Overall assessment: The results and discussion provide a valuable overview of evidence on child and adolescent mental health in Mozambique, but the interpretation should be more conservative and better aligned with the limitations of the available evidence.

**Reviewer #3:**

This abstract entitled "The science of child and adolescent mental health in Mozambique: a nationwide systematic review of prevalence rates, assessment instruments, and interventions" presents a systematic review of child and adolescent mental health research in Mozambique. The study synthesizes evidence on prevalence, assessment instruments, and interventions among individuals aged 0–19 years, addressing an important gap in mental health knowledge within a low-resource setting.

Some strengths observed, leading to the above conclusions in my review are:

i. The study addresses an important Public Health gap by focusing on a largely understudied population. Mozambique has a large youth population, yet limited mental health research exists. By compiling available evidence, the review provides valuable information for policymakers, clinicians, and researchers.

ii. The Methodology utilized was comprehensive in that the authors searched multiple international and regional databases, including PubMed, Web of Science, PsycINFO, CINAHL, Google Scholar, African Index Medicus, and local catalogues. This broad search strategy increased the likelihood of identifying relevant studies and minimized publication bias.

iii. Use of Established Guidelines- Data was extracted using recognized methodological standards like COSMIN for measurement instruments and Cochrane guidance for systematic reviews, enhancing the credibility and transparency of the review.

iv. Identification of Major Mental Health Concerns- the findings reveal significant rates of anxiety, depression, ADHD, violence exposure, and suicide among Mozambican youth. These results highlight an urgent need for mental health services and preventive interventions.

v. The review not only examined prevalence but also assessed the quality of screening and diagnostic tools. Identifying culturally adapted instruments such as the MINI-KID, GAD-7, PHQ-A, and SDQ was particularly useful for future research and clinical practice. The fact that assessment instruments were evaluated gives the study more credibility.

The study also had some limitations which are outlined below: these formed the basis for the response “No” to question 2.

• It was noted that only four instruments were properly adapted and psychometrically validated. Reliance on unadapted tools could potentially affect the accuracy and validity of prevalence estimates reported in some studies.

• Although methodological frameworks were used, there is lack of detailed information regarding risk of bias, heterogeneity, or the quality of included studies. These factors are a very important consideration for interpreting the strength of evidence availed.

• Inadequate Geographic spread: The prevalence studies were conducted in Nampula and Maputo. Mozambique is geographically and culturally diverse, and findings from these regions may not reflect mental health conditions in other provinces.

• Scarcity of Intervention Studies

The review identified only seven trials. This indicates a significant lack of evidence regarding effective mental health interventions for children and adolescents in Mozambique.

• Only two studies provided clinical prevalence estimates for mental disorders. Consequently, the reported prevalence rates may not have accurately represented the entire country. This relatively low prevalence consequently also limits generalizability.

The review has major significance and implications. It demonstrates that Mozambican children and adolescents experience substantial mental health challenges alongside high levels of violence and psychosocial adversity. At the same time, there is a shortage of validated assessment tools and evidence-based interventions. The findings suggest the need for:

• Nationally representative epidemiological studies.

• Cultural adaptation and validation of mental health assessment instruments.

• Development and testing of evidence-based interventions.

• Integration of mental health services into schools and primary healthcare settings.

• Increased investment in youth mental health research and policy.

On review of the conclusion of this study, I found it to be consistent with the results presented. The evidence indicates substantial exposure to violence, abuse, and other psychosocial stressors among children and adolescents in Mozambique. The reported prevalence of anxiety, depression, and ADHD supports the assertion of a considerable mental health burden.

The review acknowledges evidence limitations- the phrase "according to limited clinical data" appropriately recognizes that prevalence estimates are based on only a small number of clinical studies. This demonstrates caution and avoids overstating the findings.

The conclusion correctly identifies the shortage of validated mental health assessment instruments which is a key research gap. However, since only a few tools were culturally adapted and psychometrically tested, this is an important limitation affecting both research and clinical practice.

The recommendation for culturally sensitive approaches is particularly important in mental health research. Assessment tools and interventions developed in high-income countries may not adequately capture local experiences, languages, and expressions of psychological distress.

It however, also had some limitations including:

i. Lack of specific recommendations:

While the conclusion calls for culturally sensitive research approaches, it does not explicitly recommend actions such as: Expanding epidemiological studies, developing intervention programs, training mental health professionals and integrating mental health services into schools and primary healthcare. Including these recommendations would strengthen the practical implications of the review.

ii. General Statement on Mental Disorder Burden

The phrase "high estimated burden" is reasonable, but because national prevalence data are limited, the wording could be interpreted cautiously. A statement such as "available evidence suggests a substantial burden" might more accurately reflect the strength of the evidence.

The conclusion is generally concise, balanced, and supported by the review findings. It effectively summarized the major messages which are pointed out below:

1. Mozambican children and adolescents experience significant psychosocial adversity.

2. Mental health disorders appear to represent an important public health concern.

3. There is a shortage of validated assessment tools.

4. More culturally appropriate research is needed.

The conclusion could be improved by providing clearer recommendations for policy, service development, and future intervention research, but it appropriately reflects the available evidence and avoids overstating the findings. The conclusion is therefore consistent with findings, very clear and acknowledges the limitations. However, it does not articulate the practical implications of the results.

Overall, this was a valuable and timely systematic review that provided the most comprehensive overview currently available; of child and adolescent mental health research in Mozambique. Its major contribution lies in highlighting both the burden of mental health problems and the substantial gaps in research infrastructure. However, the limited number of prevalence studies, validated instruments, and intervention trials means that conclusions should be interpreted cautiously. Overall, the review offers strong evidence for prioritizing child and adolescent mental health as a public health and research agenda in Mozambique. It is a very relevant study with great policy and clinical significance. It has fairly strong methodological rigor but relatively low generalizability. Evidence completeness could have been better however the study results remain very significant and valuable with critical evidence to facilitate improved clinical and community interventions.

**Reviewer #4:**

This manuscript addresses an important and underexplored area by synthesizing the available evidence on child and adolescent mental health in Mozambique. The study is notable for its nationwide scope, its focus on a population and setting where mental health research remains limited, and its effort to compile prevalence estimates, assessment instruments, and interventions in a single review. Furthermore, the review employed a comprehensive search strategy that included grey literature sources, as it improves the completeness of the evidence synthesis and helps mitigate publication bias. By bringing together these different strands of evidence, the manuscript provides a useful overview of the current state of the field and highlights important gaps for future research and service development. However, there are several matters requiring clarification.

Major comments:

1. The inclusion criteria state that the review considered "Our scope was the mental health of children and/or adolescents in Mozambique considering outcomes in a broad sense..." It would be helpful to clarify the rationale for the selection of the mental health outcomes included in the review. For example, whether these outcomes chosen based on disease burden, International Statistical Classification of Diseases, or another prespecified framework. Providing this information would improve the transparency of the review.

2. The inclusion criteria state, "We excluded multi-country studies that did not provide specific data for Mozambique, as well as general population studies without specific data for children or adolescents". However, Skar et al. (2014) appears to involve caregivers only, and adolescent-specific data are not clearly reported. The manuscript would benefit from a clearer explanation of how studies primarily targeting caregivers or teachers were handled in the eligibility criteria, particularly in relation to the stated focus on children or adolescents. Please also clarify how Skar et al. (2014) was considered eligible, as the rationale is not sufficiently clear.

3. The methods section would benefit from a clearer description of the planned synthesis approach. Specifically, please indicate whether a meta-analysis was conducted. If quantitative synthesis was not feasible, please describe the narrative synthesis methods in sufficient detail. Referring to the Synthesis Without Meta-Analysis (SWiM) reporting guideline by Campbell et al. (2020) may help ensure transparent and comprehensive reporting of synthesis without meta-analysis. In addition, please describe how the results were synthesized and presented. Providing this information would improve the transparency and reproducibility of the review and is consistent with the reporting recommendations outlined in the PRISMA guidelines.

4. The manuscript would benefit from clarification regarding whether an assessment of reporting bias or certainty of evidence was considered in the methods section, as recommended on PRISMA checklist item #14 and #15. The authors may find references by Page et al. (2022) and Schünemann et al. (2024) helpful, for guiding the implementation and reporting of these methodological components. In addition, as this is a systematic review, please indicate whether these assessments were performed; if not, a brief rationale would be appreciated, especially considering the heterogeneity of the included evidence (prevalence studies, assessment instruments, and interventions), along with a discussion of how this may affect interpretation of the findings.

5. The manuscript would benefit from a brief limitations section. In line with the PRISMA checklist, it would be helpful to discuss both the limitations of the included evidence and any limitations of the review process, as this would improve transparency and help readers interpret the findings more appropriately.

6. Based on PRISMA flow diagram provided in the supplementary material (Supplementary Figure 1), the diagram appears to contain numerical inconsistencies. Specifically, the reported numbers of records screened, records excluded, and reports assessed for eligibility do not reconcile, leaving approximately 300 records unaccounted for. As the PRISMA flow diagram is essential for ensuring transparency and reproducibility of the study selection process, please review and correct the reported figures so that all stages of screening are internally consistent.

7. The manuscript would benefit from a clearer presentation of the risk-of-bias judgments and a more precise description of the study designs in Table 4. While the use of Risk of Bias 2 (RoB 2) and Joanna Briggs Institute (JBI) critical appraisal tools is stated (page 7–8), reporting the study-level or domain-level assessments would strengthen transparency and help readers interpret the findings more confidently. In addition, the included evidence appears to comprise cross-sectional and mixed-methods studies alongside controlled and uncontrolled designs. Clearer classification of study design is important because it allows readers to see whether the appropriate critical appraisal tool was applied to each study type, thereby strengthening the transparency, consistency, and credibility of the quality assessment.

8. The discussion would benefit from a more critical and balanced interpretation of the findings. At present, it largely repeats the results and focuses on implications and future research, but it does not sufficiently engage with previous studies, including those with conflicting or contrasting results. In addition, it does not address risk of bias, heterogeneity, differences in study design, or the strength of the evidence. Strengthening the discussion with more direct support from the literature and aligning the conclusions more closely with the scope and quality of the included evidence would improve the credibility and balance of the manuscript.

Minor comments:

1. Overall, the manuscript is well organized. However, I believe it would benefit from a careful revision of the writing to improve the flow and readability, particularly in the Results and Discussion sections. In several places, the presentation is quite concise, making it difficult to fully appreciate the progression of the findings and their interpretation. Expanding key explanations where appropriate and improving the transitions between ideas would strengthen the overall presentation.

2. The readability of the manuscript could also be improved by organizing the presentation of the included studies more systematically. For example, Table 1 might be easier to follow if the studies were grouped by sample recruitment setting (e.g., nationwide, regional, then school-based) and, where appropriate, by diagnostic criteria. Similarly, Table 2 could be organized by recruitment setting and type of adversity exposure, while Table 4 might benefit from grouping studies according to intervention target and setting. A more consistent organization across the tables may help readers identify patterns and compare studies more easily.

3. The study selection process is described inconsistently between the main text and Supplementary Table 2. Page 6 states that two independent authors conducted title/abstract screening and a single reviewer performed full-text screening, whereas Supplementary Table 2 indicates that two independent reviewers conducted both primary and secondary screening (title/abstract and full-text screening). Please reconcile these descriptions and clarify the actual screening procedure used.

4. According to PRISMA 2020 item #8, the review should report how automation tools were integrated into the study selection process. If records were removed by automation before screening, this should be shown in the PRISMA flow diagram as “Records marked as ineligible by automation tools,” similar to how deduplication is reported.

5. The eligibility criteria state that studies were included if they reported specific data for samples in Mozambique with an average age up to 19 years old, regardless of outliers. However, on Table 1, 2, and 4, several included studies appear to involve age ranges extending beyond 19 years. To avoid potential confusion, it would be helpful to report the mean age for each study, if available, along with the standard deviation, rather than only the age range.

6. In the page 7–8 “Methodological quality was assessed with the revised Cochrane tool for randomized trials (RoB 2) and the Joanna Briggs Institute (JBI) checklist for non-randomized designs.” Please provide additional details on who performed the assessments, whether reviewers worked independently, how disagreements were resolved, and how the tool outputs were incorporated into the review process, as recommended by PRISMA.

7. In Table 4, the “effects” column is unclear. Please specify what effect was measured for each intervention. If it is regarding the effect of the interventions, please report the corresponding estimate and precision measures, such as the point estimate, 95% confidence interval, and p value where available, rather than only stating “statistically significant improvement...”. Additionally, Table 4 should include a note explaining the meaning of the symbols “+” and “?” used in the effect column. At present, the table is not fully interpretable without a legend defining these symbols.

8. In Page 28, second paragraph, the study being discussed should be cited consistently every time it is mentioned. Please add the relevant reference after each statement derived from that study to improve clarity and ensure proper attribution. The same issue also appears in Page 30, second paragraph, where the study should likewise be cited consistently throughout the discussion.

9. In page 29, first paragraph, the statement “Similarly, other register-based studies in Mozambique are scarce and rely primarily on service-level data from isolated facilities, limiting their generalizability” should be cited. Please add the relevant reference to support this claim.

10. In the page 31, the conclusion appears stronger than the evidence presented. While the review suggests an emerging field and important gaps in screening tools and culturally adapted instruments, the certainty of evidence is not clearly summarized, and the strength of these claims should be tempered accordingly. Please align the conclusion more closely with the quality and certainty of the underlying evidence and explicitly acknowledge the limitations of the included studies.

References:

Campbell, M., McKenzie, J. E., Sowden, A., Katikireddi, S. V., Brennan, S. E., Ellis, S., Hartmann-Boyce, J., Ryan, R., Shepperd, S., Thomas, J., Welch, V., & Thomson, H. (2020). Synthesis without meta-analysis (SWiM) in systematic reviews: Reporting guideline. BMJ, 368, l6890. https://doi.org/10.1136/bmj.l6890

Page, M. J., Higgins, J. P. T., & Sterne, J. A. (2022). Chapter 13: Assessing risk of bias due to missing evidence in a meta-analysis | Cochrane. In J. P. T. Higgins, J. Thomas, J. Chandler, M. Cumpston, T. Li, & M. J. Page (Eds.), Cochrane Handbook for Systematic Reviews of Interventions. Cochrane. https://www.cochrane.org/authors/handbooks-and-manuals/handbook/current/chapter-13

Schünemann, H. J., Higgins, J. P. T., Vist, G. E., Glasziou, P., Akl, E. A., Skoetz, N., & Guyatt, G. H. (2024). Chapter 14: Completing ‘Summary of findings’ tables and grading the certainty of the evidence [last updated August 2023]. In J. Higgins, J. Thomas, J. Chandler, M. Cumpston, T. Li, & M. Page (Eds.), Cochrane Handbook for Systematic Reviews of Interventions version 6.5. Cochrane. cochrane.org/handbook.

Skar, A.-M. S., Sherr, L., Clucas, C., & von Tetzchner, S. (2014). Evaluation of Follow-Up Effects of the International Child Development Programme on Caregivers in Mozambique. Infants & Young Children, 27(2), 120–135. https://doi.org/10.1097/IYC.0000000000000006

**********

Figure Resubmissions:

# Author comment: Author response to Decision Letter 1

**DOI:** 10.1371/journal.pgph.0007513.r002

13 Aug 2026

Attachment Submitted filename: Response to reviewers.docx

# Peer review: Decision Letter 1

**DOI:** 10.1371/journal.pgph.0007513.r003

**Authors:** Nawi Ng

8 Sep 2026

PGPH-D-26-01930R1

The science of child and adolescent mental health in Mozambique: a systematic review of prevalence estimates, measurement tools, and interventions

PLOS Global Public Health

Dear Dr. Marchinatti,

Thank you for submitting your manuscript to PLOS Global Public Health. After careful consideration, we feel that it has merit but does not fully meet PLOS Global Public Health’s publication criteria as it currently stands. Therefore, we invite you to submit a revised version of the manuscript that addresses the points raised during the review process.

Please submit your revised manuscript by 28 September 2026. If you will need more time than this to complete your revisions, please reply to this message or contact the journal office at globalpubhealth@plos.org. When you're ready to submit your revision, log on to https://www.editorialmanager.com/pgph/ and select the 'Submissions Needing Revision' folder to locate your manuscript file.

Please include the following items when submitting your revised manuscript:

- A letter that responds to each point raised by the editor and reviewer(s). You should upload this letter as a separate file labeled 'Response to Reviewers'.
- A marked-up copy of your manuscript that highlights changes made to the original version. You should upload this as a separate file labeled 'Revised Manuscript with Track Changes'.
- An unmarked version of your revised paper without tracked changes. You should upload this as a separate file labeled 'Manuscript'.

Guidelines for resubmitting your figure files are available below the reviewer comments at the end of this letter.

As the corresponding author, your ORCID iD is verified in the submission system and will appear in the published article. PLOS supports the use of ORCID, and we encourage all coauthors to register for an ORCID iD and use it as well. Please encourage your coauthors to verify their ORCID iD within the submission system before final acceptance, as unverified ORCID iDs will not appear in the published article. *Only*  the individual author can complete the verification step; PLOS staff *cannot*  verify ORCID iDs on behalf of authors.

We look forward to receiving your revised manuscript.

Kind regards,

Nawi Ng

Academic Editor

PLOS Global Public Health

**Journal Requirements:**

If the reviewer comments include a recommendation to cite specific previously published works, please review and evaluate these publications to determine whether they are relevant and should be cited. There is no requirement to cite these works unless the editor has indicated otherwise.

Please review your reference list to ensure that it is complete and correct. If you have cited papers that have been retracted, please include the rationale for doing so in the manuscript text, or remove these references and replace them with relevant current references. Any changes to the reference list should be mentioned in the rebuttal letter that accompanies your revised manuscript. If you need to cite a retracted article, indicate the article’s retracted status in the References list and also include a citation and full reference for the retraction notice.

**Additional Editor Comments (if provided):**

[Note: HTML markup is below. Please do not edit.]

Reviewers' comments:

Reviewer’s Responses to Questions

**Comments to the Author**

Reviewer #1: All comments have been addressed

Reviewer #3: All comments have been addressed

Reviewer #4: All comments have been addressed

**********

publication criteria?

Reviewer #1: Yes

Reviewer #3: Yes

Reviewer #4: Yes

**********

3. Has the statistical analysis been performed appropriately and rigorously?

Reviewer #1: Yes

Reviewer #3: Yes

Reviewer #4: N/A

**********

4. Have the authors made all data underlying the findings in their manuscript fully available (please refer to the Data Availability Statement at the start of the manuscript PDF file)?

The PLOS Data policy

Reviewer #1: Yes

Reviewer #3: Yes

Reviewer #4: Yes

**********

5. Is the manuscript presented in an intelligible fashion and written in standard English?

Reviewer #1: Yes

Reviewer #3: Yes

Reviewer #4: Yes

**********

**Reviewer #1** : I have made substantial revisions to the manuscript and have adequately addressed the major concerns raised in the previous round of review. The revised manuscript is clearer and more transparent, particularly regarding the search strategy, study selection, risk of bias assessment, reporting of the findings, and discussion of limitations.

I have only a few minor points that I recommend addressing before publication:

1. The authors should provide a little more clarity regarding the single reviewer screening process and the extent to which the 20% independent verification identified any disagreements or additional eligible studies.

2. Given the limited number and geographical concentration of studies providing clinical prevalence estimates, the authors should continue to be cautious when discussing the overall burden of mental disorders in Mozambique and clearly distinguish between lack of evidence and evidence of absence.

3. The limitations associated with heterogeneity of study designs, assessment instruments, and populations should remain clearly reflected when interpreting and comparing prevalence estimates.

These points do not substantially affect the overall value or conclusions of the review and should be relatively straightforward to address.

Overall, the manuscript has been considerably improved and provides a valuable synthesis of the available evidence on child and adolescent mental health in Mozambique. I believe it is suitable for publication following these minor revisions.

**Reviewer #3** : This research report is exceptionally well-structured, thorough, and insightful. The author demonstrates a clear understanding of, with strong methodology, relevant data, and logical analysis.

What I found most impressive was the clarity of presentation - complex findings are explained in an accessible way, supported by credible sources and well-organized evidence. The conclusions are well-grounded and offer practical implications.

It is a valuable contribution to the field and I would highly recommend it to anyone interested in child and adolescent psychiatry. It’s an excellent work.

Strengths:

1. Relevance & Originality:* The report addresses a timely and important problem in.. Methodology:* The research design is sound, rigorous, and appropriate for the research questions.

3. Analysis & Clarity:Data is analyzed comprehensively and presented with great clarity. Figures/Tables are effective.

4. Conclusion: Findings are well-supported and the recommendations are actionable.

Overall, this is a high-quality report that reflects deep research and critical thinking. It meets academic and professional standards with distinction.

An excellent and well-researched report! Very informative, well-organized, and data-driven. The author did a fantastic job breaking down into clear, actionable insights. Highly recommended.

**Reviewer #4:**  The revised manuscript has been considerably improved, and the authors are to be commended for their careful attention to the comments raised in the previous round. This review addresses an important and underexplored area of child and adolescent mental health in Mozambique, and its nationwide scope, comprehensive search strategy, inclusion of grey literature, and synthesis of prevalence estimates, assessment instruments, and interventions remain important strengths of the study. The revisions have also improved the clarity and overall presentation of the manuscript. Most of the concerns raised previously have been adequately addressed, with only a few minor points remain for consideration. The following minor points are suggested for further consideration:

1. The authors explain in their response that a formal certainty-of-evidence assessment was not conducted given the substantial heterogeneity across the included studies. However, the implications of this heterogeneity for the interpretation of the findings should be more clearly reflected in the manuscript. I suggest that the authors explicitly describe the main sources of heterogeneity across the studies (e.g., differences in outcome definitions or assessment instruments). This would help readers understand that the reported findings should be interpreted with caution and may not be directly comparable or conclusive.

2. There appears to be lack of clarity in the reporting of the number of studies, reports, and publications. For example, in the page 9, “…61 prevalence reports are described as comprising 15 studies on mental health disorders, 9 on substance use, 15 on psychosocial adversities, and 9 on other psychosocial symptoms”, which sum to 48. In addition, the manuscript reports 29 reports on assessment instruments and 9 reports describing intervention trials, while the total number of unique publications is stated as 81. Please clarify how these numbers relate to one another, including the distinction between a study, report, and publication, and ensure that the numbers are consistent throughout the manuscript. It may also be helpful to add the corresponding number of studies for each domain or theme in the PRISMA flow diagram, so that readers can more easily follow how the reports relate to the total number of unique studies.

3. There are some discrepancies that would benefit from clarification in the reporting of the numbers throughout the manuscript and PRISMA flow diagram. In the PRISMA diagram, 451 reports are indicated as excluded, whereas the numbers provided by the different reasons for exclusion appear to sum to 440, leaving 11 reports unaccounted for. Please clarify this discrepancy.

4. In addition, the PRISMA diagram (supplementary material) reports 81 reports included in the review, while only 45 studies are reported as being included. While multiple reports may originate from the same study, the relationship between reports and studies should be made clearer. For example, where multiple reports relate to the same study or represent follow-up or subsequent publications from the same study, it would be helpful to clarify how these were identified. This would help readers understand how the 81 reports were consolidated into 45 studies and ensure that the included evidence is not double counted.

5. Figure 2 (page 10) presents the availability of evidence-based resources across the different domains of inquiry. However, the figure is not sufficiently described in the main text. It would be helpful to briefly summarize the key findings presented in the figure, rather than leaving the interpretation entirely to the reader.

6. For the non-randomized studies, the supplementary file provides the Yes/No/Unclear/Not Applicable responses to the JBI checklist. Given that the critical appraisal was conducted by a single reviewer (page 7), it would be helpful if the authors could consider adding a “Justification” column, as provided in the JBI checklist, to briefly explain the basis for each judgement of each question. This would further enhance the transparency of the appraisal process and allow readers to better understand how the judgements were made.

**********

**Figure Resubmissions:**

# Author comment: Author response to Decision Letter 2

**DOI:** 10.1371/journal.pgph.0007513.r004

17 Sep 2026

Attachment Submitted filename: 2nd Response to reviewers.docx

# Peer review: Decision Letter 2

**DOI:** 10.1371/journal.pgph.0007513.r005

**Authors:** Nawi Ng

5 Oct 2026

The science of child and adolescent mental health in Mozambique: a systematic review of prevalence estimates, measurement tools, and interventions

PGPH-D-26-01930R2

Dear Dr Marchinatti,

We are pleased to inform you that your manuscript 'The science of child and adolescent mental health in Mozambique: a systematic review of prevalence estimates, measurement tools, and interventions' has been provisionally accepted for publication in PLOS Global Public Health.

Before your manuscript can be formally accepted you will need to complete some formatting changes, which you will receive in a follow up email. Please address Reviewer 4’s remaining comments at that stage as well. A member of our team will be in touch with a set of requests.

Please note that your manuscript will not be scheduled for publication until you have made the required changes, so a swift response is appreciated.

IMPORTANT: The editorial review process is now complete. PLOS will only permit corrections to spelling, formatting or significant scientific errors from this point onwards. Requests for major changes, or any which affect the scientific understanding of your work, will cause delays to the publication date of your manuscript.

If your institution or institutions have a press office, please notify them about your upcoming paper to help maximize its impact. If they'll be preparing press materials, please inform our press team as soon as possible -- no later than 48 hours after receiving the formal acceptance. Your manuscript will remain under strict press embargo until 2 pm Eastern Time on the date of publication. For more information, please contact globalpubhealth@plos.org.

Thank you again for supporting Open Access publishing; we are looking forward to publishing your work in PLOS Global Public Health.

Best regards,

Nawi Ng

Academic Editor

PLOS Global Public Health

***********************************************************

Reviewer Comments (if any, and for reference):

Reviewer’s Responses to Questions

**Comments to the Author**

Reviewer #1: All comments have been addressed

Reviewer #4: All comments have been addressed

**********

publication criteria?

Reviewer #1: Yes

Reviewer #4: Yes

**********

3. Has the statistical analysis been performed appropriately and rigorously?

Reviewer #1: Yes

Reviewer #4: Yes

**********

4. Have the authors made all data underlying the findings in their manuscript fully available (please refer to the Data Availability Statement at the start of the manuscript PDF file)?

The PLOS Data policy

Reviewer #1: Yes

Reviewer #4: Yes

**********

5. Is the manuscript presented in an intelligible fashion and written in standard English?

Reviewer #1: (No Response)

Reviewer #4: Yes

**********

Reviewer #1: Overall, the revised manuscript is substantially improved and provides a useful catalogue of the available evidence. The limitations are appropriately acknowledged, and the conclusions are broadly consistent with the evidence presented. I have no major methodological concerns at this stage. The remaining issues are minor and primarily concern clarity and consistency.

Reviewer #4: Thank you for the authors continued efforts to improve the manuscript throughout the revision process. The authors have carefully addressed the previous comments, and the manuscript has improved substantially as a result. The revisions have strengthened the clarity, organization, and overall presentation of the study. The paper is now in very good shape, with a clear and logical structure, a coherent presentation of the methods and findings, and an engaging discussion of the study’s relevance.

I have only a few minor points remaining, mainly to further enhance clarity and ensure consistency in formatting before publication:

1. Table footnotes: Please ensure that all abbreviations referring to diagnostic criteria, instruments, or assessment tools used in the tables (e.g., PHQ-9 and GAD-7) are written in full and clearly defined in the relevant table footnotes. This will help ensure that each table is fully self-explanatory, particularly for readers from a broader public health audience.

2. Proofreading and formatting: Please conduct one final, careful proofreading of the manuscript to identify and correct any remaining typographical errors and formatting inconsistencies throughout the text, tables, figures, and references. Please ensure consistency in capitalization, terminology, abbreviations, punctuation, spacing, and the overall presentation of the manuscript (e.g., the capitalization of academic titles such as “PhD” in the page 11).

These are minor revisions only. Addressing them will further polish an already well-structured and valuable manuscript, and I believe the paper will make a useful contribution to the public health literature.

**********
